# Association of systemic inflammatory markers with clinical adverse prognosis and outcomes in HFpEF: a systematic review and meta-analysis of cohort studies

**DOI:** 10.3389/fcvm.2024.1461073

**Published:** 2024-09-30

**Authors:** Zhenyue Fu, Pengfei Liu, Xiya Gao, Shuqing Shi, Yumeng Li, Bingxuan Zhang, Huaqin Wu, Qingqiao Song

**Affiliations:** ^1^ Graduate School, Beijing University of Chinese Medicine, Beijing, China; ^2^ Department of General Internal Medicine, Guang'anmen Hospital, China Academy of Chinese Medical Sciences, Beijing, China; ^3^ Department of Cardiology, Guang'anmen Hospital, China Academy of Chinese Medical Sciences, Beijing, China

**Keywords:** inflammation, systematic review, meta-analysis, heart failure with preserved ejection fraction, neutrophil-to-lymphocyte ratio

## Abstract

**Objective:**

To evaluate the association between systemic inflammatory markers and clinical outcomes (all-cause mortality, cardiovascular mortality, and rehospitalization) in patients with heart failure with preserved ejection fraction (HFpEF).

**Methods:**

We conducted a comprehensive literature search in PubMed, Embase, and Ovid Medline databases from inception to June 27, 2024. Studies were included if they were observational clinical studies involving HFpEF patients over 18 years old, with exposure to systemic inflammatory markers and reporting on adverse prognosis outcomes. The Newcastle-Ottawa Scale (NOS) was used to assess study quality.

**Results:**

Eight studies ultimately included in the meta-analysis which involved 9,744 participants from six countries. The meta-analysis showed that systemic inflammatory markers were significantly associated with all-cause mortality (HR 1.43, 95% CI 1.19–1.72, *p* < 0.05), cardiovascular mortality (HR 2.04, 95% CI 1.33–3.12, *p* < 0.05), and cardiovascular rehospitalization (HR 2.83, 95% CI 0.92–8.67, *p* < 0.05) in HFpEF patients. Low heterogeneity was observed across studies (I^2^ = 0.00%). Sensitivity and publication bias analyses indicated that the results were robust.

**Conclusion:**

Systemic inflammatory markers demonstrate significant predictive value for adverse clinical outcomes in HFpEF patients. The findings suggest that monitoring systemic inflammation may provide valuable prognostic information for clinicians managing HFpEF patients.

**Systematic Review Registration:**

https://www.crd.york.ac.uk/PROSPERO/display_record.php?RecordID=562698, identifier (CRD42024562698).

## 1 Introduction

With the acceleration of the aging process, the incidence of heart failure continues to rise, becoming a major challenge in the field of global public health. Epidemiological cohort studies show that the prevalence of heart failure with preserved ejection fraction (HFpEF) was 47.8% between 2000 and 2003, then rose to 56.9% between 2004 and 2007, and 52.3% between 2008 and 2010, highlighting its status as the main subtype of heart failure (1, 2). Similarly, the incidence of HFpEF has also been rising in the past 20 years, with an estimated incidence of approximately 27 cases per 10,000 person-years (3, 4).Unlike heart failure with reduced ejection fraction (HFrEF), the pathophysiological mechanisms of HFpEF are more complex and closely related to the overlapping heterogeneity of its causes, such as aging, obesity, atrial fibrillation, diabetes, sarcopenia, etc (5–8). Modern scientific research teams have long been committed to exploring the basic pathophysiological mechanisms of chronic heart failure (CHF). The MIMICA study revealed the potential inflammation and metabolic imbalance of anabolism and catabolism in CHF (9). In the CANTOS cohort, the application of the IL-1β monoclonal antibody Canakinumab significantly reduced the risk of cardiovascular events after myocardial infarction, a finding that for the first time confirmed the potential benefits of anti-inflammatory treatment in cardiovascular diseases (10). The SATELLITE trial further revealed that myeloperoxidase inhibition can inhibit the activation of inflammatory factors and the transmission of cardiac hypertrophy signals, improving the exercise tolerance and quality of life of HFpEF patients (11, 12). The LoDoCo and LoDoCo2 trials indicate that patients receiving 0.5 mg of colchicine once daily exhibit a reduced risk of acute cardiovascular events compared to those not receiving colchicine (13). This effect may be achieved by inhibiting the release of neutrophil enzymes and the assembly and activation of the NLRP3 inflammasome, thereby downregulating the concentrations of Interleukin-6 (IL-6) and C-reactive protein (CRP) (14).

The infiltration of the inflammatory microenvironment is an indispensable part of the evolution of CHF. The inflammation in HFrEF originates from myocardial injury, whereas the inflammation in HFpEF is triggered by systemic metabolic and inflammatory risk factors such as obesity, hypertension, and diabetes. Chronic systemic inflammation may induce endothelial dysfunction through mechanisms involving oxidative stress and NO bioavailability imbalance, leading to myocardial infiltration with activated leukocytes, cardiac hypertrophy, fibrosis, and myocardial stiffness, eventually resulting in progressive diastolic dysfunction (15). Vascular endothelial injury may lead to neutrophil migration (16) while myocardial infiltration is associated with increased monocyte/macrophage recruitment and aggravated fibrosis (17). It suggests the elevated levels of serum inflammatory cells in HFpEF patients, but the predictive power for the prognosis of HFpEF patients still needs further evaluation (18, 19).

This study selected inflammatory markers available from complete blood count, such as high vs. low neutrophil-to-lymphocyte ratio (NLR), lymphocyte-to-monocyte ratio (LMR), platelet-to-lymphocyte ratio (PLR), etc., which can comprehensively reflect the body's inflammation status. Although there is abundant evidence to confirm the role of inflammation and immune activation in promoting CHF, while in the clinical disease process it still lacks evidence-based support. Previous meta-analyses have explored the association between systemic inflammatory markers and coronary artery disease (CAD) and stroke (20, 21), and revealed their diagnostic and prognostic value. Currently, many related clinical studies have also found an association between inflammatory markers and adverse clinical outcomes of HFpEF. Therefore, we have conducted a systematic review and meta-analysis to rigorously assess their predictive utility by synthesizing existing evidence.

## 2 Materials and methods

### 2.1 Search strategy and inclusion criteria

This study's design and reporting strictly adhered to the Preferred Reporting Items for Systematic Reviews and Meta-Analyses (PRISMA) guidelines (22) (Supplementary Table S1) and was duly registered in the International Prospective Register of Systematic Reviews (Registration number: CRD42024562698). The process of data retrieval, extraction, and analysis was undertaken by FZY and LPF. In instances where disagreements arose, consensus was reached by consulting with SQQ.

We searched PubMed/Medline and Embase from the inception of the databases up to June 27, 2024, to identify literature related to the prognostic value of systemic inflammatory markers in HFpEF. In accordance with the PICOS framework, we employed a comprehensive search strategy combining thematic word and free word, including HFpEF, neutrophil-to-lymphocyte ratio, Leukocytes, Neutrophils, Lymphocytes, C-Reactive Protein, etc. The specific search strategy can be found in the Supplementary Table S2.

If the research articles meet the following criteria, they are included: (1) Population: Participants diagnosed with HFpEF and are over 18 years old. (2) Exposure: Systemic inflammatory markers available from complete blood count, including neutrophil-to-lymphocyte ratio (NLR), lymphocyte-to-monocyte ratio (LMR), platelet-to-lymphocyte ratio (PLR), white blood cells (WBC), platelets, lymphocytes, and CRP, HDL(high-density lipoprotein)/CRP ratio. They are divided into high and low groups based on the cutoff values, representing exposure to different levels of inflammatory states. (3) Outcome measures: The hazard ratios (HR) or adjusted HRs for the occurrence of adverse outcomes in HFpEF (all-cause mortality/cardiovascular mortality/rehospitalization rate). 4) Study design: cohort studies.

We excluded: (1) Reviews, conference abstracts, systematic reviews, meta-analyses, and case reports; (2) Non-English studies; (3) Studies with a follow-up period of less than 365 days.

### 2.2 Data extraction

We recorded the following information in the standardized data extraction form in Microsoft Excel 2019: (1) Basic information: first author's name, country/region, year of publication, clinical trial registration number, study design; (2) Baseline information: sample size, gender ratio, average age, medication history; (3) Measured outcomes: reported systemic inflammatory marker measurements (mean and standard deviation), cutoff values and the basis for the cutoff (median, quartile, ROC), HRs and adjusted HRs for HFpEF clinical outcomes (all-cause mortality, cardiovascular mortality, readmission rate).

### 2.3 Study quality assessment

The Newcastle-Ottawa Scale (NOS) was used to assess each study in the following three aspects: the selection of the study population, the comparability of the groups, and the outcomes of non-randomized studies. The total score is 9 points, where high-quality studies score 7–9 points, medium-quality studies score 4–6 points, and low-quality studies score less than 4 points (23, 24).

### 2.4 Data analysis

Data analysis was conducted using Stata 17.0 (Stata Corporation, College Station, TX, USA) and R 4.2.0. The Cochran's Q statistic and the I^2^ statistic were used to assess heterogeneity (25). If I^2^ > 50%, it indicates significant heterogeneity, and a random-effects model was used to combine the effect sizes; otherwise, a fixed-effects model was used (26, 27). The adjusted HRs for the interest outcomes were extracted to calculate the pooled estimates and 95% confidence intervals (CI). Sensitivity analysis was assessed in the following two ways: (1) leave-one-out analysis: to identify the study that most affects the robustness of the results (28), and to observe whether the exclusion of that study affects the direction of the estimates; (2) altering the effect sizes combined model: The impact of model selection on the results can be assessed by alternating fixed-effect and random-effect models in the meta-analysis. If the conclusions are significantly different, this may indicate that interstudy heterogeneity has an important influence on the results.

Publication bias was assessed in the following three ways: (1) Contour-enhanced funnel plot: to observe its symmetry and the range it spans (29); (2) Trim and fill method: to observe whether the direction of the correlation coefficients changes through iterative procedures (30); (3) Egger's test and Begg's test. Since the number of studies included in this meta-analysis is less than 10, Egger's test or Begg's test will not be conducted according to the recommendations of the Cochrane Handbook.

## 3 Results

### 3.1 Basic characteristics of included studies

**Figure 1:**
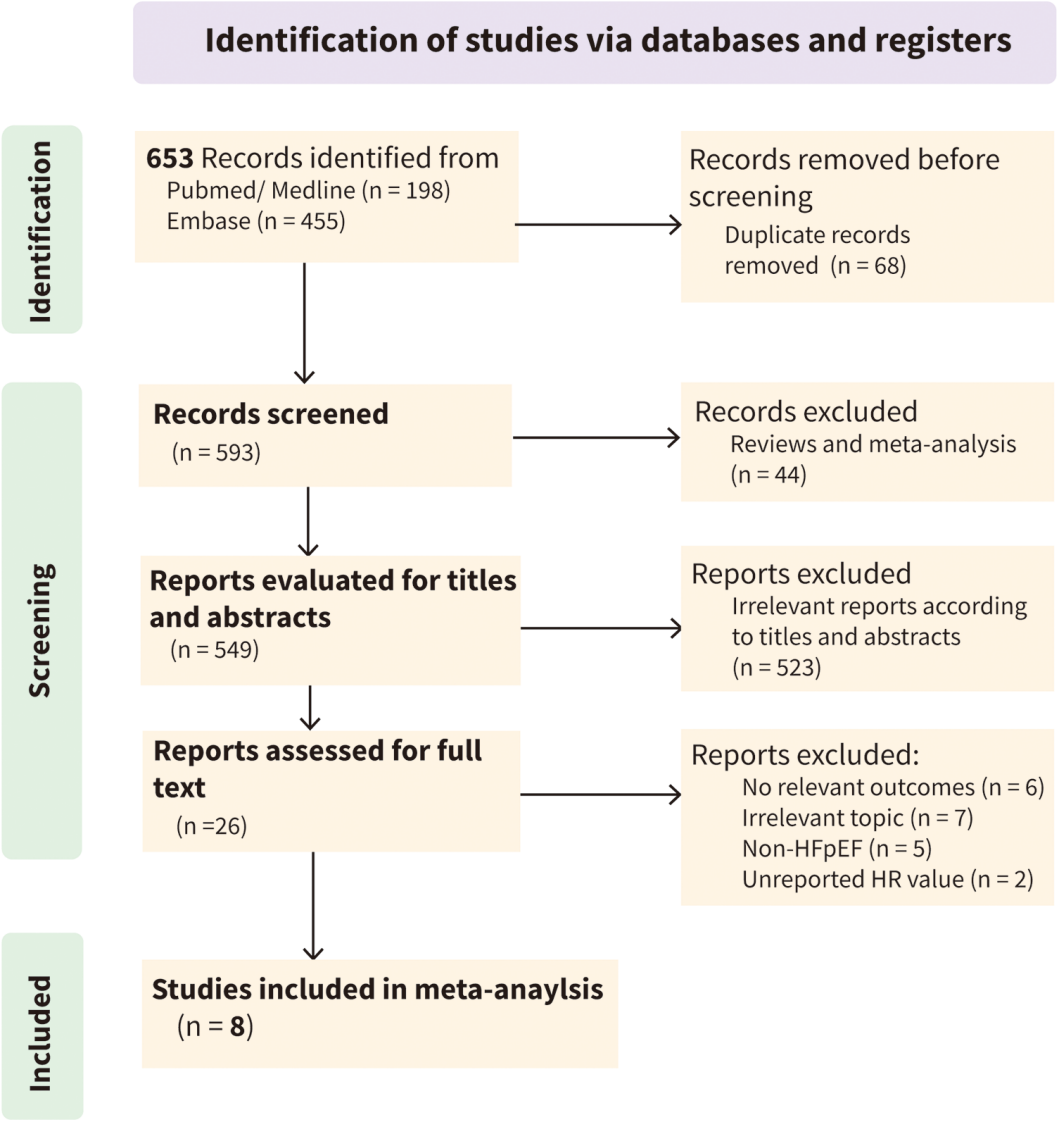
Flow diagram of studies selection process.

According to the established search strategy, 653 articles were initially identified from three databases. After automatic deduplication using EndNote software, 593 articles remained. After excluding irrelevant literature by reading titles, abstracts, and full texts, eight studies were included in the final meta-analysis (Figure 1 and Supplementary Table S3).

The eight studies included in the final quantitative analysis examined the association between systemic inflammatory markers and adverse clinical outcomes in HFpEF. All studies were published in the last five years (2020–2024) and were conducted in China (*n* = 2) (31, 32), Japan (*n* = 2) (33, 34), the United States (*n* = 1) (35), the United Kingdom (*n* = 1) (36), Belgium (*n* = 1) (37), and France (*n* = 1) (38). All studies were prospective cohort studies, including a total of 9,744 participants with an average age of 71.43. Systemic inflammatory markers included NLR, PLR, HDL/CRP, hs-CRP, WBC, and platelets. The sample size of the cohorts ranged from 232 to 3,459, and the follow-up time varied from 420 days to 3.4 years. The NOS scale showed that all studies were of high quality (Table 1).

**Table 1:** Basic information of included studies.

Author/year	Nation	Registration No.	No. (male)	Age (mean + sd)	Phenotype	Index	Cut-off type	Medication history	endpoints	Follow-up duration	Sensitivity,%	Specificity,%	NOS
Tamaki et al. (33)	Japan	UMIN000021831	1,026 (45%)	83 (5)	ADHF with preserved ejection fraction	NLR, PLR	ROC curve low NLR(≤4.5)and PLR(≤193)(*n* = 492) high NLR(>4.5)or high PLR(>193)(*n* = 242) high NLR and PLR(*n* = 292)	Loop diuretics 50.0% ACEi/ARBs 50.0% *β*-blockers 46.0% MRAs 21.0% SGLT2 Inhibitors 2.0% Statins 30.0%	Cardiac death *n* = 85 All-cause death *n* = 110	429d	Cardiac death 49 All-cause death 73	Cardiac death 43 All-cause death 75	7
Yano et al. (42)	Japan	NA	796 (45%)	82 (6)	ADHF with preserved ejection fraction	HDL/CRP	ROC curve HDL-C/CRP 4.05	Loop diuretics 46.0% ACEi/ARBs 45.0% β-blockers 54.9% MRAs 21.5% Statins 30.0%	Cardiac death *n* = 51 All-cause death *n* = 118	420d	Cardiac death 78.4 All-cause death 79.7	Cardiac death 44.6 All-cause death 38.2	7
Ferreira at al. (38)	France	NCT00094302	232	73 (9)	HFpEF	hs-CRP	Median hsCRP < 2 mg/L (*n* = 89) hsCRP>=2 mg/L (*n* = 143)	Loop diuretics 89.3% ACEi/ARBs 75.2% β-blockers 82.5%	Cardiac death *n* = 51 All-cause death *n* = 146 heart failure hospitalization *n* = 34	3.3y	NA	NA	8
Zhou et al. (31)	China	NA	3,459 (61%)	65.8 (10.7)	HFpEF With MAFLD and Suspected Coronary Artery Disease	hs-CRP	Quartile Q1: ≤3.26 mg/L Q2: 3.26–7.00 mg/L Q3: 7.01–36.9 mg/L Q4: >36.9 mg/L	Loop diuretics 18.3% ACEi/ARB/ARNIs 62.9% β-blockers 65.7% MRAs 14.0% SGLT2 Inhibitors 1.2%	heart failure hospitalization *n* = 598	3.2y	NA	NA	7
Zhu and Zhou (32)	China	NA	2,898 (46%)	69 (9.6)	HFpEF	WBC	Quartile Q1: ≦ 5.5 × 10^9^/L Q2: > 5.5 × 10^9^/L to ≦ 6.7 × 10^9^/L Q3: > 6.7 × 10^9^/L to ≦ 8.0 × 10^9^/L Q4: > 8.0 × 10^9^/L	Aspirin 53.9% β-blockers 65.6% ACEi/ARBs 67.6% Statins 42.5% CCB 32.8% Spironolactone 42.5% Loop diuretic 42.7% Thiazide diuretic 32.2%	All-cause death *n* = 429 heart failure hospitalization *n* = 386	3.4y	NA	NA	8
Menghoum et al. (37)	Belgium	NCT03197350	228	79 (9)	HFpEF	platelets	Quartile	ACEi/ARBs 62.0% β-blockers 59.4% Loop diuretics 60.5% Thiazide 16.5% MRA 19.5% CCB 36.1% Anticoagulants 53.0% Antiplatelet agents 29.7% Statins 41.7%	All-cause death *n* = 87 heart failure hospitalization *n* = 107	26m	NA	NA	9
Curran et al. (36)	UK	NA	662	74 (10)	HFpEF	NLR	Tertiles NLR 3.22	ACEI/ARB 70.8% β-blockers 73.2% MRA 32.6% Loop diuretics 97.0%	NA	18m	NA	NA	7
Boralkar et al. (35)	USA	NA	443	77 (16)	acute HFpEF	NLR	Tertiles	ACEI/ARB 42.4% β-blockers 57.3% diuretics 59.6%	All-cause death *n* = 121	2.2y	NA	NA	8

### 3.2 Meta-Analysis results

#### 3.2.1 Prognostic value of systemic inflammatory markers for All-cause mortality in HFpEF

**Figure 2:**
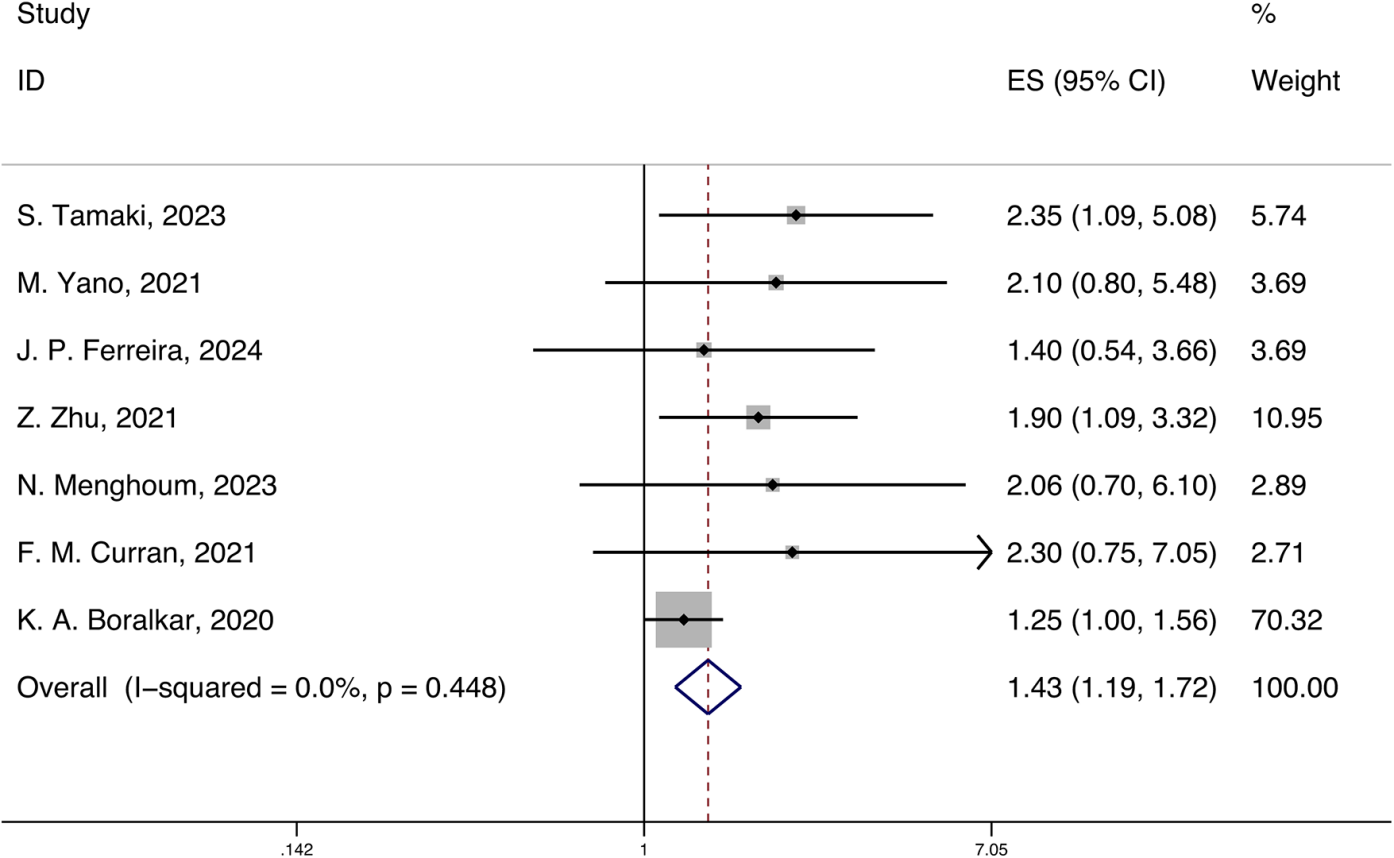
Forest plot and meta-analysis of systemic inflammatory markers and all-cause mortality in HFpEF.

A total of 7 studies reported the HRs and 95% CIs for the association of systemic inflammatory markers with all-cause mortality in HFpEF, with follow-up periods ranging from 420 days to 3.6 years. Using a fixed-effects model for meta-analysis, the results indicated that systemic inflammatory markers are correlated with all-cause mortality in HFpEF [HR 1.43 (1.19, 1.72), *p* < 0.05, I2 = 0.00%] (Figure 2).

#### 3.2.2 Prognostic value of systemic inflammatory markers for cardiovascular mortality in HFpEF

**Figure 3:**
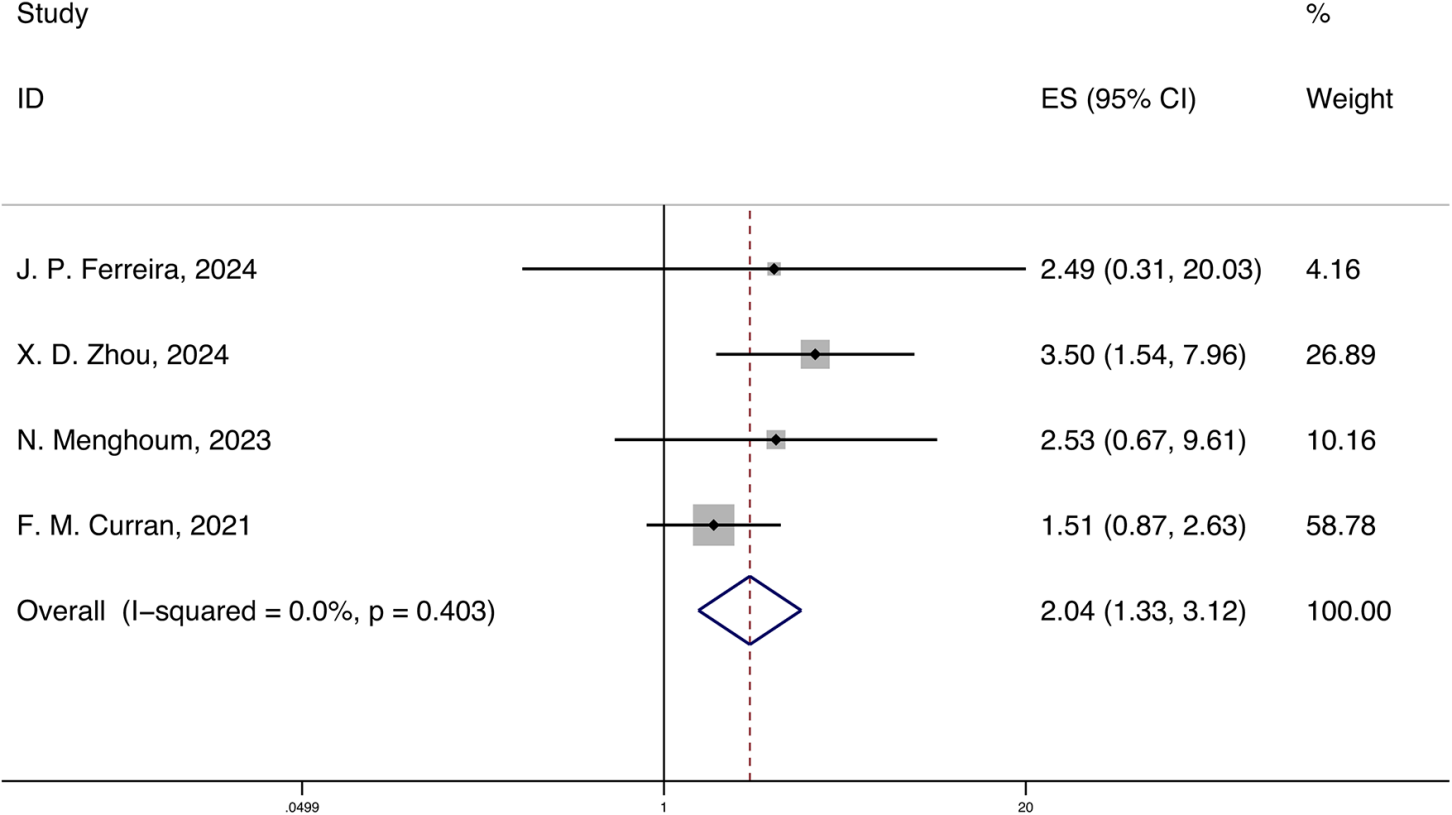
Forest plot and meta-analysis of systemic inflammatory markers and cardiovascular mortality in HFpEF.

A total of 4 studies reported the HRs and 95% CIs for the association of systemic inflammatory markers with cardiovascular mortality in HFpEF, with follow-up periods ranging from 18 months to 3.3 years. Using a fixed-effects model for meta-analysis, the results indicated that systemic inflammatory markers are correlated with cardiovascular mortality in HFpEF [HR 2.04 (1.33, 3.12), *p* < 0.05, I^2^ = 0.00%] (Figure 3).

#### 3.2.3 Prognostic value of systemic inflammatory markers for cardiovascular rehospitalization in HFpEF

**Figure 4:**
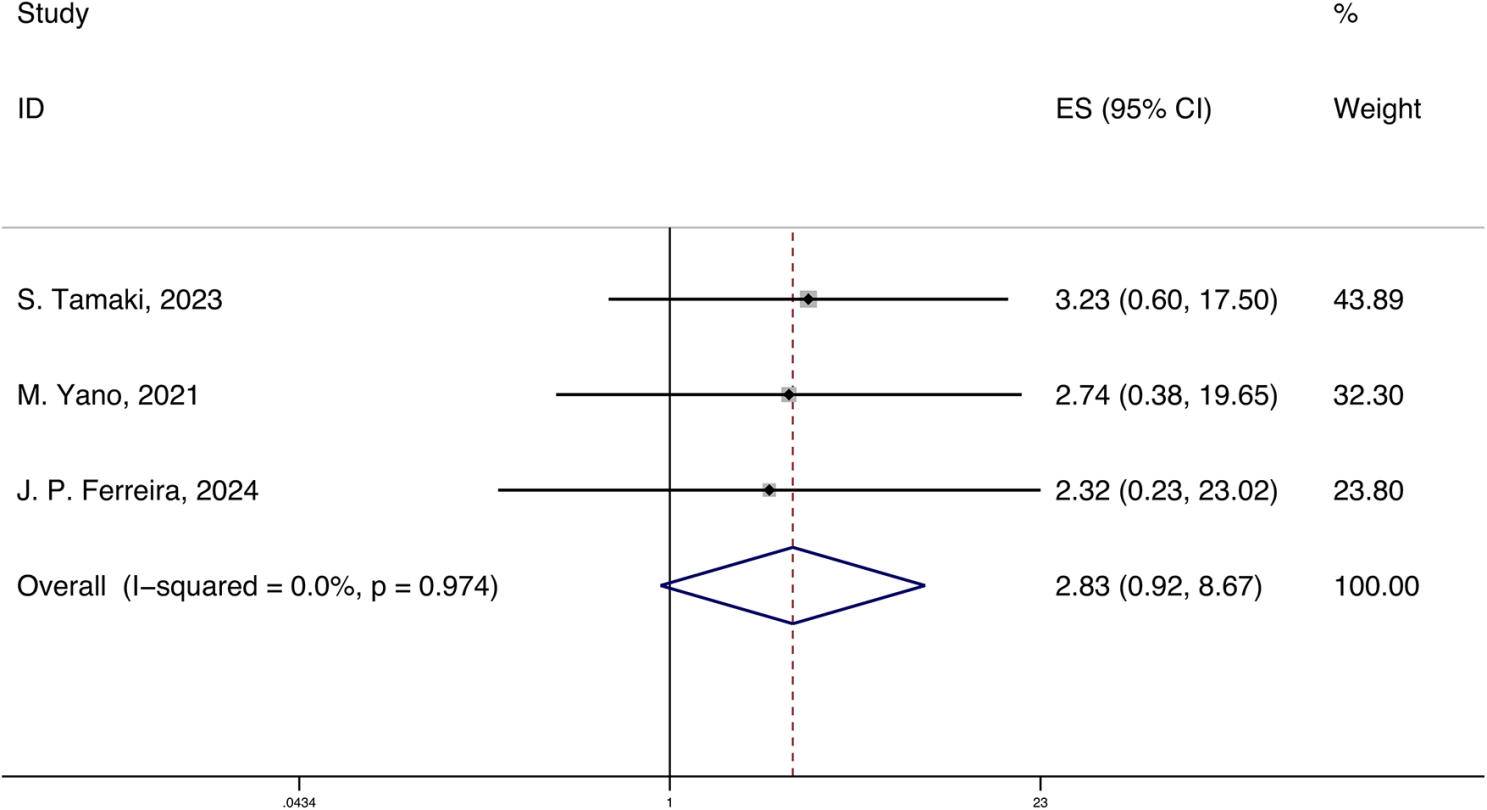
Forest plot and meta-analysis of systemic inflammatory markers and cardiovascular rehospitalization in HFpEF.

A total of 3 studies reported the HRs and 95% CIs for the association of systemic inflammatory markers with cardiovascular rehospitalization in HFpEF, with follow-up periods ranging from 420 days to 3.3 years. Using a fixed-effects model for meta-analysis, the results indicated that systemic inflammatory markers are correlated with cardiovascular rehospitalization in HFpEF [HR 2.83 (0.92, 8.67), *p* < 0.05, I^2^ = 0.00%] (Figure 4).

### 3.3 Publication bias and sensitivity analysis

In the contour-enhanced funnel plot, we can observe that the seven studies reporting all-cause mortality are essentially symmetrical along the central line and mostly fall within the central white no-effect zone. Since there were only seven studies in the contour-enhanced funnel plot, we cannot rule out the possibility of publication bias. During the trim and fill process, the direction of the HR did not change upon iterative observation.

**Figure 5:**
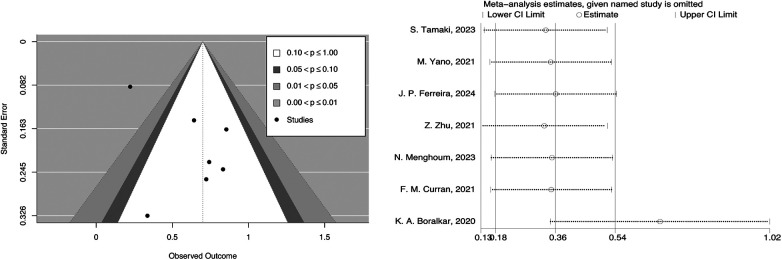
Contour-enhanced funnel plot and leave-one-out analysis of systemic inflammatory markers and all-cause mortality in HFpEF.

To assess the robustness and reliability of the results, we conducted a sensitivity analysis by leave-one-out analysis. The results indicated that the robustness might be influenced by the study of K. A. Boralkar, 2020, but fortunately, excluding this study did not affect the overall direction (Figure 5). By switching from a fixed-effects model to a random-effects model, we found that the differences in the results obtained by the two models were minimal (Supplementary Figure 1).

## 4 Discussion

### 4.1 Summary of research findings

To systematically review the predictive role of inflammatory markers for adverse clinical outcomes in HFpEF, we conducted a strict inclusion and exclusion criteria, retrieving eight prospective cohort studies from six countries (including 9,744 participants). To our knowledge, this is the first meta-analysis to explore the potential of systemic inflammatory markers in predicting the prognosis of HFpEF patients. The results show that the upregulate of systemic inflammatory markers (including NLR, PLR, hs-CRP, WBC, platelets) increases the all-cause mortality of HFpEF by 43%, cardiovascular mortality by 104%, and cardiovascular rehospitalization rate by 183%. All studies showed very low heterogeneity (I^2^ = 0). To verify the robustness of the results, we conducted a contour-enhanced funnel plot and trim and fill method, and no publication bias was found. Sensitivity analysis showed that the reliability of this study may be affected by the study of K. A. Boralkar, 2020, et al., but it did not affect the overall effect.

### 4.2 The intrinsic link between systemic inflammation and HFpEF

As research into HFpEF progresses, it has been found that systemic low-grade inflammation mediated by metabolic disorders triggers the onset of HFpEF (4). Packer and colleagues discovered a metabolic inflammatory phenotype in HFpEF, characterized by elevated circulating inflammatory biomarkers, adipose tissue dysfunction, microvascular endothelial dysfunction, insulin resistance, and infiltration of adipocytes and lipotoxicity (39). Paulus and colleagues depicted a new paradigm of systemic inflammation in the pathological links of HFpEF, where initiating factors such as overweight/obesity, insulin resistance, and salt-sensitive hypertension led to systemic persistent inflammation. Circulating inflammatory cytokines and local cardiac inflammatory factors induce coronary microvascular endothelial inflammation, which reduces the bioavailability of nitric oxide in cardiomyocytes, cyclic guanosine monophosphate levels, and protein kinase G activity, leading to increased myocardial resting tension, myocardial interstitial fibrosis, and ultimately impaired diastolic function and filling, resulting in HFpEF (40). This chronic low-grade systemic inflammation under metabolic interference is referred to as “metainflammation” (41).

### 4.3 Clinical implications

Previous articles have confirmed the effect of inflammation in heart failure, which can damage the myocardium, affect ventricular function, and promote ventricular remodeling, thereby aggravating heart failure (40). Vascular endothelial injury can result in neutrophil migration, while myocardial infiltration, which leads to increased monocyte/macrophage recruitment, is associated with aggravated fibrosis. Consequently, this study initiates with serum inflammation markers to evaluate the inflammatory status of HFpEF patients. After analyzing the correlation between systemic inflammatory marker and adverse prognosis in HFpEF patients, we concluded that inflammation increases the all-cause mortality, cardiovascular mortality, and cardiovascular readmission rate in HFpEF patients. The primary clinical implications are as follows: Firstly, it is feasible to evaluate the systemic inflammatory condition of HFpEF patients using easily accessible indicators, and it has been demonstrated that the inflammatory state is correlated with adverse outcomes. Secondly, when assessing the prognosis of HFpEF patients, attention should be given to the control of comorbidities such as metabolic diseases and infections to prevent further damage to cardiac function by systemic inflammation. Lastly, the implementation of strict and personalized inflammatory control in clinical practice may contribute to improving the prognosis of HFpEF patients, although this necessitates further research to guide the clinical treatment protocols for HFpEF.

### 4.4 Limitations

This study still faces inevitable inherent limitations. Firstly, based on the multiple comorbidities and close association with metabolic diseases of HFpEF, the values of inflammatory markers are influenced by various factors such as age, gender, and medication history, which obscure the true state of the values and prevent accurate contribution of systemic inflammatory markers to HFpEF. Secondly, due to the scarcity of available data, we cannot conduct meaningful subgroup analyses for different individual systemic inflammatory markers, and in-depth subgroup analyses could distinguish the contributions of different inflammatory markers to outcomes, providing more profound insights for clinical practice. Finally, the easier publication of positive results will inevitably lead to publication bias. Meanwhile, although we conducted an analysis of publication bias, the number of included studies was too small to rule out the existence of publication bias. In summary, although the current meta-analysis results point to the potential predictive value of systemic inflammatory markers for adverse outcomes in HFpEF, the results should still be interpreted with caution.

### 4.5 Prospects

Based on the existing evidence, we have identified several key areas for future research. Firstly, more cross-sectional and longitudinal studies should be conducted to determine the initial state of systemic inflammation levels and their dynamic changes as the disease progresses. Secondly, more basic experiments should be carried out to elucidate the pathways, targets, and molecular mechanisms and provide a more solid foundation for the development of immunoanti-inflammatory therapies. Lastly, although systemic inflammatory markers have been deeply studied in the prognosis of HFpEF, there is still an urgent need to identify and validate specific systemic inflammatory markers as biomarkers for early detection and monitoring of HFpEF and improve the early detection rate of HFpEF and prevent adverse clinical outcomes.

## 5 Conclusion

Our systematic review and meta-analysis have revealed and clarified the predictive value of systemic inflammatory markers for the clinical prognosis of HFpEF, such as all-cause mortality, cardiovascular mortality, and cardiovascular rehospitalization. This will provide a certain reference value for frontline clinicians to understand the complex pathophysiological process of HFpEF, judge prognosis, and adjust treatment strategies in a timely manner. However, due to the currently small sample size, larger prospective cohort results are still needed to further elucidate and confirm the predictive value of systemic inflammatory markers for adverse outcomes in HFpEF.

## Data Availability

The original contributions presented in the study are included in the article/Supplementary Material, further inquiries can be directed to the corresponding author.
