# Supplementary material for: Association of systemic inflammatory markers with clinical adverse prognosis and outcomes in HFpEF: a systematic review and meta-analysis of cohort studies
Source: Front Cardiovasc Med. 2024 Sep 30;11:1461073. doi: 10.3389/fcvm.2024.1461073 (PMC11471577; doi:10.3389/fcvm.2024.1461073)
Supplement: Supplementary file 1 [file Datasheet1.pdf]

Supplementary Material

1.1 Supplementary Table 1

Section and Topic	Item #	Checklist item	Location where item is reported
**TITLE**			
Title	1	Identify the report as a systematic review.	Title
**ABSTRACT**			
Abstract	2	See the PRISMA 2020 for Abstracts checklist.	Abstract
**INTRODUCTION**			
Rationale	3	Describe the rationale for the review in the context of existing knowledge.	introduction
Objectives	4	Provide an explicit statement of the objective(s) or question(s) the review addresses.	introduction
**METHODS**			
Eligibility criteria	5	Specify the inclusion and exclusion criteria for the review and how studies were grouped for the syntheses.	Section 2.1
Information sources	6	Specify all databases, registers, websites, organisations, reference lists and other sources searched or consulted to identify studies. Specify the date when each source was last searched or consulted.	Section 2.1
Search strategy	7	Present the full search strategies for all databases, registers and websites, including any filters and limits used.	Section 2.1
Selection process	8	Specify the methods used to decide whether a study met the inclusion criteria of the review, including how many reviewers screened each record and each report retrieved, whether they worked independently, and if applicable, details of automation tools used in the process.	Section 2.2
Data collection process	9	Specify the methods used to collect data from reports, including how many reviewers collected data from each report, whether they worked independently, any processes for obtaining or confirming data from study investigators, and if applicable, details of automation tools used in the process.	Section 2.2
Data items	10a	List and define all outcomes for which data were sought. Specify whether all results that were compatible with each outcome domain in each study were sought (e.g. for all measures, time points, analyses), and if not, the methods used to decide which results to collect.	Section 2.2
	10b	List and define all other variables for which data were sought (e.g. participant and intervention characteristics, funding sources). Describe any assumptions made about any missing or unclear information.	Section 2.2
Study risk of bias assessment	11	Specify the methods used to assess risk of bias in the included studies, including details of the tool(s) used, how many reviewers assessed each study and whether they worked independently, and if applicable, details of automation tools used in the process.	Section 2.3
Effect measures	12	Specify for each outcome the effect measure(s) (e.g. risk ratio, mean difference) used in the synthesis or presentation of results.	Section 2.4
Synthesis methods	13a	Describe the processes used to decide which studies were eligible for each synthesis (e.g. tabulating the study intervention characteristics and comparing against the planned groups for each synthesis (item #5)).	Section 2.4
	13b	Describe any methods required to prepare the data for presentation or synthesis, such as handling of missing summary statistics, or data conversions.	Section 2.4

Section and Topic	Item #	Checklist item	Location where item is reported
	13c	Describe any methods used to tabulate or visually display results of individual studies and syntheses.	Section 2.4
	13d	Describe any methods used to synthesize results and provide a rationale for the choice(s). If meta-analysis was performed, describe the model(s), method(s) to identify the presence and extent of statistical heterogeneity, and software package(s) used.	Section 2.4
	13e	Describe any methods used to explore possible causes of heterogeneity among study results (e.g. subgroup analysis, meta-regression).	Section 2.4
	13f	Describe any sensitivity analyses conducted to assess robustness of the synthesized results.	Section 2.4
Reporting bias assessment	14	Describe any methods used to assess risk of bias due to missing results in a synthesis (arising from reporting biases).	Section 2.4
Certainty assessment	15	Describe any methods used to assess certainty (or confidence) in the body of evidence for an outcome.	None
**RESULTS**			
Study selection	16a	Describe the results of the search and selection process, from the number of records identified in the search to the number of studies included in the review, ideally using a flow diagram.	Section 3.1
	16b	Cite studies that might appear to meet the inclusion criteria, but which were excluded, and explain why they were excluded.	Figure 1
Study characteristics	17	Cite each included study and present its characteristics.	Table 1
Risk of bias in studies	18	Present assessments of risk of bias for each included study.	Table 1
Results of individual studies	19	For all outcomes, present, for each study: (a) summary statistics for each group (where appropriate) and (b) an effect estimate and its precision (e.g. confidence/credible interval), ideally using structured tables or plots.	Section 3.1
Results of syntheses	20a	For each synthesis, briefly summarise the characteristics and risk of bias among contributing studies.	Section 3.2
	20b	Present results of all statistical syntheses conducted. If meta-analysis was done, present for each the summary estimate and its precision (e.g. confidence/credible interval) and measures of statistical heterogeneity. If comparing groups, describe the direction of the effect.	Section 3.2
	20c	Present results of all investigations of possible causes of heterogeneity among study results.	Section 3.2
	20d	Present results of all sensitivity analyses conducted to assess the robustness of the synthesized results.	Section 3.2
Reporting biases	21	Present assessments of risk of bias due to missing results (arising from reporting biases) for each synthesis assessed.	Section 3.3
Certainty of evidence	22	Present assessments of certainty (or confidence) in the body of evidence for each outcome assessed.	Section 3.2
**DISCUSSION**			
Discussion	23a	Provide a general interpretation of the results in the context of other evidence.	Section 4.1
	23b	Discuss any limitations of the evidence included in the review.	Section 4.4
	23c	Discuss any limitations of the review processes used.	Section 4.4
	23d	Discuss implications of the results for practice, policy, and future research.	Section 4.5

Section and Topic	Item #	Checklist item	Location where item is reported
**OTHER INFORMATION**			
Registration and protocol	24a	Provide registration information for the review, including register name and registration number, or state that the review was not registered.	abstract
	24b	Indicate where the review protocol can be accessed, or state that a protocol was not prepared.	abstract
	24c	Describe and explain any amendments to information provided at registration or in the protocol.	abstract
Support	25	Describe sources of financial or non-financial support for the review, and the role of the funders or sponsors in the review.	Section 8
Competing interests	26	Declare any competing interests of review authors.	Section 6
Availability of data, code and other materials	27	Report which of the following are publicly available and where they can be found: template data collection forms; data extracted from included studies; data used for all analyses; analytic code; any other materials used in the review.	None

1.2 Supplementary Table 2 Search strategy in Pubmed

Query	Results
((((((((((neutrophil-to-lymphocyte ratio[Title/Abstract]) OR (neutrophil lymphocyte ratio[Title/Abstract])) OR (NLR[Title/Abstract])) OR (lymphocyte-to-monocyte ratio[Title/Abstract])) OR (lymphocyte to monocyte ratio[Title/Abstract])) OR (LMR[Title/Abstract])) OR (platelet-to-lymphocyte ratio[Title/Abstract])) OR (platelet to lymphocyte ratio[Title/Abstract])) OR (PLR[Title/Abstract])) OR (systemic immune inflammation index[Title/Abstract])) OR (((((("Leukocytes"[Mesh]) OR "Neutrophils"[Mesh]) OR "Lymphocytes"[Mesh]) OR "Monocytes"[Mesh]) OR "Blood Platelets"[Mesh]) OR "C-Reactive Protein"[Mesh])	983,413
(((((("Heart Failure, Diastolic"[Mesh]) OR (Heart Failure, Preserved Ejection Fraction[Title/Abstract])) OR (Diastolic Heart Failure[Title/Abstract])) OR (HFpEF[Title/Abstract])) OR (Heart failure with preserved ejection fraction[Title/Abstract])) OR (diastolic function[Title/Abstract]))	21,735

((((((("Heart Failure, Diastolic"[Mesh]) OR (Heart Failure, Preserved Ejection Fraction[Title/Abstract])) OR (Diastolic Heart Failure[Title/Abstract])) OR (HFpEF[Title/Abstract])) OR (Heart failure with preserved ejection fraction[Title/Abstract])) OR (diastolic function[Title/Abstract]))) AND (((((((((((neutrophil-to-lymphocyte ratio[Title/Abstract]) OR (neutrophil lymphocyte ratio[Title/Abstract])) OR (NLR[Title/Abstract])) OR (lymphocyte-to-monocyte ratio[Title/Abstract])) OR (lymphocyte to monocyte ratio[Title/Abstract])) OR (LMR[Title/Abstract])) OR (platelet-to-lymphocyte ratio[Title/Abstract])) OR (platelet to lymphocyte ratio[Title/Abstract])) OR (PLR[Title/Abstract])) OR (systemic immune inflammation index[Title/Abstract])) OR (((((("Leukocytes"[Mesh]) OR "Neutrophils"[Mesh]) OR "Lymphocytes"[Mesh]) OR "Monocytes"[Mesh]) OR "Blood Platelets"[Mesh]) OR "C-Reactive Protein"[Mesh]))	198

1.3 Supplementary Table 3 Exclusion reasons

author	year	title	exclusion reason	journal
Tabata, T., K. Kiuchi, Y. Nagamatsu, Y. Shinkura, K. Uzu, J. I. Ooka, S. Shimoyama, T. Nishii, S. Mori, K. I. Hirata, M. Tsujimoto, S. Tajima, E. Nakano, C. Nishigori, Y. Yamamoto and S. Hara	- 2018	A case of acute heart failure due to myocardial infiltration of mycosis fungoides	case report	Journal of Cardiology Cases
Tada, Y., K. Utob, H. Wada, K. I. Sakakura, J. I. Suzuki, T. Nishikawa, J. Ako and S. I. Momomura	- 2013	A case of fulminant myocarditis with preceding repeated episodes of congestive heart failure	case report	Cardiology Research
Nakayama, S., S. Kinugasa, T. Hirose, Y. Miyake, K. Ota, M. Onzo-Toyama, I. Oba-Yabana, H. Nakamura, J. Tani, W. Yumura and T. Mori	- 2021	A case of light chain (AL) amyloidosis with heart failure, renal dysfunction, and heparin-induced thrombocytopenia successfully treated with peritoneal dialysis	case report	CEN Case Reports
Bain, E. and M. Guglin	- 2023	A case report of constrictive pericarditis following COVID-19 vaccination	case report	European Heart Journal - Case Reports
Cabanilla, M. G., E. Jones, S. V. Norville and A. Santana	- 2022	A case series of Corynebacterium striatum native valve infective endocarditis	case report	Journal of Cardiology Cases

Akasheva, D. U., E. V. Plokhova, O. N. Tkacheva, I. D. Strazhesko, A. S. Kruglikova, V. S. Pykhtina, E. N. Dudinskaya, D. A. Skvortsov, L. V. Egshatyan, N. V. Brailova, M. V. Agaltsov, I. N. Ozerova, V. A. Vygodin and S. A. Boytsov	- 2015	[Age-related Changes of Left Ventricular Diastolic Function, NT-proBNP Level and Their Association With Leukocyte Telomere Length]	irrelevent reports	Kardiologiia
Moiseeva, O. M., G. M. Aleshina, T. G. Ivanova, V. N. Kokriakov and E. V. Shliakhto	- 2006	[Cardial fibrosis and the functional activity of leukocytes in patients with essential arterial hypertension]	irrelevent reports	Vestn Ross Akad Med Nauk
Zhao, F. C., Y. L. Gao, H. S. Cai, S. W. Song, X. H. Liu and J. M. Liu	- 2021	[Effect of transcutaneous electric acupoints stimulation on vascular endothelial function and inflammatory factors after percutaneous coronary intervention]	irrelevent reports	Zhen Ci Yan Jiu
Lin, Z. P., Z. W. Zhang, R. K. Zhang, P. C. Shu and S. Q. Wu	- 2010	[Effects of rosuvastatin on left ventricular cardiac function, arteriosclerotic plaque and high sensitive C-reactive protein in hypertensive patients with mild LDL-C elevation]	irrelevent reports	Nan Fang Yi Ke Da Xue Xue Bao
Tatenkulova, S. N., V. Mareev, K. A. Zykov, N. A. Baklanova, E. A. Beliavskiĭ and N. Belenkov Iu	- 2009	[Immune mechanisms of inflammation in dilated cardiomyopathy]	irrelevent reports	Kardiologiia
Li, D. Z., Y. F. Hu and K. P. Yang	- 2006	[Protective effect of puerarin on endothelial dysfunction of heat shock protein 60 induced specific immunity in apolipoprotein E-null mice]	irrelevent reports	Zhongguo Zhong Xi Yi Jie He Za Zhi
Kratnov, A. E., E. V. Timganova and E. V. Koroleva	- 2014	[Remodeling of heart and neutrophil intracellular metabolism in obese men]	irrelevent reports	Klin Med (Mosk)
Smetanina, I. N., N. A. Vaulin, V. P. Masenko and N. A. Gratsianskiĭ	- 2006	[Short term simvastatin use in patients with heart failure of ischemic origin. Changes of blood lipids, markers of inflammation and left ventricular function]	irrelevent reports	Kardiologiia
Van Roeden, S., H. Hartog, V. Bongers, S. Thijsen and S. Sankatsing	- 2016	18F-FDG-PET Scanning Confirmed Infected Intracardiac Device-Leads with Abiotrophia defectiva	irrelevent reports	Case Reports in Cardiology
Casal Moura, M., E. S. Yi, M. J. Koster, J. H. Ryu and M. Baqir	- 2023	A 79-Year-Old Man With Recurrent Respiratory and Constitutional Symptoms, Elevated Acute Phase Reactants, and Pancytopenia	irrelevent reports	Chest
Conradi, P. M., J. F. Heidendael, C. M. H. B. Lucas, M. T. Yazdi and M. L. Handoko	- 2024	A classical case report of constrictive pericarditis, highlighting the role of magnetic resonance imaging and haemodynamic assessment	irrelevent reports	European Heart Journal - Case Reports
Bradt, N., F. Olbrechts, P. Alexander and A. Salembier	- 2022	A Dasatinib-Induced Chylothorax Persisting After the Discontinuation of Dasatinib	irrelevent reports	European Journal of Case Reports in Internal Medicine
Castagna, F., R. Kataria, S. Madan, S. Z. Ali, K. Diab, C. Leyton, A. Arfaras-Melainis, P. Kim, F. M. Giorgi, S. Vukelic, O. Saeed, S. R. Patel, D. B. Sims and U. P. Jorde	- 2021	A history of heart failure is an independent risk factor for death in patients admitted with coronavirus 19 disease	irrelevent reports	Journal of Cardiovascular Development and Disease
Liu, Z., R. Zhang, Y. Xv, J. Wang, J. Chen and X. Zhou	- 2022	A Novel Nomogram Integrated with Systemic Inflammation Markers and Traditional Prognostic Factors for Adverse Events' Prediction in Patients with Chronic Heart Failure in the Southwest of China	irrelevent reports	J Inflamm Res
Kattih, Z., A. Mahajan, M. Vojnic, J. Steinberg, A. Yurovitsky, J. A. Kim and A. Novoselac	- 2022	A Rapidly Accumulating Effusion in an Immunocompetent Woman	irrelevent reports	Chest
Yu, Q., R. R. Watson, J. J. Marchalonis and D. F. Larson	- 2005	A role for T lymphocytes in mediating cardiac diastolic function	irrelevent reports	Am J Physiol Heart Circ Physiol
Sarkar, S. and J. Biswas	- 2020	A study of association between high sensitivity C-reactive protein and diastolic dysfunction in patients with cardiac risk factors	irrelevent reports	Journal of the Indian Medical Association
Rawat, V., N. Nahar, S. Alawe and M. Meena	- 2023	A STUDY OF CLINICAL PROFILE AND PREDICTORS OF OUTCOME IN CARDIAC PATIENTS WITH PRESERVED EJECTION FRACTION REQUIRING MECHANICAL VENTILATION	irrelevent reports	Journal of Cardiovascular Disease Research
Lamonte, M. J., A. Z. Lacroix, S. Nguyen, K. R. Evenson, C. Di, M. L. Stefanick, E. T. Hyde, B. Anuskiewicz and C. B. Eaton	- 2024	Accelerometer-Measured Physical Activity, Sedentary Time, and Heart Failure Risk in Women Aged 63 to 99 Years	irrelevent reports	JAMA Cardiology

Kim, Y. H., A. Y. Her, M. H. Jeong, B. K. Kim, S. J. Hong, S. Kim, C. M. Ahn, J. S. Kim, Y. G. Ko, D. Choi, M. K. Hong and Y. Jang	- 2020	ACE Inhibitors Versus ARBs in Patients With NSTEMI With Preserved LV Systolic Function Who Underwent PCI With New Generation Drug-Eluting Stents	irrelevent reports	Angiology
Masutani, S.	- 2019	Activin a — A potentially useful biomarker of diastolic dysfunction —	irrelevent reports	Circulation Journal
Kishi, S., S. Yamada, F. Kishi, E. Shibata, M. Matsuura, K. Nagai, A. Mima, H. Abe and T. Doi	- 2012	Acute glomerulonephritis in an immunocompetent elderly woman after contact with a child who had been diagnosed as erythema infectiosum	irrelevent reports	Internal Medicine
Takami, T., H. Ito, K. Ishii, K. Shimada, K. Iwakura, H. Watanabe, S. Fukuda and J. Yoshikawa	- 2012	Adding thiazide to a rennin-angiotensin blocker regimen to improve left ventricular relaxation in diabetes and nondiabetes patients with hypertension	irrelevent reports	Drug Des Devel Ther
Berezina, T. A., Z. Obradovic, E. Boxhammer, A. A. Berezin, M. Lichtenauer and A. E. Berezin	- 2023	Adropin Predicts Chronic Kidney Disease in Type 2 Diabetes Mellitus Patients with Chronic Heart Failure	irrelevent reports	Journal of Clinical Medicine
Brown, J. M.	- 2024	Adverse Effects of Aldosterone: Beyond Blood Pressure	irrelevent reports	Journal of the American Heart Association
Akasheva, D. U., E. V. Plokhova, O. N. Tkacheva, I. D. Strazhesko, E. N. Dudinskaya, A. S. Kruglikova, V. S. Pykhtina, N. V. Brailova, I. A. Pokshubina, N. V. Sharashkina, M. V. Agaltsov, D. Skvortsov and S. A. Boytsov	- 2015	Age-Related Left Ventricular Changes and Their Association with Leukocyte Telomere Length in Healthy People	irrelevent reports	PLoS One
Nguyen, Q. L., Y. Wang, N. Helbling, M. A. Simon and S. Shiva	- 2019	Alterations in platelet bioenergetics in Group 2 PH-HFpEF patients	irrelevent reports	PLoS One
Lu, J., F. Liu, F. Chen, Y. Jin, H. Chen, D. Liu and W. Cui	- 2016	Amlodipine and atorvastatin improve ventricular hypertrophy and diastolic function via inhibiting TNF-α, IL-1β and NF-κB inflammatory cytokine networks in elderly spontaneously hypertensive rats	irrelevent reports	Biomed Pharmacother
Koren, R., O. Tzuman and R. Zaidenstein	- 2014	Amyloid heart: heart failure with preserved ejection fraction--a rare cause of a common illness	irrelevent reports	Isr Med Assoc J
Nojima, Y., M. Ihara, T. Kurimoto and S. Nanto	- 2016	Amyloid light-chain amyloidosis manifesting as heart failure with preserved ejection fraction in a patient with hyper-immunoglobulin E-emia	irrelevent reports	American Journal of Case Reports
Keogh, A., F. Lynott, A. Papanicolau-Sengos, M. Mohammed Nur, A. Spillane, F. Quinn, E. ElHassadi, E. S. Jaffe and R. Flavin	- 2022	An Isolated Mesenteric Presentation of a Nodal Peripheral T Cell Lymphoma with T Follicular Helper Cell Phenotype	irrelevent reports	Hematology Reports
Hung, C. L., C. H. Yun, Y. H. Lai, K. T. Sung, H. G. Bezerra, J. Y. Kuo, C. J. Y. Hou, T. F. Chao, B. E. Bulwer, H. I. Yeh, S. C. Shih, S. J. Lin and R. C. Cury	- 2016	An observational study of the association among interatrial adiposity by computed tomography measure, insulin resistance, and left atrial electromechanical disturbances in heart failure	irrelevent reports	Medicine (United States)
Schattner, A., I. Dubin, Y. Glick and S. Haimovich	- 2023	An Unusual Cause of Prolonged Fever in an Elderly Woman – Pyometra	irrelevent reports	American Journal of Medicine
Yang, Y., X. Li, Y. Gu, Z. Lyu, Y. Liang, M. Jiao and M. Jin	- 2024	Analysis of clinical features of heart failure in children with cardiomyopathy and improved ejection fraction	irrelevent reports	Translational Pediatrics
Charles, E., B. L. Dumont, S. Bonneau, P. E. Neagoe, L. Villeneuve, A. Räkel, M. White and M. G. Sirois	- 2021	Angiopoietin 1 release from human neutrophils is independent from neutrophil extracellular traps (NETs)	irrelevent reports	BMC Immunol
Hu, Y., C. Zhang, C. Zou, H. Yang, Y. Chen and T. Liang	- 2023	Anthropometric measures and physical examination could be used to assess phenotypic GLIM (Global leadership initiative on malnutrition) criteria in heart failure patients	irrelevent reports	Nutrition, Metabolism and Cardiovascular Diseases

Pinchuk, T. V., Y. N. Fedulaev, G. A. Khairetdinova, N. N. Denisova, O. V. Chura and I. Y. Logunova	- 2014	Anti-Inflammatory Effects of Simvastatin in Patients with Chronic Heart Failure	irrelevent reports	Bulletin of Experimental Biology and Medicine
Rethy, L., B. A. Borlaug, M. M. Redfield, J. K. Oh, S. J. Shah and R. B. Patel	- 2021	Application of Guideline-Based Echocardiographic Assessment of Left Atrial Pressure to Heart Failure with Preserved Ejection Fraction	irrelevent reports	Journal of the American Society of Echocardiography
Mertens, J. and M. Haddad	- 2022	Artifactual hypoglycemia in a patient with systemic sclerosis	irrelevent reports	Acta Clinica Belgica: International Journal of Clinical and Laboratory Medicine
Liu, B. H., Y. G. Li, J. X. Liu, X. J. Zhao, Q. Jia, C. L. Liu, Z. G. Xu and K. L. He	- 2019	Assessing inflammation in Chinese subjects with subtypes of heart failure: An observational study of the Chinese PLA Hospital Heart Failure Registry	irrelevent reports	Journal of Geriatric Cardiology
Yagmur, J., M. Cansel, N. Acikgoz, N. Ermis, M. Yagmur, H. Atas, H. Tasolar, Y. Karakus, H. Pekdemir and R. Ozdemir	- 2011	Assessment of atrial electromechanical delay by tissue Doppler echocardiography in obese subjects	irrelevent reports	Obesity (Silver Spring)
Misztal, M., K. Stopyra, A. Gackowski, K. Zmudka and W. Piwowarska	- 2009	Assessment of left ventricle diastolic function in myocardial infarction patients treated with primary angioplasty	irrelevent reports	Cardiol J
Inci, U., A. Yildiz, I. Batmaz and E. Tekbas	- 2017	Assessment of serum asymmetric dimethylarginine levels and left ventricular diastolic function in patients with ankylosing spondylitis	irrelevent reports	Int J Rheum Dis
Peng, Z., Q. Zhan, X. Xie, H. Li, Y. Tu, Y. Bai, X. Huang, W. Lai, B. Zhao, Q. Zeng and D. Xu	- 2019	Association between admission plasma 2-oxoglutarate levels and short-term outcomes in patients with acute heart failure: A prospective cohort study	irrelevent reports	Molecular Medicine
Shacham, Y., Y. Topilsky, E. Leshem-Rubinow, Y. Arbel, E. Ben Assa, G. Keren, A. Roth and A. Steinvil	- 2014	Association between C-reactive protein level and echocardiography assessed left ventricular function in first ST-segment elevation myocardial infarction patients who underwent primary coronary intervention	irrelevent reports	J Cardiol
Oreopoulos, A., J. A. Ezekowitz, F. A. McAlister, K. Kalantar-Zadeh, G. C. Fonarow, C. M. Norris, J. A. Johnson and R. S. Padwal	- 2010	Association between direct measures of body composition and prognostic factors in chronic heart failure	irrelevent reports	Mayo Clin Proc
Abohamr, S. I., R. M. Abazid, M. K. Alhumaid, A. E. Abdulrahim, M. A. Aldossari, L. Khedr, R. H. Werida, H. S. Alkheledan, Y. S. Aleid, S. W. Abdelhamid, A. Al Mefarrej, A. W. Abdelhamid, M. H. Alaboud, O. T. Alhasan, H. M. Gomaa and E. Elsheikh	- 2023	Association between echocardiographic features and inflammatory biomarkers with clinical outcomes in COVID-19 patients in Saudi Arabia	irrelevent reports	Frontiers in Cardiovascular Medicine
Barasch, E., J. S. Gottdiener, G. Aurigemma, D. W. Kitzman, J. Han, W. J. Kop and R. P. Tracy	- 2009	Association between elevated fibrosis markers and heart failure in the elderly the cardiovascular health study	irrelevent reports	Circulation: Heart Failure
Takei, Y., H. Tomiyama, Y. Higashi, A. Yamashina and T. Chikamori	- 2023	Association Between Endothelial Dysfunction and Left Ventricular Diastolic Stiffness ― Subanalysis of the Flow-Mediated Dilation Japan (FMD-J) Study ―	irrelevent reports	Circulation Journal
Li, N., M. Zhao, L. Yuan, Y. Chen and H. Zhou	- 2023	Association between glycosylated hemoglobin levels, diabetes duration, and left ventricular diastolic dysfunction in patients with type 2 diabetes and preserved ejection fraction: a cross-sectional study	irrelevent reports	Frontiers in Endocrinology
Masugata, H., S. Senda, M. Inukai, K. Murao, S. Tada, N. Hosomi, Y. Iwado, T. Noma, M. Kohno, T. Himoto and F. Goda	- 2011	Association between high-sensitivity C-reactive protein and left ventricular diastolic function assessed by echocardiography in patients with cardiovascular risk factors	irrelevent reports	Tohoku J Exp Med
Huang, S., T. Cai, B. N. Weber, Z. He, K. P. Dahal, C. Hong, J. Hou, T. Seyok, A. Cagan, M. F. DiCarli, J. Joseph, S. C. Kim, D. H. Solomon, T. Cai and K. P. Liao	- 2023	Association Between Inflammation, Incident Heart Failure, and Heart Failure Subtypes in Patients With Rheumatoid Arthritis	irrelevent reports	Arthritis Care and Research
Kim, G., T. Y. Yu, J. H. Jee, J. C. Bae, M. Kang and J. H. Kim	- 2024	Association between nonalcoholic fatty liver disease and left ventricular diastolic dysfunction: A 7-year retrospective cohort study of 3,380 adults using serial echocardiography	irrelevent reports	Diabetes and Metabolism

Samman Tahhan, A., P. B. Sandesara, S. S. Hayek, A. Alkhoder, K. Chivukula, M. Hammadah, H. Mohamed-Kelli, W. T. O'Neal, M. Topel, N. Ghasemzadeh, Y. A. Ko, H. Aida, M. Gafeer, L. Sperling, V. Vaccarino, Y. Liang, D. P. Jones and A. A. Quyyumi	- 2017	Association between oxidative stress and atrial fibrillation	irrelevent reports	Heart Rhythm
Meng, Y., T. Zhao, Z. Y. Zhang and D. K. Zhang	- 2020	Association between sub-clinical hypothyroidism and heart failure with preserved ejection fraction	irrelevent reports	Chinese Medical Journal
De Boer, R. A., M. Nayor, C. R. DeFilippi, D. Enserro, V. Bhambhani, J. R. Kizer, M. J. Blaha, F. P. Brouwers, M. Cushman, J. A. C. Lima, H. Bahrami, P. Van Der Harst, T. J. Wang, R. T. Gansevoort, C. S. Fox, H. K. Gaggin, W. J. Kop, K. Liu, R. S. Vasan, B. M. Psaty, D. S. Lee, H. L. Hillege, T. M. Bartz, E. J. Benjamin, C. Chan, M. Allison, J. M. Gardin, J. L. Januzzi, S. J. Shah, D. Levy, D. M. Herrington, M. G. Larson, W. H. Van Gilst, J. S. Gottdiener, A. G. Bertoni and J. E. Ho	- 2018	Association of cardiovascular biomarkers with incident heart failure with preserved and reduced ejection fraction	irrelevent reports	JAMA Cardiology
Tun, B., R. Ehrbar, M. Short, S. Cheng, R. S. Vasan and V. Xanthakis	- 2020	Association of exhaled carbon monoxide with ideal cardiovascular health, circulating biomarkers, and incidence of heart failure in the framingham offspring study	irrelevent reports	Journal of the American Heart Association
Tsui, J. I., M. A. Whooley, A. Monto, K. Seal, P. C. Tien and M. Shlipak	- 2009	Association of hepatitis C virus seropositivity with inflammatory markers and heart failure in persons with coronary heart disease: data from the Heart and Soul study	irrelevent reports	J Card Fail
Sinha, A., C. M. Sitlani, M. F. Doyle, A. E. Fohner, P. Buzkova, J. S. Floyd, S. A. Huber, N. C. Olson, J. N. Njoroge, J. R. Kizer, J. A. Delaney, S. S. Shah, R. P. Tracy, B. Psaty and M. Feinstein	- 2022	Association of immune cell subsets with incident heart failure in two population-based cohorts	irrelevent reports	ESC Heart Failure
Jia, X., L. Buckley, C. Sun, M. Al Rifai, B. Yu, V. Nambi, S. S. Virani, E. Selvin, K. Matsushita, R. C. Hoogeveen, J. Coresh, A. M. Shah and C. M. Ballantyne	- 2023	Association of interleukin-6 and interleukin-18 with cardiovascular disease in older adults: Atherosclerosis Risk in Communities study	irrelevent reports	European Journal of Preventive Cardiology
Hsu, H. C., G. R. Norton, F. Peters, C. Robinson, N. Dlongolo, A. Solomon, G. Teckie, A. J. Woodiwiss and P. H. Dessein	- 2021	Association of post transplantation anaemia and persistent secondary hyperparathyroidism with diastolic function in stable kidney transplant recipients	irrelevent reports	International Journal of Nephrology and Renovascular Disease
Ramalho, S. H. R., B. L. Claggett, G. R. Washko, R. S. J. Estepar, P. P. Chang, D. W. Kitzman, G. C. Junior, S. D. Solomon, H. Skali and A. M. Shah	- 2022	Association of Pulmonary Function With Late-Life Cardiac Function and Heart Failure Risk: The ARIC Study	irrelevent reports	Journal of the American Heart Association
Liu, C., Y. Lai, T. Guan, J. Zhan, J. Pei, D. Wu, S. Ying and Y. Shen	- 2022	Associations of ATP-Sensitive Potassium Channel’s Gene Polymorphisms With Type 2 Diabetes and Related Cardiovascular Phenotypes	irrelevent reports	Frontiers in Cardiovascular Medicine
Mokotedi, L., F. S. Michel, C. Mogane, M. Gomes, A. J. Woodiwiss, G. R. Norton and A. M. E. Millen	- 2020	Associations of inflammatory markers with impaired left ventricular diastolic and systolic function in collagen-induced arthritis	irrelevent reports	PLoS One
Liu, L., J. A. C. Lima, W. S. Post and M. Szklo	- 2021	Associations of time-varying obesity and metabolic syndrome with risk of incident heart failure and its subtypes: Findings from the Multi-Ethnic Study of Atherosclerosis	irrelevent reports	International Journal of Cardiology

Yerebakan, C., E. Sandica, S. Prietz, C. Klopsch, M. Ugurlucan, A. Kaminski, S. Abdija, B. Lorenzen, J. Boltze, B. Nitzsche, D. Egger, M. Barten, D. Furlani, N. Ma, B. Vollmar, A. Liebold and G. Steinhoff	- 2009	Autologous umbilical cord blood mononuclear cell transplantation preserves right ventricular function in a novel model of chronic right ventricular volume overload	irrelevent reports	Cell Transplant
Şentürk, B., A. Çelik, L. Bekar, U. Uyan, S. Y. Tülüce, H. Güneş, M. Kerkütlüoğlu, A. Çoner, B. Kılıçaslan, N. Çetin, A. Çolak, B. Açar, İ. Gül, C. Altın, Y. Özkurt, M. K. Karadağ, E. Kalaycıoğlu, M. Özkahya, T. Ekin, N. Yılmaz and M. B. Yılmaz	- 2022	Baseline characteristics of outpatients with heart failure according to phenotype: preliminary analysis from SMYRNA-HF registry	irrelevent reports	European Research Journal
Suthahar, N., C. Tschöpe and R. A. De Boer	- 2021	Being in Two Minds - The Challenge of Heart Failure with Preserved Ejection Fraction Diagnosis with a Single Biomarker	irrelevent reports	Clinical Chemistry
Rider, O. J., J. M. Francis, M. K. Ali, S. E. Petersen, M. Robinson, M. D. Robson, J. P. Byrne, K. Clarke and S. Neubauer	- 2009	Beneficial cardiovascular effects of bariatric surgical and dietary weight loss in obesity	irrelevent reports	J Am Coll Cardiol
Patel, R. B., F. Alenezi, J. L. Sun, B. Alhanti, M. Vaduganathan, J. K. Oh, M. M. Redfield, J. Butler, A. F. Hernandez, E. J. Velazquez and S. J. Shah	- 2020	Biomarker Profile of Left Atrial Myopathy in Heart Failure With Preserved Ejection Fraction: Insights From the RELAX Trial: Biomarkers in Left Atrial Myopathy	irrelevent reports	Journal of Cardiac Failure
Tromp, J., M. A. F. Khan, I. T. Klip, S. Meyer, R. A. de Boer, T. Jaarsma, H. Hillege, D. J. van Veldhuisen, P. van der Meer and A. A. Voors	- 2017	Biomarker profiles in heart failure patients with preserved and reduced ejection fraction	irrelevent reports	Journal of the American Heart Association
Tromp, J., M. A. F. Khan, R. J. Mentz, C. M. O'Connor, M. Metra, H. C. Dittrich, P. Ponikowski, J. R. Teerlink, G. Cotter, B. Davison, J. G. F. Cleland, M. M. Givertz, D. M. Bloomfield, D. J. Van Veldhuisen, H. L. Hillege, A. A. Voors and P. van der Meer	- 2017	Biomarker Profiles of Acute Heart Failure Patients With a Mid-Range Ejection Fraction	irrelevent reports	JACC: Heart Failure
Aulin, J., Z. Hijazi, J. Lindbäck, J. H. Alexander, B. J. Gersh, C. B. Granger, M. Hanna, J. Horowitz, R. D. Lopes, J. J. V. McMurray, J. Oldgren, A. Siegbahn and L. Wallentin	- 2022	Biomarkers and heart failure events in patients with atrial fibrillation in the ARISTOTLE trial evaluated by a multi-state model	irrelevent reports	American Heart Journal
Sinning, C., T. Kempf, M. Schwarzl, S. Lanfermann, F. Ojeda, R. B. Schnabel, E. Zengin, P. S. Wild, K. J. Lackner, T. Munzel, S. Blankenberg, K. C. Wollert, T. Zeller and D. Westermann	- 2017	Biomarkers for characterization of heart failure - Distinction of heart failure with preserved and reduced ejection fraction	irrelevent reports	Int J Cardiol
Merino-Merino, A., R. Saez-Maleta, R. Salgado-Aranda, D. AlKassam-Martinez, V. Pascual-Tejerina, J. Martin-Gonzalez, J. Garcia-Fernandez and J. A. Perez-Rivera	- 2020	Biomarkers in atrial fibrillation and heart failure with non-reduced ejection fraction: Diagnostic application and new cut-off points	irrelevent reports	Heart and Lung
Musikhina, N. A., T. I. Petelina, A. I. Kostousova, L. I. Gapon, E. A. Gorbatenko and I. S. Bessonov	- 2020	Biomarkers of inflammation in patients with myocardial infarction and heart failure with preserved and mid-range ejection fraction: 5-year prospective follow-up	irrelevent reports	Russian Journal of Cardiology

Anguita, E., A. Chaparro, F. J. Candel, C. Ramos-Acosta, N. Martínez-Micaelo, N. Amigó, M. J. Torrejón, G. Llopis-García, M. D. M. Suárez-Cadenas, M. Matesanz, J. González del Castillo and F. J. Martín-Sánchez	- 2022	Biomarkers of stable and decompensated phases of heart failure with preserved ejection fraction	irrelevent reports	International Journal of Cardiology
Moliner, P., J. Lupón, J. Barallat, M. de Antonio, M. Domingo, J. Núñez, E. Zamora, A. Galán, J. Santesmases, C. Pastor and A. Bayes-Genis	- 2018	Bio-profiling and bio-prognostication of chronic heart failure with mid-range ejection fraction	irrelevent reports	International Journal of Cardiology
Olagunju, A., C. Shekar, M. Morris, A. Kalya, F. Mookadam and S. Unzek	- 2022	Blood Cancer and the Heart: Light Chain Cardiomyopathy in Refractory Multiple Myeloma	irrelevent reports	Case Reports in Cardiology
Shantsila, E., N. Bialiuk, D. Navitski, A. Pyrochkin, P. S. Gill, V. Pyrochkin, V. Snezhitskiy and G. Y. Lip	- 2012	Blood leukocytes in heart failure with preserved ejection fraction: impact on prognosis	irrelevent reports	Int J Cardiol
Witczak, B. N., J. Bollerslev, K. Godang, T. Schwartz, B. Flatø, Ø. Molberg, I. Sjaastad and H. Sanner	- 2022	Body composition in long-standing juvenile dermatomyositis: associations with disease activity, muscle strength and cardiometabolic measures	irrelevent reports	Rheumatology (Oxford)
Parker, S. J., D. N. Didier, J. R. Karcher, T. J. Stodola, B. Endres and A. S. Greene	- 2012	Bone marrow mononuclear cells induce beneficial remodeling and reduce diastolic dysfunction in the left ventricle of hypertensive SS/MCWi rats	irrelevent reports	Physiol Genomics
Lovell, M. J., M. Yasin, K. L. Lee, K. K. Cheung, Y. Shintani, M. Collino, A. Sivarajah, K. Y. Leung, K. Takahashi, A. Kapoor, M. M. Yaqoob, K. Suzuki, M. F. Lythgoe, J. Martin, P. B. Munroe, C. Thiemermann and A. Mathur	- 2010	Bone marrow mononuclear cells reduce myocardial reperfusion injury by activating the PI3K/Akt survival pathway	irrelevent reports	Atherosclerosis
Chrysohoou, C., G. Georgiopoulos, H. Kosyfa, I. Kotsopoulou Haritou, M. Kouvari, A. Filippou, S. Iosifidis, E. Tsiamis, P. Aggelopoulos, C. Pitsavos and D. Tousoulis	- 2018	Brain Natriuretic Peptide mediates the prognostic role of renal function toward 10-year cardiovascular mortality in patients with Acute Coronary Syndrome: the HHF study (2006–2016)	irrelevent reports	Hellenic Journal of Cardiology
Harada, E., Y. Mizuno, F. Kugimiya, M. Shono, H. Maeda, N. Yano, K. Kuwahara and H. Yasue	- 2017	B-type natriuretic peptide in heart failure with preserved ejection fraction: Relevance to age-related left ventricular modeling in Japanese	irrelevent reports	Circulation Journal
Bartko, P. E., G. Heitzinger, N. Pavo, M. Heitzinger, G. Spinka, S. Prausmüller, H. Arfsten, M. Andreas, C. Gabler, G. Strunk, J. Mascherbauer, C. Hengstenberg, M. Hülsmann and G. Goliasch	- 2021	Burden, treatment use, and outcome of secondary mitral regurgitation across the spectrum of heart failure: observational cohort study	irrelevent reports	The BMJ
Menghoum, N., M. C. Badii, M. Deltombe, S. Lejeune, C. Roy, D. Vancraeynest, A. Pasquet, B. L. Gerber, S. Horman, D. Gruson, C. Beauloye and A. C. Pouleur	- 2024	Carbohydrate antigen 125: a useful marker of congestion, fibrosis, and prognosis in heart failure with preserved ejection fraction	irrelevent reports	ESC Heart Failure
Lee, S. K., M. J. Song, S. H. Kim and H. J. Ahn	- 2018	Cardiac diastolic dysfunction predicts poor prognosis in patients with decompensated liver cirrhosis	irrelevent reports	Clinical and Molecular Hepatology
Dogan, A., O. Dogdu, I. Ozdogru, M. Yarlioglues, N. Kalay, M. T. Inanc, I. Ardic, A. Celik, L. Kaynar, F. Kurnaz, N. K. Eryol and M. G. Kaya	- 2013	Cardiac effects of chronic graft-versus-host disease after stem cell transplantation	irrelevent reports	Tex Heart Inst J

Mutlu, D., U. Raimoğlu, M. Cimci, S. N. Ömeroğlu, E. Durmaz, B. İkitimur and B. Karadağ	- 2022	Cardiac Hydatid Disease and Peritoneal Tuberculosis Coexistence	irrelevent reports	Turk Kardiyoloji Dernegi Arsivi
Hulsmans, M., H. B. Sager, J. D. Roh, M. Valero-Muñoz, N. E. Houstis, Y. Iwamoto, Y. Sun, R. M. Wilson, G. Wojtkiewicz, B. Tricot, M. T. Osborne, J. Hung, C. Vinegoni, K. Naxerova, D. E. Sosnovik, M. R. Zile, A. D. Bradshaw, R. Liao, A. Tawakol, R. Weissleder, A. Rosenzweig, F. K. Swirski, F. Sam and M. Nahrendorf	- 2018	Cardiac macrophages promote diastolic dysfunction	irrelevent reports	J Exp Med
Corbi, G., V. Conti, J. Troisi, A. Colucci, V. Manzo, P. Di Pietro, M. C. Calabrese, A. Carrizzo, C. Vecchione, N. Ferrara and A. Filippelli	- 2019	Cardiac rehabilitation increases SIRT1 activity and β -Hydroxybutyrate levels and decreases oxidative stress in patients with HF with preserved ejection fraction	irrelevent reports	Oxidative Medicine and Cellular Longevity
Santorio, N. C., F. A. M. Cardozo, R. F. Miada, F. G. Pitta, C. De Assis Moura Tavares, F. C. Habrum, H. T. Pinesi, I. R. Magalhães, M. C. S. Menezes, B. Caramelli and D. Calderaro	- 2021	Cardiology referral during the COVID-19 pandemic	irrelevent reports	Clinics
Balletti, A., N. De Biase, L. Del Punta, F. Filidei, S. Armenia, F. Masi, V. Di Fiore, M. Mazzola, A. Bacca, F. L. Dini, S. Taddei, S. Masi and N. R. Pugliese	- 2023	Cardiometabolic Phenotyping in Heart Failure: Differences between Patients with Reduced vs. Preserved Ejection Fraction	irrelevent reports	Diagnostics
Lindman, B. R., V. G. Dávila-Román, D. L. Mann, S. McNulty, M. J. Semigran, G. D. Lewis, L. De Las Fuentes, S. M. Joseph, J. Vader, A. F. Hernandez and M. M. Redfield	- 2014	Cardiovascular phenotype in HFpEF patients with or without diabetes: A RELAX trial ancillary study	irrelevent reports	Journal of the American College of Cardiology
Oliveira, A. N., M. M. Simões, R. Simões, M. V. B. Malachias and B. A. Rezende	- 2019	Cardiovascular Risk in Psoriasis Patients: Clinical, Functional and Morphological Parameters	irrelevent reports	Arq Bras Cardiol
Kojima, M., K. Sato, G. Kimura, R. Ueda and Y. Dohi	- 2007	Carvedilol reduces elevated B-type natriuretic peptide in dialyzed patients without heart failure: cardioprotective effect of the beta-blocker	irrelevent reports	J Cardiovasc Pharmacol
Amelotti, N., M. Brusamolino, M. Mapelli, M. Contini, A. Baggiano, F. Fazzari, G. Pontone and P. Agostoni	- 2024	Case report: acute myocarditis in two patients with coronary artery disease presenting with chest pain—thinking outside the box	irrelevent reports	European Heart Journal - Case Reports
Wang, Y., M. Qian, X. Jin, J. Wang, T. Chen, P. Gao, Z. Cheng, J. Lai, Y. Liu, J. Fan, L. Zhang, K. Cheng, H. Deng, Q. Fang and D. Yang	- 2024	Case Report: Temporary pacing using active fixation lead and invasive electrophysiology studies for immune checkpoint inhibitor associated reversible advanced atrioventricular block	irrelevent reports	Frontiers in Cardiovascular Medicine
Okeke, R. K., G. A. Harmon, I. G. Okeke, J. W. Schuler, S. J. Sangappa, J. S. Harmon, E. Angelova, X. Sun and A. A. Chinnici	- 2023	Catch the Calcium: T-Cell Histiocyte-Rich B-Cell Lymphoma Presenting as Hypercalcemia	irrelevent reports	World Journal of Oncology
Mavrea, A. M., T. Dragomir, D. A. Bordejevic, M. C. Tomescu, O. Ancusa and I. Marincu	- 2015	Causes and predictors of hospital readmissions in patients older than 65 years hospitalized for heart failure with preserved left ventricular ejection fraction in western Romania	irrelevent reports	Clinical Interventions in Aging
Suppamutharwyam, M. and T. M. Shah	- 2023	Cefepime-induced delirium	irrelevent reports	Progress in Neurology and Psychiatry
Golino, M., F. Moroni, M. G. Del Buono, J. M. Canada, A. H. Talasaz, S. Piñel, J. Mbualungu, A. Vecchiè, A. C. Ho, G. K. Thomas, S. Carbone, H. E. Billingsley, J. Turlington, R. Markley, C. Trankle, R. De Ponti, B. Van Tassell and A. Abbate	- 2023	Change in Eosinophil Count in Patients with Heart Failure Treated with Anakinra	irrelevent reports	Cells

Vitiello, D., F. Harel, R. M. Touyz, M. G. Sirois, J. Lavoie, J. Myers, A. Ducharme, N. Racine, E. O'Meara, M. Gayda, M. Chabot-Blanchet, J. L. Rouleau, S. De Denus and M. White	- 2014	Changes in cardiopulmonary reserve and peripheral arterial function concomitantly with subclinical inflammation and oxidative stress in patients with heart failure with preserved ejection fraction	irrelevent reports	International Journal of Vascular Medicine
Hungerford, S., K. Kearney, N. Song, E. Kotlyar, N. K. Bart, E. Lau, A. Jabbour, C. S. Hayward, D. W. M. Muller and A. Adji	- 2023	Characteristic changes to pulsatile and steady-state load according to pulmonary hypertension classification	irrelevent reports	Physiological Reports
Jalali, F., F. Hatami, M. Saravi, I. Jafaripour, M. T. Hedayati, K. Amin, R. Pourkia, S. Abroutan, M. Javanian, S. Ebrahimpour, N. Valizadeh, S. K. Bizhaem and N. Ziaie	- 2021	Characteristics and outcomes of hospitalized patients with cardiovascular complications of COVID-19	irrelevent reports	Journal of Cardiovascular and Thoracic Research
Reddy, Y. N. V., G. D. Lewis, S. J. Shah, M. Obokata, O. F. Abou-Ezzedine, M. Fudim, J. L. Sun, H. Chakraborty, S. McNulty, M. M. LeWinter, D. L. Mann, L. W. Stevenson, M. M. Redfield and B. A. Borlaug	- 2019	Characterization of the Obese Phenotype of Heart Failure With Preserved Ejection Fraction: A RELAX Trial Ancillary Study	irrelevent reports	Mayo Clinic Proceedings
Shin, W. J., H. M. Kwon, S. H. Kim, H. Y. Jang, J. Y. Kim, J. H. Kim, K. S. Kim, Y. J. Moon, I. G. Jun, J. G. Song and G. S. Hwang	- 2023	Characterizing Heart Failure With Preserved Ejection Fraction in End-Stage Liver Disease and Liver Transplant Outcomes	irrelevent reports	JACC: Asia
Kirillova, I. G., D. S. Novikova, T. V. Popkova, H. V. Udachkina, E. I. Markelova, Y. N. Gorbunova, Y. O. Korsakova and S. N. Gluchova	- 2020	Chronic heart failure in early rheumatoid arthritis patients prior to basic antirheumatic therapy	irrelevent reports	Rational Pharmacotherapy in Cardiology
Wang, Z., L. Yu, S. Wang, B. Huang, K. Liao, G. Saren, T. Tan and H. Jiang	- 2014	Chronic intermittent low-level transcutaneous electrical stimulation of auricular branch of vagus nerve improves left ventricular remodeling in conscious dogs with healed myocardial infarction	irrelevent reports	Circ Heart Fail
He, J., M. Pan, M. Xu and R. Chen	- 2021	Circulating miRNA-29b and Sclerostin Levels Correlate with Coronary Artery Calcification and Cardiovascular Events in Maintenance Hemodialysis Patients	irrelevent reports	Cardiology Research and Practice
Wang, K. T., Y. Y. Liu, K. T. Sung, C. C. Liu, C. H. Su, T. C. Hung, C. L. Hung, C. Y. Chien and H. I. Yeh	- 2020	Circulating monocyte count as a surrogate marker for ventricular-arterial remodeling and incident heart failure with preserved ejection fraction	irrelevent reports	Diagnostics
Baumhove, L., N. Bomer, J. Tromp, B. J. van Essen, K. Dickstein, J. G. Cleland, C. C. Lang, L. L. Ng, N. J. Samani, S. D. Anker, M. Metra, D. J. van Veldhuisen, P. van der Meer and A. A. Voors	- 2024	Clinical characteristics and prognosis of patients with heart failure and high concentrations of interleukin-17D	irrelevent reports	International Journal of Cardiology
Piotrowski, J., M. Timler, R. Kozłowski, A. Stasiak, J. Stasiak, A. Bissinger, D. Timler, W. Timler, M. Marczak, R. Załuska and G. Piotrowski	- 2021	Clinical Characteristics, Treatment, and Short-Term Outcome in Patients with Heart Failure and Cancer	irrelevent reports	Clinics and Practice
He, J., W. Yang, W. Wu, X. Sun, S. Li, G. Yin, B. Zhuang, J. Xu, D. Zhou, Y. Zhang, Y. Wang, L. Zhu, P. Sharma, A. Sirajuddin, Z. Teng, F. Kureshi, S. Zhao and M. Lu	- 2023	Clinical features, myocardial strain and tissue characteristics of heart failure with preserved ejection fraction in patients with obesity: A prospective cohort study	irrelevent reports	eClinicalMedicine

Song, S., J. S. Ko, H. A. Lee, E. K. Choi, M. J. Cha, T. H. Kim, J. K. Park, J. M. Lee, K. W. Kang, J. Shim, J. S. Uhm, J. Kim, C. Kim, J. B. Kim, H. W. Park, B. Joung and J. Park	- 2022	Clinical Implications of Heart Rate Control in Heart Failure With Atrial Fibrillation: Multi-Center Prospective Observation Registry (CODE-AF Registry)	irrelevent reports	Frontiers in Cardiovascular Medicine
Suzuki, S., A. Yoshihisa, Y. Sato, Y. Kanno, S. Watanabe, S. Abe, T. Sato, M. Oikawa, A. Kobayashi, T. Yamaki, H. Kunii, K. Nakazato, T. Ishida and Y. Takeishi	- 2018	Clinical significance of get with the guidelines-heart failure risk score in patients with chronic heart failure after hospitalization	irrelevent reports	Journal of the American Heart Association
Nishihara, T., E. Yamamoto, D. Sueta, K. Fujisue, H. Usuku, F. Oike, M. Takae, Y. Arima, S. Araki, S. Takashio, T. Nakamura, S. Suzuki, K. Sakamoto, H. Soejima, H. Kawano, K. Kaikita, K. Tsujita and L. Roever	- 2019	Clinical significance of serum magnesium levels in patients with heart failure with preserved ejection fraction	irrelevent reports	Medicine (United States)
Oeun, B., S. Hikoso, D. Nakatani, H. Mizuno, T. Kitamura, K. Okada, T. Dohi, Y. Sotomi, H. Kida, A. Sunaga, T. Sato, Y. Matsuoka, H. Kurakami, T. Yamada, S. Tamaki, M. Seo, M. Yano, T. Hayashi, A. Nakagawa, Y. Nakagawa, T. Yamada, Y. Yasumura and Y. Sakata	- 2023	Clinical trajectories and outcomes of patients with heart failure with preserved ejection fraction with normal or indeterminate diastolic function	irrelevent reports	Clinical Research in Cardiology
Cleland, J. G. F., A. P. Coletta and A. L. Clark	- 2006	Clinical trials update from the joint European Society and World Congress of Cardiology meeting: PEP-CHF, ACCLAIM and the HHH study	irrelevent reports	European Journal of Heart Failure
Parcha, V., N. Patel, R. Kalra, A. Bhargava, S. D. Prabhu, G. Arora and P. Arora	- 2020	Clinical, Demographic, and Imaging Correlates of Anemia in Heart Failure With Preserved Ejection Fraction (from the RELAX Trial)	irrelevent reports	American Journal of Cardiology
Schuermans, A., M. C. Honigberg, L. M. Raffield, B. Yu, M. B. Roberts, C. Kooperberg, P. Desai, A. P. Carson, A. M. Shah, C. M. Ballantyne, A. G. Bick, P. Natarajan, J. E. Manson, E. A. Whitsel, C. B. Eaton and A. P. Reiner	- 2024	Clonal Hematopoiesis and Incident Heart Failure With Preserved Ejection Fraction	irrelevent reports	JAMA Netw Open
Risco-Zevallos, J. D., J. Piñeyroa, D. Rodríguez-Espinosa, M. Garrote, A. Gaya, J. J. Broseta, L. F. Quintana, M. Blasco and M. V. Rivera	- 2022	Cloudy fluid, cloudy diagnosis	irrelevent reports	Peritoneal Dialysis International
Doeblin, P., D. Hashemi, R. Tanacli, T. Lapinskas, R. Gebker, C. Stehning, L. A. Motzkus, M. Blum, E. Tahirovic, A. Dordevic, R. Kraft, S. M. Zamani, B. Pieske, F. Edelmann, H. D. Düngen and S. Kelle	- 2019	CMR tissue characterization in patients with HFmrEF	irrelevent reports	Journal of Clinical Medicine
Yokota, J., Y. Ogawa, S. Yamanaka, Y. Takahashi, H. Fujita, N. Yamaguchi, N. Onoue, T. Ishizuka, T. Shinozaki and A. Kohzuki	- 2016	Cognitive dysfunction and malnutrition are independent predictor of dysphagia in patients with acute exacerbation of congestive heart failure	irrelevent reports	PLoS ONE
Shchendrygina, A., S. Rachina, N. Cherkasova, A. Suvorov, I. Komarova, N. Mukhina, N. Ananicheva, D. Gasanova, V. Sitnikova, A. Koposova, J. Smirnova, E. Moiseewa and D. Drogashevskaya	- 2023	Colchicine in patients with heart failure and preserved left ventricular ejection fraction: rationale and design of a prospective, randomised, open-label, crossover clinical trial	irrelevent reports	Open Heart
Prakash, S., M. Obeidat and A. R. Murali	- 2022	Colonic Venous Blebs Secondary to Gastrointestinal Amyloidosis	irrelevent reports	ACG Case Reports Journal

Wu, C. K., M. Y. M. Su, Y. F. Wu, J. J. Hwang and L. Y. Lin	-<br>2018	Combination of plasma biomarkers and clinical data for the detection of myocardial fibrosis or aggravation of heart failure symptoms in heart failure with preserved ejection fraction patients	irrelevent reports	Journal of Clinical Medicine
Yamasaki, Y., K. Suzuki, R. Kamijima, Y. Asari, K. Tsuchida, M. Mizushima, T. Okazaki, Y. J. Akashi, S. Ozaki and H. Yamada	-<br>2018	Combined disease with pulmonary arterial hypertension and pulmonary venous hypertension revealed after treatment of heart failure with preserved ejection fraction in a case with primary Sjögren syndrome	irrelevent reports	Modern Rheumatology
Fassett, R. G., R. Driver, H. Healy, D. Ranganathan, S. Ratanjee, I. K. Robertson, D. P. Geraghty, J. E. Sharman and J. S. Coombes	-<br>2009	Comparison of markers of oxidative stress, inflammation and arterial stiffness between incident hemodialysis and peritoneal dialysis patients--an observational study	irrelevent reports	BMC Nephrol
van Ramshorst, J., M. Duffels, S. P. M. de Boer, A. Bos-Schaap, O. Drexhage, S. Walburg, J. de Beij, D. van der Stoop and V. A. W. M. Umans	-<br>2022	Connected care for endocarditis and heart failure patients: a hospital-at-home programme	irrelevent reports	Netherlands Heart Journal
De Roeck, F., E. A. Prihadi, P. Vermeersch and Y. De Greef	-<br>2020	Constrictive pericarditis as late complication of cryoballoon pulmonary vein isolation	irrelevent reports	HeartRhythm Case Reports
Dai, T., D. Wu, J. Tang, Z. Liu and M. Zhang	-<br>2023	Construction and validation of a predictive model for the risk of three-month-postoperative malnutrition in patients with gastric cancer: a retrospective case-control study	irrelevent reports	Journal of Gastrointestinal Oncology
Kimmel, J., R. Potosky, M. R. Williams, M. Glading, P. J. Neuburger, J. D. Roberts and A. Feider	-<br>2018	Conversion from Monitored Anesthesia Care to General Anesthesia for Transcatheter Aortic Valve Replacement	irrelevent reports	Journal of Cardiothoracic and Vascular Anesthesia
Chiu, L., V. Agrawal, D. Armstrong, E. Brittain, S. Collins, A. R. Hemnes, J. A. Hill, J. Lindenfeld, S. J. Shah, L. W. Stevenson, T. J. Wang and D. K. Gupta	-<br>2024	Correlates of Plasma NT-proBNP/Cyclic GMP Ratio in Heart Failure With Preserved Ejection Fraction: An Analysis of the RELAX Trial	irrelevent reports	Journal of the American Heart Association
Li, T., L. Yang, S. Zhao and S. Zhang	-<br>2018	Correlation Between Apolipoprotein M and Inflammatory Factors in Obese Patients	irrelevent reports	Med Sci Monit
Xu, W., S. Liang, Y. Huang, S. Zhao, Y. Zhang and Y. Li	-<br>2021	Correlation between thyroid autoantibodies and cardiovascular disease in patients with stages 3–5 chronic kidney disease	irrelevent reports	Annals of Translational Medicine
Lourenço, P., J. Pereira, A. Ribeiro, J. Ferreira-Coimbra, I. Barroso, J. T. Guimarães, A. Leite-Moreira and P. Bettencourt	-<br>2019	C-reactive protein decrease associates with mortality reduction only in heart failure with preserved ejection fraction	irrelevent reports	J Cardiovasc Med (Hagerstown)
Celik, T., A. Iyisoy, M. Celik, U. C. Yuksel and E. Kardesoglu	-<br>2009	C-reactive protein in chronic heart failure: A new predictor of survival	irrelevent reports	International Journal of Cardiology
Villar, S., A. Mollar, M. Lorenzo, G. Núñez, R. de la Espriella and J. Núñez	-<br>2024	C-reactive protein in patients with acute heart failure and preserved ejection fraction	irrelevent reports	Revista Espanola de Cardiologia
Koller, L., M. Kleber, G. Goliasch, P. Sulzgruber, H. Scharnagl, G. Silbernagel, T. Grammer, G. Delgado, A. Tomaschitz, S. Pilz, W. März and A. Niessner	-<br>2014	C-reactive protein predicts mortality in patients referred for coronary angiography and symptoms of heart failure with preserved ejection fraction	irrelevent reports	Eur J Heart Fail
Williams, E. S., S. J. Shah, S. Ali, B. Y. Na, N. B. Schiller and M. A. Whooley	-<br>2008	C-reactive protein, diastolic dysfunction, and risk of heart failure in patients with coronary disease: Heart and Soul Study	irrelevent reports	Eur J Heart Fail
Brassington, K., P. Kanellakis, A. Cao, B. H. Toh, K. Peter, A. Bobik and T. Kyaw	-<br>2022	Crosstalk between cytotoxic CD8+ T cells and stressed cardiomyocytes triggers development of interstitial cardiac fibrosis in hypertensive mouse hearts	irrelevent reports	Front Immunol
Joseph, J. P., E. Reyes, J. Guzman, J. O'Doherty, H. McConkey, S. Arri, R. Kakkar, N. Beckley, A. Douiri, S. F. Barrington, S. R. Redwood and A. Ferro	-<br>2017	CXCR2 Inhibition - a novel approach to treating CoronAry heart DiseAse (CICADA): study protocol for a randomised controlled trial	irrelevent reports	Trials

Lin, Y., X. Hu, W. Wang, B. Yu, L. Zhou, Y. Zhou, G. Li and H. Dong	- 2022	D-Dimer Is Associated With Coronary Microvascular Dysfunction in Patients With Non-obstructive Coronary Artery Disease and Preserved Ejection Fraction	irrelevent reports	Frontiers in Cardiovascular Medicine
Chiang, C. H., P. H. Huang, H. B. Leu, C. Y. Hsu, K. F. Wang, J. W. Chen and S. J. Lin	- 2013	Decreased circulating endothelial progenitor cell levels in patients with heart failure with preserved ejection fraction	irrelevent reports	Cardiology (Switzerland)
Gao, X. R., M. D. Wang, X. Y. He, L. L. Tang, J. B. Liang, L. Y. Peng, H. Lin, H. W. Sun, H. Ma, J. Lin and Z. Y. Zuo	- 2011	Decreased intralymphocytic magnesium content is associated with diastolic heart dysfunction in patients with essential hypertension	irrelevent reports	Int J Cardiol
Tourki, B., V. Kain, S. R. Shaikh, X. Leroy, C. N. Serhan and G. V. Halade	- 2020	Deficit of resolution receptor magnifies inflammatory leukocyte directed cardiorenal and endothelial dysfunction with signs of cardiomyopathy of obesity	irrelevent reports	Faseb j
Klimis, H., M. Altman, R. Chard, M. Skinner and L. Thomas	- 2019	Dehiscence of a Mechanical Aortic Valve Secondary to Culture-Negative Endocarditis Complicated by Acute Heart Failure	irrelevent reports	CASE
Vu, K., B. L. Claggett, J. E. John, H. Skali, S. D. Solomon, T. H. Mosley, J. E. Williams, A. Kucharska-Newton, T. Biering-Sørensen and A. M. Shah	- 2021	Depressive symptoms, cardiac structure and function, and risk of incident heart failure with preserved ejection fraction and heart failure with reduced ejection fraction in late life	irrelevent reports	Journal of the American Heart Association
van Wezenbeek, J., J. M. Canada, K. Ravindra, S. Carbone, D. Kadariya, C. R. Trankle, G. Wohlford, L. Buckley, M. G. Del Buono, H. Billingsley, M. Viscusi, I. Tchoukina, K. B. Shah, R. Arena, B. Van Tassell and A. Abbate	- 2020	Determinants of Cardiorespiratory Fitness in Patients with Heart Failure Across a Wide Range of Ejection Fractions	irrelevent reports	American Journal of Cardiology
Pan, Q., C. Chen, Z. Gong, G. Chen and Y. Yang	- 2023	Development of heart failure with preserved ejection fraction is independent of eosinophils in a preclinical model	irrelevent reports	Immun Inflamm Dis
Annappah, D., M. Saling, J. Prodafikas and A. N. Badie	- 2022	Device-associated aortic valve endocarditis due to a complicated Enterobacter cloacae urinary tract infection	irrelevent reports	IDCases
Cunha, F. M., J. Pereira, P. Marques, A. Ribeiro, P. Bettencourt and P. Lourenço	- 2020	Diabetic patients need higher furosemide doses: A report on acute and chronic heart failure patients	irrelevent reports	Journal of Cardiovascular Medicine
Dong, J., D. Barnett, N. Adjei-Kyeremeh and H. Bartz	- 2022	Diagnosing Intravascular Large B-Cell Lymphoma: A Tale of Hide and Seek	irrelevent reports	Case Reports in Oncology
Chan, W. X., A. Kaura, A. Mulla, D. Papadimitriou, B. Glampson, E. Mayer, A. S. V. Shah, J. Mayet and C. H. Yap	- 2024	Diagnostic and prognostic value of an ejection fraction adjusted for myocardial remodeling	irrelevent reports	Frontiers in Cardiovascular Medicine
Lakhani, I., M. V. Wong, J. K. F. Hung, M. Gong, K. B. Waleed, Y. Xia, S. Lee, L. Roever, T. Liu, G. Tse, K. S. K. Leung and K. H. C. Li	- 2021	Diagnostic and prognostic value of serum C-reactive protein in heart failure with preserved ejection fraction: a systematic review and meta-analysis	irrelevent reports	Heart Fail Rev
Hookey, G., Q. Ahmad, T. McCune, J. Kowalewska, B. Amaker and N. Inayat	- 2021	Diagnostic role of technitium-99m bone scan in severe COVID-19-associated myositis	irrelevent reports	Radiology Case Reports
Farajidavar, N., K. O'Gallagher, D. Bean, A. Nabeebaccus, R. Zakeri, D. Bromage, Z. Kraljevic, J. T. H. Teo, R. J. Dobson and A. M. Shah	- 2022	Diagnostic signature for heart failure with preserved ejection fraction (HFpEF): a machine learning approach using multi-modality electronic health record data	irrelevent reports	BMC Cardiovascular Disorders
Polat, V., E. Bozcali, T. Uygun, S. Opan and O. Karakaya	- 2016	Diagnostic significance of serum galectin-3 levels in heart failure with preserved ejection fraction	irrelevent reports	Acta Cardiologica
Yu, P., M. Liu, X. Yang, Y. Yu, J. Zhao, L. Zhang, R. Tong, H. Jiang, Y. Zou and J. Ge	- 2016	Diagnostic utility of ANG in coronary heart disease complicating chronic heart failure: A cross-sectional study	irrelevent reports	Disease Markers
Cho, W., T. Y. Hwang, Y. K. Choi, J. H. Yang, M. G. Kim, S. K. Jo, W. Y. Cho and S. W. Oh	- 2019	Diastolic dysfunction and acute kidney injury in elderly patients with femoral neck fracture	irrelevent reports	Kidney Research and Clinical Practice

Srivastava, P. M., M. C. Thomas, P. Calafiore, R. J. MacIsaac, G. Jerums and L. M. Burrell	- 2006	Diastolic dysfunction is associated with anaemia in patients with Type II diabetes	irrelevent reports	Clin Sci (Lond)
Wernhart, S., A. Goertz, J. Hedderich, M. Papathanasiou, J. Hoffmann, T. Rassaf and P. Luedike	- 2023	Diastolic exercise stress testing in heart failure with preserved ejection fraction: The DEST-HF study	irrelevent reports	European Journal of Heart Failure
de Denus, S., J. Lavoie, A. Ducharme, E. O'Meara, N. Racine, M. G. Sirois, P. E. Neagoe, L. Zhu, J. L. Rouleau and M. White	- 2012	Differences in biomarkers in patients with heart failure with a reduced vs a preserved left ventricular ejection fraction	irrelevent reports	Can J Cardiol
Matsushita, K., K. Harada, T. Miyazaki, T. Miyamoto, S. Kohsaka, K. Iida, S. Tanimoto, M. Takei, T. Hosoda, Y. Yamamoto, Y. Shiraishi, H. Yoshino, T. Yamamoto, K. Nagao and M. Takayama	- 2019	Different prognostic associations of beta-blockers and diuretics in heart failure with preserved ejection fraction with versus without high blood pressure	irrelevent reports	Journal of Hypertension
Cui, Y., X. Qi, A. Huang, J. Li, W. Hou and K. Liu	- 2018	Differential and predictive value of galectin-3 and soluble suppression of tumorigenicity-2 (sST2) in heart failure with preserved ejection fraction	irrelevent reports	Medical Science Monitor
Hanvivadhanakul, P. and A. Buakhamsri	- 2019	Disease activity is associated with LV dysfunction in rheumatoid arthritis patients without clinical cardiovascular disease	irrelevent reports	Adv Rheumatol
Damilano, F., I. Franco, C. Perrino, K. Schaefer, O. Azzolino, D. Carnevale, G. Cifelli, P. Carullo, R. Ragona, A. Ghigo, A. Perino, G. Lembo and E. Hirsch	- 2011	Distinct effects of leukocyte and cardiac phosphoinositide 3-kinase γ activity in pressure overload-induced cardiac failure	irrelevent reports	Circulation
Grandi, E., M. F. Navedo, J. J. Saucerman, D. M. Bers, N. Chiamvimonvat, R. E. Dixon, D. Dobrev, A. M. Gomez, O. F. Harraz, B. Hegyi, D. K. Jones, T. Krogh-Madsen, W. L. Murfee, M. A. Nystoriak, N. G. Posnack, C. M. Ripplinger, R. Veeraraghavan and S. Weinberg	- 2023	Diversity of cells and signals in the cardiovascular system	irrelevent reports	Journal of Physiology
Hiraiwa, H., G. Sakamoto, R. Ito, Y. Koyama, S. Kazama, Y. Kimura, T. Kondo, R. Morimoto, T. Okumura and T. Murohara	- 2023	Dynamic chest radiography as a novel minimally invasive hemodynamic imaging method in patients with heart failure	irrelevent reports	European Journal of Radiology
Vasarmidi, E., N. Bizymi, A. M. Matthaiou, M. Tsagkataki, E. Bibaki, D. Tamiolakis, C. Skiadas, N. Tzanakis and K. M. Antoniou	- 2024	Dyspnea and crazy paving on computed tomography: Is it easy to make the diagnosis?	irrelevent reports	Pneumon
Villa, M. P., F. Ianniello, G. Tocci, M. Evangelisti, S. Miano, A. Ferrucci, G. M. Ciavarella and M. Volpe	- 2012	Early cardiac abnormalities and increased C-reactive protein levels in a cohort of children with sleep disordered breathing	irrelevent reports	Sleep Breath
Santos, A. B., M. Junges, D. Silvello, A. Macari, B. S. Araújo, B. G. Seligman, B. B. Duncan, L. E. Rohde, N. Clausell and M. Foppa	- 2013	Early change of extracellular matrix and diastolic parameters in metabolic syndrome	irrelevent reports	Arq Bras Cardiol
Urbieta Caceres, V. H., J. Lin, X. Y. Zhu, F. D. Favreau, M. E. Gibson, J. A. Crane, A. Lerman and L. O. Lerman	- 2011	Early experimental hypertension preserves the myocardial microvasculature but aggravates cardiac injury distal to chronic coronary artery obstruction	irrelevent reports	Am J Physiol Heart Circ Physiol

Doiron, J. E., Z. Li, X. Yu, K. B. LaPenna, H. Quiriarte, T. D. Allerton, K. Koul, A. Malek, S. J. Shah, T. E. Sharp, T. T. Goodchild, D. R. Kapusta and D. J. Lefer	- 2024	Early Renal Denervation Attenuates Cardiac Dysfunction in Heart Failure With Preserved Ejection Fraction	irrelevent reports	J Am Heart Assoc
Ališauskas, A., A. Naudžiūnas, S. Sadauskas, L. Jankauskienė, E. Kalinauskienė, J. Jucevičius and G. Vanagaitė	- 2023	Early repolarization pattern on ECG predicts worse outcomes in patients hospitalized due to chronic heart failure decompensation	irrelevent reports	Journal of Electrocardiology
Liu, Z., W. Hu, H. Zhang, H. Tao, P. Lei, J. Liu, Y. Yu, Q. Dong, L. Gao and D. Zhang	- 2022	EAT Thickness as a Predominant Feature for Evaluating Arterial Stiffness in Patients with Heart Failure with Preserved Ejection Fraction	irrelevent reports	Diabetes, Metabolic Syndrome and Obesity
Brittain, E. L., C. Nwabuo, M. Xu, D. K. Gupta, A. R. Hemnes, H. T. Moreira, H. D. De Vasconcellos, J. G. Terry, J. J. Carr and J. A. Lima	- 2017	Echocardiographic Pulmonary Artery Systolic Pressure in the Coronary Artery Risk Development in Young Adults (CARDIA) Study: Associations With Race and Metabolic Dysregulation	irrelevent reports	J Am Heart Assoc
Vetrovsky, T., M. Siranec, J. Parenica, M. Griva, J. Stastny, J. Precek, R. Pelouch, V. Bunc, A. Linhart and J. Belohlavek	- 2017	Effect of a 6-month pedometer-based walking intervention on functional capacity in patients with chronic heart failure with reduced (HFrEF) and with preserved (HFpEF) ejection fraction: Study protocol for two multicenter randomized controlled trials	irrelevent reports	Journal of Translational Medicine
Toh, N., K. Ishii, H. Kihara, K. Iwakura, H. Watanabe, J. Yoshikawa, H. Ito, S. Takiuchi, N. Tokunaga, M. Kusaka, F. Nakamura, S. Fukuda, Y. Abe, Y. Tani, A. Furukawa, Y. Hen, M. Kawaguchi, S. Mori, N. Kasayuki, H. Nishioka, Y. Doi, M. Ikushima, K. Suwa, K. Tawarahara, R. Terasaka, N. Takahashi, Y. Takeuchi, T. Minagawa, H. Takase, T. Nagano, Y. Hiramoto, M. Izumi, T. Yamada, H. Fujio, T. Takami, M. Yoshida, H. Oe, Y. Akazawa and T. Murakami	- 2016	Effect of diuretic or calcium-channel blocker plus angiotensin-receptor blocker on diastolic function in hypertensive patients	irrelevent reports	Circulation Journal
Beeres, S. L., H. J. Lamb, S. D. Roes, E. R. Holman, T. A. Kaandorp, W. E. Fibbe, A. de Roos, E. E. van der Wall, M. J. Schalij, J. J. Bax and D. E. Atsma	- 2008	Effect of intramyocardial bone marrow cell injection on diastolic function in patients with chronic myocardial ischemia	irrelevent reports	J Magn Reson Imaging
Dong, X., G. Zou, Y. Han, Y. Han and L. Liu	- 2023	Effect of Jiawei Shenqi Yixin Prescription on Cardiovascular Risk Factors in Patients with Heart Failure with Preserved Ejection Fraction and Insulin Resistance	irrelevent reports	Chinese Journal of Experimental Traditional Medical Formulae
Tatar, S., Y. E. Yavuz, İ. Oktay, N. Aydın, Y. Alsancak and A. İçli	- 2023	Effect of Pericardial Effusion on Right Ventricular Functions in Oncology Patients Receiving Chemotherapy	irrelevent reports	Istanbul Medical Journal
Gevaert, A. B., B. Böhm, H. Hartmann, I. Goovaerts, T. Stoop, C. M. Van De Heyning, P. J. Beckers, F. Baldassarri, S. Mueller, R. Oberhoffer, A. Duvinage, M. J. Haykowsky, U. Wisløff, V. Adams, B. Pieske, M. Halle and E. M. Van Craenenbroeck	- 2023	Effect of Training on Vascular Function and Repair in Heart Failure With Preserved Ejection Fraction	irrelevent reports	JACC: Heart Failure
Ensley, R. D., M. Ives, L. Zhao, M. McMillan, J. Shelby and W. H. Barry	- 1994	Effects of alloimmune injury on contraction and relaxation in cultured myocytes and intact cardiac allografts	irrelevent reports	J Am Coll Cardiol
Gunes, Y., M. Tuncer, U. Guntekin, M. Sahin and L. Yazmalar	- 2009	Effects of ankylosing spondylitis on the heart	irrelevent reports	Acta Cardiol
Wu, C. K., C. F. Yeh, J. Y. Chiang, T. T. Lin, Y. F. Wu, C. K. Chiang, T. W. Kao, K. Y. Hung and J. W. Huang	- 2017	Effects of atorvastatin treatment on left ventricular diastolic function in peritoneal dialysis patients-The ALEVENT clinical trial	irrelevent reports	J Clin Lipidol

Prochaska, J. H., C. Jünger, A. Schulz, N. Arnold, F. Müller, M. W. Heidorn, R. Baumkötter, D. Zahn, T. Koeck, S. O. Tröbs, K. J. Lackner, A. Daiber, H. Binder, S. J. Shah, T. Gori, T. Münzel and P. S. Wild	- 2023	Effects of empagliflozin on left ventricular diastolic function in addition to usual care in individuals with type 2 diabetes mellitus—results from the randomized, double-blind, placebo-controlled EmDia trial	irrelevent reports	Clinical Research in Cardiology
Van Tassell, B. W., R. Arena, G. Biondi-Zoccai, J. McNair Canada, C. Oddi, N. A. Abouzaki, A. Jahangiri, R. A. Falcao, M. C. Kontos, K. B. Shah, N. F. Voelkel, C. A. Dinarello and A. Abbate	- 2014	Effects of interleukin-1 blockade with anakinra on aerobic exercise capacity in patients with heart failure and preserved ejection fraction (from the D-HART pilot study)	irrelevent reports	American Journal of Cardiology
Kosiborod, M. N., S. Verma, B. A. Borlaug, J. Butler, M. J. Davies, T. Jon Jensen, S. Rasmussen, P. Erlang Marstrand, M. C. Petrie, S. J. Shah, H. Ito, M. Schou, V. Melenovský, W. Abhayaratna and D. W. Kitzman	- 2024	Effects of Semaglutide on Symptoms, Function, and Quality of Life in Patients With Heart Failure With Preserved Ejection Fraction and Obesity: A Prespecified Analysis of the STEP-HFpEF Trial	irrelevent reports	Circulation
Kang, J., H. J. Cho, H. Y. Lee, S. Lee, S. K. Park, S. E. Lee, J. J. Kim, E. S. Jeon, S. C. Chae, S. H. Baek, S. M. Kang, D. J. Choi, B. S. Yoo, K. H. Kim, M. C. Cho and B. H. Oh	- 2018	Effects of widespread inotrope use in acute heart failure patients	irrelevent reports	Journal of Clinical Medicine
Rehman, A., S. Saidullah, M. Asad, U. R. Gondal, A. Ashraf, M. F. Khan, W. Akhtar, A. Mehmoodi and J. Malik	- 2024	Efficacy and safety of semaglutide in patients with heart failure with preserved ejection fraction and obesity	irrelevent reports	Clinical Cardiology
Kim, S. H., W. Y. Chung, J. H. Zo, M. A. Kim, H. J. Chang, Y. S. Cho, T. J. Youn, I. H. Chae, D. J. Choi, J. J. Gwak, H. Y. Lee, J. S. Park, H. J. Kang, Y. J. Kim and H. S. Kim	- 2009	Efficacy and tolerability of two formulations of ramipril in Korean adults with mild to moderate essential hypertension: an 8-week, multicenter, prospective, randomized, open-label, parallel-group noninferiority trial	irrelevent reports	Clin Ther
Masaki, M., K. Komamura, A. Goda, S. Hirotani, M. Otsuka, A. Nakabo, M. Fukui, S. Fujiwara, M. Sugahara, M. Lee-Kawabata, T. Tsujino, M. Koshiba and T. Masuyama	- 2014	Elevated arterial stiffness and diastolic dysfunction in subclinical hypothyroidism	irrelevent reports	Circ J
Grufman, H., T. Yndigegn, I. Gonçalves, J. Nilsson and A. Schiopu	- 2019	Elevated IL-27 in patients with acute coronary syndrome is associated with adverse ventricular remodeling and increased risk of recurrent myocardial infarction and cardiovascular death	irrelevent reports	Cytokine
Petyunina, O. V., M. P. Kopytsya and A. E. Berezin	- 2018	Elevated levels of circulating soluble ST2 at discharge predict late adverse ventricular remodeling in patients with ST-segment elevation myocardial infarction	irrelevent reports	Biomedical Research and Therapy
Hsieh, M. C., W. C. Yu, C. C. Weng, W. J. Chen, C. K. Chen, Y. C. Lee and M. H. Chen	- 2023	Elevated serum levels of T-cell immunoglobulin and mucin-domain containing molecule 3 in patients with systemic inflammation following COVID-19 vaccination	irrelevent reports	Journal of the Chinese Medical Association
Carnicelli, A. P., J. L. Sun, B. Alhanti, M. Bjursell, S. Perl, B. Lytle, M. T. Roe and R. J. Mentz	- 2020	Elevated Uric Acid Prevalence and Clinical Outcomes in Patients with Heart Failure with Preserved Ejection Fraction: Insights from RELAX	irrelevent reports	American Journal of Medicine
Zhao, D., E. Guallar, P. Ouyang, V. Subramanya, D. Vaidya, C. E. Ndumele, J. A. Lima, M. A. Allison, S. J. Shah, A. G. Bertoni, M. J. Budoff, W. S. Post and E. D. Michos	- 2018	Endogenous Sex Hormones and Incident Cardiovascular Disease in Post-Menopausal Women	irrelevent reports	Journal of the American College of Cardiology

Hahn, V. S., L. R. Yanek, J. Vaishnav, W. Ying, D. Vaidya, Y. Z. J. Lee, S. J. Riley, V. Subramanya, E. E. Brown, C. D. Hopkins, S. Ononogbu, K. Perzel Mandell, M. K. Halushka, C. Steenbergen, A. Z. Rosenberg, R. J. Tedford, D. P. Judge, S. J. Shah, S. D. Russell, D. A. Kass and K. Sharma	- 2020	Endomyocardial Biopsy Characterization of Heart Failure With Preserved Ejection Fraction and Prevalence of Cardiac Amyloidosis	irrelevent reports	JACC: Heart Failure
Conte, L., M. Fejzo, A. Rossi, M. Zuin and L. Roncon	- 2018	Endomyocardial Fibrosis: A Rare Case of Diastolic Heart Failure in a European Caucasian Elderly Woman	irrelevent reports	Heart Lung and Circulation
Barroso, M. C., P. Boehme, F. Kramer, T. Mondritzki, T. Koehler, J. E. Gülker, M. Karoff and W. Dinh	- 2017	Endostatin a potential biomarker for heart failure with preserved ejection fraction	irrelevent reports	Arquivos Brasileiros de Cardiologia
Ärnlöv, J., Y. Sang, S. H. Ballew, D. Vaidya, E. D. Michos, D. R. Jacobs, J. Lima, M. G. Shlipak, A. G. Bertoni, J. Coresh, M. Blaha, W. S. Post and K. Matsushita	- 2020	Endothelial dysfunction and the risk of heart failure in a community-based study: the Multi-Ethnic Study of Atherosclerosis	irrelevent reports	ESC Heart Failure
Nesti, L., N. R. Pugliese, M. Chiriacò, D. Trico, S. Baldi and A. Natali	- 2023	Epicardial adipose tissue thickness is associated with reduced peak oxygen consumption and systolic reserve in patients with type 2 diabetes and normal heart function	irrelevent reports	Diabetes, Obesity and Metabolism
Jiang, Y., S. Hu, M. Cao, X. Li, J. Zhou, B. Ding, F. Zhang, T. Chen and Y. Zhou	- 2019	Evaluation of acute myocardial infarction patients with mid-range ejection fraction after emergency percutaneous coronary intervention	irrelevent reports	Postgraduate Medical Journal
Kis, M., Y. Dogan, A. Yildirim, T. Güzel, L. Bekar, O. Akhan, M. Dogdus, H. Harbalıoğlu, D. Karabulut, E. Soydan, M. Zoghi and O. Ergene	- 2022	Evaluation of demographic, clinical, and aetiological data of patients admitted to cardiology clinics and diagnosed with left ventricular hypertrophy in Turkish population (LVH-TR)	irrelevent reports	Acta Cardiologica
Besler, C., K. P. Rommel, K. P. Kresoja, J. Mörbitz, H. Kirsten, M. Scholz, K. Klingel, J. Thiery, R. Burkhardt, P. Büttner, V. Adams, H. Thiele and P. Lurz	- 2021	Evaluation of phosphodiesterase 9A as a novel biomarker in heart failure with preserved ejection fraction	irrelevent reports	ESC Heart Fail
Agarwal, A., S. Beddhu, R. Boucher, V. Rao, N. Ramkumar, A. R. Rodan, J. Fang, B. M. Wynne, S. G. Drakos, T. Hanff, A. K. Cheung and J. C. Fang	- 2024	Evaluation of renal sodium handling in heart failure with preserved ejection fraction: A pilot study	irrelevent reports	Physiological Reports
Erturk, I., E. Sertoglu, C. Bilgi, K. Saglam, F. Yesildal, R. Acar and T. Ozgurtas	- 2019	Evaluation of the effects of different treatment modalities on angiogenesis in heart failure patients with preserved ejection fraction via VEGF and sVEGFR-1	irrelevent reports	Turkish Journal of Biochemistry
Glezeva, N., V. Voon, C. Watson, S. Horgan, K. McDonald, M. Ledwidge and J. Baugh	- 2015	Exaggerated inflammation and monocytosis associate with diastolic dysfunction in heart failure with preserved ejection fraction: evidence of M2 macrophage activation in disease pathogenesis	irrelevent reports	J Card Fail
Pugliese, N. R., M. Mazzola, R. Madonna, L. Gargani, N. De Biase, F. L. Dini, S. Taddei, R. De Caterina and S. Masi	- 2022	Exercise-induced pulmonary hypertension in HFpEF and HFrEF: Different pathophysiologic mechanism behind similar functional impairment	irrelevent reports	Vascular Pharmacology
Carson, M. A., J. Reid, L. Hill, L. Dixon, P. Donnelly, P. Slater, A. Hill, S. E. Piper, T. A. McDonagh and D. Fitzsimons	- 2022	Exploring the prevalence, impact and experience of cardiac cachexia in patients with advanced heart failure and their caregivers: A sequential phased study	irrelevent reports	Palliative Medicine
Ishikawa, H., K. Otsuka, Y. Kono, K. Hojo, H. Yamaura, K. Hirata, N. Kasayuki, Y. Izumiya and D. Fukuda	- 2023	Extent of coronary atherosclerosis is associated with deterioration of left ventricular global longitudinal strain in patients with preserved ejection fraction undergoing coronary computed tomography angiography	irrelevent reports	IJC Heart and Vasculature
Karim, B., I. P. Wijaya, R. Rahmaniyah, I. Ariyanto, S. Waters, R. Estiasari and P. Price	- 2017	Factors affecting affect cardiovascular health in Indonesian HIV patients beginning ART	irrelevent reports	AIDS Res Ther
Shurkevich, N. P., A. S. Vetoshkin, A. A. Simonyan, L. I. Gapon and M. A. Kareva	- 2023	Factors and sex differences associated with intermediate probability of heart failure with preserved ejection fraction in asymptomatic patients working in the Arctic	irrelevent reports	Russian Journal of Cardiology

Komajda, M., P. E. Carson, S. Hetzel, R. McKelvie, J. McMurray, A. Ptaszynska, M. R. Zile, D. Demets and B. M. Massie	- 2011	Factors associated with outcome in heart failure with preserved ejection fraction: findings from the Irbesartan in Heart Failure with Preserved Ejection Fraction Study (I-PRESERVE)	irrelevent reports	Circ Heart Fail
Barker, C. M., C. U. Meduri, P. S. Fail, J. W. Chambers, D. J. Solet, J. M. Kriegel, D. C. Vela, K. Feldt, T. D. Pate, A. P. Patel and T. Shaburishvili	- 2022	Feasibility of a No-Implant Approach to Interatrial Shunts: Preclinical and Early Clinical Studies	irrelevent reports	Structural Heart
Manukyan, M. A., A. Y. Falkovskaya, V. F. Mordovin, T. R. Ryabova, I. V. Zyubanova, A. M. Gusakova and T. E. Suslova	- 2021	Features of heart failure with preserved ejection fraction (HFPEF) in diabetic patients with resistant hypertension	irrelevent reports	Diabetes Mellitus
Ghuman, J., X. Cai, R. B. Patel, S. S. Khan, J. Hecktman, M. M. Redfield, G. Lewis, S. J. Shah, M. Wolf, T. Isakova and R. Mehta	- 2021	Fibroblast Growth Factor 23 and Exercise Capacity in Heart Failure with Preserved Ejection Fraction: FGF23 and Exercise Capacity in HFpEF	irrelevent reports	Journal of Cardiac Failure
Kanagala, P., J. R. Arnold, J. N. Khan, A. Singh, G. S. Gulsin, M. Eltayeb, P. Gupta, I. B. Squire, G. P. McCann and L. L. Ng	- 2020	Fibroblast-growth-factor-23 in heart failure with preserved ejection fraction: relation to exercise capacity and outcomes	irrelevent reports	ESC Heart Failure
Sablani, N., J. Garg, B. Hasan, R. Patel and M. W. Martinez	- 2018	First reported case series in the United States of hemopericardium in patients on apixaban	irrelevent reports	HeartRhythm Case Reports
Gu, Z. Y., B. H. Chen, L. Zhao, D. A. An, C. W. Wu, S. Xue, W. B. Chen, S. Huang, Y. Y. Wang and L. M. Wu	- 2024	Fractal analysis of left ventricular trabeculae in heart failure with preserved ejection fraction patients with multivessel coronary artery disease	irrelevent reports	Insights into Imaging
Nomura, S. O., A. B. Karger, N. L. Weir, J. A. C. Lima, G. Thanassoulis and M. Y. Tsai	- 2021	Free fatty acids and heart failure in the Multi-Ethnic Study of Atherosclerosis (MESA)	irrelevent reports	Journal of Clinical Lipidology
Casian, M., C. Jurcuţ, C. Dobrea, B. Radulescu and R. Jurcuţ	- 2023	From clinic to echocardiography to microscope - The multimodal journey of a rare disease	irrelevent reports	Romanian Journal of Cardiology / Revista Romana de Cardiologie
Veronese, G., S. Nonini, A. Cannata, F. Aresta, G. Olivieri, E. Montrasio, D. De Caria, E. Perna, A. Calini, M. Bottiroli, F. Cislaghi, G. Pedrazzini, F. Baltaro, G. Quattrocchi, P. Pedrotti, C. F. Russo, A. Garascia, M. Mondino and E. Ammirati	- 2022	Fulminant Lymphocytic Myocarditis during Pregnancy Treated with Temporary Mechanical Circulatory Supports and Aggressive Immunosuppression	irrelevent reports	Circulation: Heart Failure
AbouEzzeddine, O. F., P. Haines, S. Stevens, J. Nativi-Nicolau, G. M. Felker, B. A. Borlaug, H. H. Chen, R. P. Tracy, E. Braunwald and M. M. Redfield	- 2015	Galectin-3 in heart failure with preservedejection fraction. A RELAX trial substudy (phosphodiesterase-5 inhibition to improve clinical status and exercise capacity in diastolic heartfailure)	irrelevent reports	JACC: Heart Failure
Tousoulis, D., E. Androulakis, N. Papageorgiou, A. Miliou, E. Chatzistamatiou, E. Oikonomou, G. Moustakas, I. Kallikazaros and C. Stefanadis	- 2013	Genetic predisposition to left ventricular hypertrophy and the potential involvement of cystatin-C in untreated hypertension	irrelevent reports	Am J Hypertens
Nishi, I., Y. Seo, Y. Hamada-Harimura, M. Yamamoto, T. Ishizu, A. Sugano, K. Sato, S. Sai, K. Obara, S. Suzuki, A. Koike, K. Aonuma and M. Ieda	- 2019	Geriatric nutritional risk index predicts all-cause deaths in heart failure with preserved ejection fraction	irrelevent reports	ESC Heart Failure
Kinugasa, Y., M. Kato, S. Sugihara, M. Hirai, K. Yamada, K. Yanagihara and K. Yamamoto	- 2013	Geriatric nutritional risk index predicts functional dependency and mortality in patients with heart failure with preserved ejection fraction	irrelevent reports	Circ J

Yasuda, Y., H. Aoki, W. Fujita, K. Fujibayashi, M. Wakasa, Y. Kawai, H. Nakanishi, K. Saito, M. Takeuchi and K. Kajinami	- 2022	Glyceraldehyde-derived advanced glycation end-products are associated with left ventricular ejection fraction and brain natriuretic peptide in patients with diabetic adverse cardiac remodeling	irrelevent reports	Scandinavian Cardiovascular Journal
Mohiuddin, M. M., A. K. Singh, L. Scobie, C. E. Goerlich, A. Grazioli, K. Saharia, C. Crossan, A. Burke, C. Drachenberg, C. Oguz, T. Zhang, B. Lewis, A. Hershfeld, F. Sentz, I. Tatarov, S. Mudd, G. Braileanu, K. Rice, J. F. Paolini, K. Bondensgaard, T. Vaught, K. Kuravi, L. Sorrells, A. Dandro, D. Ayares, C. Lau and B. P. Griffith	- 2023	Graft dysfunction in compassionate use of genetically engineered pig-to-human cardiac xenotransplantation: a case report	irrelevent reports	Lancet
Gizatulina, T. P., L. U. Martyanova, T. I. Petelina, E. V. Zueva and N. E. Shirokov	- 2021	GROWTH DIFFERENTIATION FACTOR 15 AS AN INTEGRAL MARKER OF CLINICAL AND FUNCTIONAL STATUS OF PATIENTS WITH NON-VALVULAR ATRIAL FIBRILLATION	irrelevent reports	Journal of Arrhythmology
Hayashi, T., T. Yamashita, H. Watanabe, K. Kami, N. Yoshida, T. Tabata, T. Emoto, N. Sasaki, T. Mizoguchi, Y. Irino, R. Toh, M. Shinohara, Y. Okada, W. Ogawa, T. Yamada and K. I. Hirata	- 2019	Gut microbiome and plasma microbiome-related metabolites in patients with decompensated and compensated heart failure	irrelevent reports	Circulation Journal
Sun, L., X. Liu, W. Li and D. Jia	- 2019	HDL-C to hsCRP ratio is associated with left ventricular diastolic function in absence of significant coronary atherosclerosis	irrelevent reports	Lipids Health Dis
Alvi, R. M., M. Afshar, A. M. Neilan, N. Tariq, M. Hassan, J. Gerber, M. Awadalla, C. P. Mulligan, A. Rokicki, V. A. Triant, M. V. Zanni and T. G. Neilan	- 2019	Heart failure and adverse heart failure outcomes among persons living with HIV in a US tertiary medical center	irrelevent reports	American Heart Journal
Wang, A., Z. Li, Z. Sun, Y. Wang, S. Fu, D. Zhang and X. Ma	- 2023	Heart failure with preserved ejection fraction and non-alcoholic fatty liver disease: new insights from bioinformatics	irrelevent reports	ESC Heart Failure
Chitsazan, M., A. Amin, M. Chitsazan, N. Ziaie, P. Amri Maleh, H. Pouraliakbar and S. Von Haehling	- 2021	Heart failure with preserved ejection fraction in coronavirus disease 2019 patients: the promising role of diuretic therapy in critically ill patients	irrelevent reports	ESC Heart Failure
Pop-Busui, R., J. L. Januzzi, D. Bruemmer, S. Butalia, J. B. Green, W. B. Horton, C. Knight, M. Levi, N. Rasouli and C. R. Richardson	- 2022	Heart Failure: An Underappreciated Complication of Diabetes. A Consensus Report of the American Diabetes Association	irrelevent reports	Diabetes Care
Yano, M., M. Nishino, K. Ukita, A. Kawamura, H. Nakamura, Y. Matsuhiro, K. Yasumoto, M. Tsuda, N. Okamoto, A. Tanaka, Y. Matsunaga-Lee, Y. Egami, R. Shutta, J. Tanouchi, T. Yamada, Y. Yasumura, S. Tamaki, T. Hayashi, A. Nakagawa, Y. Nakagawa, S. Suna, D. Nakatani, S. Hikoso and Y. Sakata	- 2021	High density lipoprotein cholesterol / C reactive protein ratio in heart failure with preserved ejection fraction	irrelevent reports	ESC Heart Fail
Kimura, M., H. Sekiguchi, K. Shimamoto, M. Kawana, Y. Takemura, N. Hagiwara and J. Yamaguchi	- 2022	High-normal diastolic blood pressure as a risk factor for left ventricular diastolic dysfunction in healthy postmenopausal women	irrelevent reports	Hypertension Research
Pellicori, P., J. Zhang, J. Cuthbert, A. Urbinati, P. Shah, S. Kazmi, A. L. Clark and J. G. F. Cleland	- 2020	High-sensitivity C-reactive protein in chronic heart failure: Patient characteristics, phenotypes, and mode of death	irrelevent reports	Cardiovascular Research
DuBrock, H. M., O. F. AbouEzzeddine and M. M. Redfield	- 2018	High-sensitivity C-reactive protein in heart failure with preserved ejection fraction	irrelevent reports	PLoS One

Sokolski, M., K. Reszka, T. Suchocki, B. Adamik, A. Doroszko, J. Drobnik, J. Gorka-Dynysiewicz, M. Jedrzejczyk, K. Kaliszewski, K. Kilis-Pstrusinska, B. Konopska, A. Kopec, A. Larysz, W. Lis, A. Matera-Witkiewicz, L. Pawlik-Sobecka, M. Rosiek-Biegus, J. M. Sokolska, J. Sokolowski, A. Zapolska-Tomasiewicz, M. Protasiewicz, K. Madziarska and E. A. Jankowska	- 2022	History of Heart Failure in Patients Hospitalized Due to COVID-19: Relevant Factor of In-Hospital Complications and All-Cause Mortality up to Six Months	irrelevent reports	Journal of Clinical Medicine
Bauersachs, J., S. Störk, M. Kung, C. Waller, F. Fidler, C. Hoyer, S. Frantz, F. Weidemann, G. Ertl and C. E. Angermann	- 2007	HMG CoA reductase inhibition and left ventricular mass in hypertrophic cardiomyopathy: a randomized placebo-controlled pilot study	irrelevent reports	Eur J Clin Invest
Zhang, X. L., T. Y. Wang, Z. Chen, H. W. Wang, Y. Yin, L. Wang, Y. Wang, B. Xu and W. Xu	- 2022	HMGB1-Promoted Neutrophil Extracellular Traps Contribute to Cardiac Diastolic Dysfunction in Mice	irrelevent reports	J Am Heart Assoc
Schmidt, C., S. Magalhães, P. Gois Basilio, M. Gouveia, M. Teixeira, C. Santos, A. I. Tavares, J. P. Ferreira, F. Ribeiro and M. Santos	- 2024	Home- versus centre-based EXercise InTervention in patients with Heart Failure (EXIT-HF trial): A pragmatic randomized controlled trial	irrelevent reports	Revista Portuguesa de Cardiologia
Okuyan, E., A. Uslu, M. A. Çakar, I. Sahin, I. Önür, A. Enhos, H. I. Biter, S. Çetin and M. H. Dinçkal	- 2010	Homocysteine levels in patients with heart failure with preserved ejection fraction	irrelevent reports	Cardiology
Oyonarte Gómez, M., C. Del Castillo Gordillo, M. Rojas Romero and K. Loo Urbina	- 2021	Human bite wounds as a portal of entry for infective endocarditis and purulent pericarditis: A very rare association	irrelevent reports	BMJ Case Reports
Sato, Y., A. Yoshihisa, M. Oikawa, T. Nagai, T. Yoshikawa, Y. Saito, K. Yamamoto, Y. Takeishi and T. Anzai	- 2019	Hyponatremia at discharge is associated with adverse prognosis in acute heart failure syndromes with preserved ejection fraction: a report from the JASPER registry	irrelevent reports	European Heart Journal: Acute Cardiovascular Care
Moutinho, A., R. G. Cunha, S. K. Jamal, M. M. Lisboa and S. Tavares	- 2021	Idiopathic Multicentric Hyaline Vascular-Type Castleman Disease	irrelevent reports	Case Reports in Hematology
Van Tassell, B. W., C. R. Trankle, J. M. Canada, S. Carbone, L. Buckley, D. Kadariya, M. G. Del Buono, H. Billingsley, G. Wohlford, M. Viscusi, C. Oddi-Erdle, N. A. Abouzaki, D. Dixon, G. Biondi-Zoccai, R. Arena and A. Abbate	- 2018	IL-1 Blockade in Patients With Heart Failure With Preserved Ejection Fraction	irrelevent reports	Circ Heart Fail
Yu, Q., R. Vazquez, E. V. Khojeini, C. Patel, R. Venkataramani and D. F. Larson	- 2009	IL-18 induction of osteopontin mediates cardiac fibrosis and diastolic dysfunction in mice	irrelevent reports	Am J Physiol Heart Circ Physiol
Smart, C. D., D. J. Fehrenbach, J. W. Wassenaar, V. Agrawal, N. L. Fortune, D. D. Dixon, M. A. Cottam, A. H. Hasty, A. R. Hemnes, A. C. Doran, D. K. Gupta and M. S. Madhur	- 2023	Immune profiling of murine cardiac leukocytes identifies triggering receptor expressed on myeloid cells 2 as a novel mediator of hypertensive heart failure	irrelevent reports	Cardiovasc Res
Perticone, M., R. Zito, S. Miceli, A. Pinto, E. Suraci, M. Greco, S. Gigliotti, M. L. Hribal, S. Corrao, G. Sesti and F. Perticone	- 2019	Immunity, Inflammation and Heart Failure: Their Role on Cardiac Function and Iron Status	irrelevent reports	Frontiers in Immunology

Ratchford, S. M., H. L. Clifton, J. R. Gifford, D. T. LaSalle, T. S. Thurston, K. Bunsawat, J. K. Alpenglow, R. S. Richardson, J. B. Wright, J. J. Ryan and D. W. Wray	- 2019	Impact of acute antioxidant administration on inflammation and vascular function in heart failure with preserved ejection fraction	irrelevent reports	American Journal of Physiology - Regulatory Integrative and Comparative Physiology
Zakeri, R., B. A. Borlaug, S. E. McNulty, S. F. Mohammed, G. D. Lewis, M. J. Semigran, A. Deswal, M. LeWinter, A. F. Hernandez, E. Braunwald and M. M. Redfield	- 2014	Impact of atrial fibrillation on exercise capacity in heart failure with preserved ejection fraction a relax trial ancillary study	irrelevent reports	Circulation: Heart Failure
Mooney, L., N. M. Hawkins, P. S. Jhund, M. M. Redfield, M. Vaduganathan, A. S. Desai, J. L. Rouleau, M. Minamisawa, A. M. Shah, M. P. Lefkowitz, M. R. Zile, D. J. Van Veldhuisen, M. A. Pfeffer, I. S. Anand, A. P. Maggioni, M. Senni, B. L. Claggett, S. D. Solomon and J. J. V. McMurray	- 2021	Impact of Chronic Obstructive Pulmonary Disease in Patients With Heart Failure With Preserved Ejection Fraction: Insights From PARAGON-HF	irrelevent reports	J Am Heart Assoc
De Marco, C., B. L. Claggett, S. de Denus, M. R. Zile, T. Huynh, A. S. Desai, M. G. Sirois, S. D. Solomon, B. Pitt, J. L. Rouleau, M. A. Pfeffer and E. O'Meara	- 2021	Impact of diabetes on serum biomarkers in heart failure with preserved ejection fraction: insights from the TOPCAT trial	irrelevent reports	ESC Heart Failure
Pugliese, N. R., F. Paneni, M. Mazzola, N. De Biase, L. Del Punta, L. Gargani, A. Mengozzi, A. Virdis, L. Nesti, S. Taddei, A. Flammer, B. A. Borlaug, F. Ruschitzka and S. Masi	- 2021	Impact of epicardial adipose tissue on cardiovascular haemodynamics, metabolic profile, and prognosis in heart failure	irrelevent reports	European Journal of Heart Failure
Wohlfahrt, P., M. M. Redfield, F. Lopez-Jimenez, V. Melenovsky, G. C. Kane, R. J. Rodeheffer and B. A. Borlaug	- 2014	Impact of general and central adiposity onventricular-arterial aging in women and men	irrelevent reports	JACC: Heart Failure
Silverman, M. G., B. Patel, R. Blankstein, J. A. C. Lima, R. S. Blumenthal, K. Nasir and M. J. Blaha	- 2016	Impact of Race, Ethnicity, and Multimodality Biomarkers on the Incidence of New-Onset Heart Failure with Preserved Ejection Fraction (from the Multi-Ethnic Study of Atherosclerosis)	irrelevent reports	American Journal of Cardiology
Imai, R., Y. Uemura, T. Okumura, K. Takemoto, T. Uchikawa, M. Koyasu, S. Ishikawa, S. Iwamiya, Y. Ozaki, R. Shibata, M. Watarai and T. Murohara	- 2017	Impact of red blood cell distribution width on non-cardiac mortality in patients with acute decompensated heart failure with preserved ejection fraction	irrelevent reports	Journal of Cardiology
Yano, M., M. Nishino, S. Kawanami, H. Sugae, K. Ukita, A. Kawamura, K. Yasumoto, M. Tsuda, N. Okamoto, Y. Matsunaga-Lee, Y. Egami, J. Tanouchi, T. Yamada, Y. Yasumura, M. Seo, T. Hayashi, A. Nakagawa, Y. Nakagawa, S. Tamaki, Y. Sotomi, D. Nakatani, S. Hikoso and Y. Sakata	- 2023	Impact of Structural Abnormalities in Left Ventricle and Left Atrium on Clinical Outcomes in Heart Failure with Preserved Ejection Fraction	irrelevent reports	International Heart Journal
Hirose, S., S. Miyazaki, S. Yatsu, A. Sato, S. Ishiwata, H. Matsumoto, J. Shitara, A. Murata, T. Kato, S. Suda, Y. Matsue, M. Hiki, A. Takagi, H. Daida and T. Kasai	- 2020	Impact of the geriatric nutritional risk index on in-hospital mortality and length of hospitalization in patients with acute decompensated heart failure with preserved or reduced ejection fraction	irrelevent reports	Journal of Clinical Medicine
Caliskan, Z., H. S. Gokturk, M. Caliskan, H. Gullu, O. Ciftci, G. T. Ozgur, A. Guven and H. Selcuk	- 2015	Impaired coronary microvascular and left ventricular diastolic function in patients with inflammatory bowel disease	irrelevent reports	Microvasc Res
Hussain, I., S. F. Mohammed, P. R. Forfia, G. D. Lewis, B. A. Borlaug, D. S. Gallup and M. M. Redfield	- 2016	Impaired right ventricular-pulmonary arterial coupling and effect of sildenafil in heart failure with preserved ejection fraction	irrelevent reports	Circulation: Heart Failure

Smolgovsky, S., A. L. Bayer, K. Kaur, E. Sanders, M. Aronovitz, M. E. Filipp, E. B. Thorp, G. G. Schiattarella, J. A. Hill, R. M. Blanton, J. R. Cubillos-Ruiz and P. Alcaide	- 2023	Impaired T cell IRE1α/XBP1 signaling directs inflammation in experimental heart failure with preserved ejection fraction	irrelevent reports	J Clin Invest
Medranda, G. A., H. Fazlalizadeh, B. C. Case, C. Yerasi, C. Zhang, H. Rappaport, C. Shea, W. S. Weintraub and R. Waksman	- 2021	Implications of Left Ventricular Function on Short-Term Outcomes in COVID-19 Patients With Myocardial Injury	irrelevent reports	Cardiovascular Revascularization Medicine
Ishigami, J., L. T. Cowan, R. T. Demmer, M. E. Grams, P. L. Lutsey, J. J. Carrero, J. Coresh and K. Matsushita	- 2020	Incident hospitalization with major cardiovascular diseases and subsequent risk of ESKD: Implications for cardiorenal syndrome	irrelevent reports	Journal of the American Society of Nephrology
Rocha, J. A., S. P. Ribeiro, C. M. França, O. Coelho, G. Alves, S. Lacchini, E. G. Kallás, M. C. Irigoyen and F. M. Consolim-Colombo	- 2016	Increase in cholinergic modulation with pyridostigmine induces anti-inflammatory cell recruitment soon after acute myocardial infarction in rats	irrelevent reports	Am J Physiol Regul Integr Comp Physiol
Turak, O., F. Özcan, U. Canpolat, A. Işleyen, M. Cebeci, F. Öksüz, M. A. Mendi, K. Çağli, Z. Gölbaşi and S. Aydoğdu	- 2013	Increased echocardiographic epicardial fat thickness and high-sensitivity CRP level indicate diastolic dysfunction in patients with newly diagnosed essential hypertension	irrelevent reports	Blood Press Monit
Gotsman, I., A. Keren, O. Amir and D. R. Zwas	- 2022	Increased estimated fat-free mass and fat mass associated with improved clinical outcome in heart failure	irrelevent reports	European Journal of Clinical Investigation
Aoyama, N., K. Kure, M. Minabe and Y. Izumi	- 2019	Increased heart failure prevalence in patients with a high antibody level against periodontal pathogen	irrelevent reports	International Heart Journal
Schau, T., M. Gottwald, O. Arbach, M. Seifert, M. Schöpp, M. Neuß, C. Butter and M. Zänker	- 2015	Increased prevalence of diastolic heart failure in patients with rheumatoid arthritis correlates with active disease, but not with treatment type	irrelevent reports	Journal of Rheumatology
Matsubara, J., S. Sugiyama, T. Nozaki, E. Akiyama, Y. Matsuzawa, H. Kurokawa, H. Maeda, K. Fujisue, K. Sugamura, E. Yamamoto, K. Matsui, H. Jinnouchi and H. Ogawa	- 2014	Incremental prognostic significance of the elevated levels of pentraxin 3 in patients with heart failure with normal left ventricular ejection fraction	irrelevent reports	Journal of the American Heart Association
Lai, Y. H., C. C. Liu, J. Y. Kuo, T. C. Hung, Y. J. Wu, H. I. Yeh, B. E. Bulwer and C. L. Hung	- 2014	Independent effects of body fat and inflammatory markers on ventricular geometry, midwall function, and atrial remodeling	irrelevent reports	Clin Cardiol
Szelényi, Z., Á. Fazakas, G. Szénási, M. Kiss, N. Tegze, B. C. Fekete, E. Nagy, I. Bodó, B. Nagy, A. Molvarec, A. Patócs, L. Pepó, Z. Prohászka and A. Vereckei	- 2015	Inflammation and oxidative stress caused by nitric oxide synthase uncoupling might lead to left ventricular diastolic and systolic dysfunction in patients with hypertension	irrelevent reports	Journal of Geriatric Cardiology
López-Sánchez, M., M. Muñoz-Esquerre, D. Huertas, A. Montes, M. Molina-Molina, F. Manresa, J. Dorca and S. Santos	- 2017	Inflammatory markers and circulating extracellular matrix proteins in patients with chronic obstructive pulmonary disease and left ventricular diastolic dysfunction	irrelevent reports	Clin Respir J
McKechnie, D. G. J., A. O. Papacosta, L. T. Lennon, P. Welsh, P. H. Whincup and S. G. Wannamethee	- 2021	Inflammatory markers and incident heart failure in older men: The role of NT-proBNP	irrelevent reports	Biomarkers in Medicine
Kloch, M., K. Stolarz-Skrzypek, A. Olszanecka, W. Wojciechowska, A. Bednarski, J. Stefaniak and D. Czarnecka	- 2019	Inflammatory markers and left ventricular diastolic dysfunction in a family-based population study	irrelevent reports	Kardiol Pol
Albar, Z., M. Albakri, J. Hajjari, M. Karnib, S. E. Janus and S. G. Al-Kindi	- 2022	Inflammatory Markers and Risk of Heart Failure With Reduced to Preserved Ejection Fraction	irrelevent reports	American Journal of Cardiology

Masiha, S., J. Sundström and L. Lind	- 2013	Inflammatory markers are associated with left ventricular hypertrophy and diastolic dysfunction in a population-based sample of elderly men and women	irrelevent reports	J Hum Hypertens
Tian, L., X. Li, J. Zhang, X. Tian, X. Wan, D. Yao, B. Luo, Q. Huang, Y. Deng and W. Xiang	- 2024	Influence of Body Mass Index on the Prognostic Value of N-Terminal Pro-B-Type Natriuretic Peptide Level in Chinese Patients with Heart Failure	irrelevent reports	International Heart Journal
Rungwerth, K., U. Schindler, M. Gerl, S. Schäfer, T. Licher, A. E. Busch and H. Ruetten	- 2004	Inhibition of Na+-H+ exchange by cariporide reduces inflammation and heart failure in rabbits with myocardial infarction	irrelevent reports	Br J Pharmacol
Karabağ, Y., T. Çınar, M. Çağdaş, İ. Rencüzoğulları and V. O. Tanık	- 2019	In-hospital and long-term prognoses of patients with a mid-range ejection fraction after an ST-segment myocardial infarction	irrelevent reports	Acta Cardiologica
Wisniacki, N., W. Taylor, M. Lye and J. P. H. Wilding	- 2005	Insulin resistance and inflammatory activation in older patients with systolic and diastolic heart failure	irrelevent reports	Heart
Kosmala, W., M. Przewlocka-Kosmala, A. Wojnalowicz, A. Mysiak and T. H. Marwick	- 2012	Integrated backscatter as a fibrosis marker in the metabolic syndrome: association with biochemical evidence of fibrosis and left ventricular dysfunction	irrelevent reports	Eur Heart J Cardiovasc Imaging
Samoilova, E. V., A. Korotaeva, I. V. Zhirov, Y. O. Aksenova, S. N. Nasonova and S. N. Tereschenko	- 2024	Interleukin 6 Signalling in Heart Failure With Preserved and Reduced Ejection Fraction	irrelevent reports	Kardiologiia
Alogna, A., K. E. Koepp, M. Sabbah, J. M. Espindola Netto, M. D. Jensen, J. L. Kirkland, C. S. P. Lam, M. Obokata, M. C. Petrie, P. M. Ridker, H. Sorimachi, T. Tchkonia, A. Voors, M. M. Redfield and B. A. Borlaug	- 2023	Interleukin-6 in Patients With Heart Failure and Preserved Ejection Fraction	irrelevent reports	JACC: Heart Failure
Villalba-Cuesta, P. L., C. Álvaro-Vegue, C. G. Carrasco-Muñoz, C. Gomis-Goti and A. García-Villa	- 2022	Interstitial pneumonitis associated with leuprorelin acetate for a prostate cancer: A case report	irrelevent reports	Journal of Oncology Pharmacy Practice
Blatt, A., S. Minha, G. Moravsky, Z. Vered and R. Krakover	- 2010	Intracoronary administration of autologous bone marrow mononuclear cells in patients with chronic ischemic symptomatic cardiomyopathy: 5 years follow-up	irrelevent reports	Isr Med Assoc J
Kang, Z., Y. Wu, Y. Ding, Y. Zhang, X. Cai, H. Yang and J. Wei	- 2024	Investigation of the efficacy of Dengzhan Shengmai capsule against heart failure with preserved ejection fraction	irrelevent reports	Journal of Ethnopharmacology
Xia, Y. Y., Y. Shi, Z. Li, H. Li, L. D. Wu, W. Y. Zhou, Y. Gu, Z. Y. Ling, J. X. Zhang and S. L. Chen	- 2022	Involvement of pyroptosis pathway in epicardial adipose tissue - myocardium axis in experimental heart failure with preserved ejection fraction	irrelevent reports	Biochem Biophys Res Commun
Beale, A., D. Carballo, J. Stirnemann, N. Garin, T. Agoritsas, J. Serratrice, D. Kaye, P. Meyer and S. Carballo	- 2019	Iron deficiency in acute decompensated heart failure	irrelevent reports	Journal of Clinical Medicine
Bekfani, T., P. Pellicori, D. Morris, N. Ebner, M. Valentova, A. Sandek, W. Doehner, J. G. Cleland, M. Lainscak, P. C. Schulze, S. D. Anker and S. von Haehling	- 2019	Iron deficiency in patients with heart failure with preserved ejection fraction and its association with reduced exercise capacity, muscle strength and quality of life	irrelevent reports	Clinical Research in Cardiology
Alcaide-Aldeano, A., A. Garay, L. Alcoberro, S. Jiménez-Marrero, S. Yun, M. Tajes, E. García-Romero, C. Díez-López, J. González-Costello, G. Mateus-Porta, M. Cainzos-Achirica, C. Enjuanes, J. Comín-Colet and P. Moliner	- 2020	Iron deficiency: Impact on functional capacity and quality of life in heart failure with preserved ejection fraction	irrelevent reports	Journal of Clinical Medicine
Tao, J., H. Chen, Y. J. Wang, J. X. Qiu, Q. Q. Meng, R. J. Zou, L. Li, J. G. Huang, Z. K. Zhao, Y. L. Huang, H. F. Zhang and J. M. Zheng	- 2021	Ketogenic Diet Suppressed T-Regulatory Cells and Promoted Cardiac Fibrosis via Reducing Mitochondria-Associated Membranes and Inhibiting Mitochondrial Function	irrelevent reports	Oxid Med Cell Longev

Cho, J. Y., D. H. Cho, J. C. Youn, D. Kim, S. M. Park, M. H. Jung, J. Hyun, J. Choi, H. J. Cho, S. M. Park, J. O. Choi, W. J. Chung, B. S. Yoo and S. M. Kang	- 2023	Korean Society of Heart Failure Guidelines for the Management of Heart Failure: Definition and Diagnosis	irrelevent reports	Korean Circulation Journal
Degousee, N., J. Simpson, S. Fazel, K. Scholich, D. Angoulvant, C. Angioni, H. Schmidt, M. Korotkova, E. Stefanski, X. H. Wang, T. F. Lindsay, E. Ofek, S. Pierre, J. Butany, P. J. Jakobsson, A. Keating, R. K. Li, M. Nahrendorf, G. Geisslinger, P. H. Backx and B. B. Rubin	- 2012	Lack of microsomal prostaglandin E(2) synthase-1 in bone marrow-derived myeloid cells impairs left ventricular function and increases mortality after acute myocardial infarction	irrelevent reports	Circulation
Liu, Y. X., H. Li, Y. Y. Xia, C. L. Xia, X. L. Qu, P. Chu, W. Y. Zhou, L. L. Zhu, L. Li, S. L. Chen and J. X. Zhang	- 2020	Left atrial diameter and atrial fibrillation, but not elevated NT-proBNP, predict the development of pulmonary hypertension in patients with HFpEF	irrelevent reports	Journal of Geriatric Cardiology
Kizer, J. R., J. N. Bella, V. Palmieri, J. E. Liu, L. G. Best, E. T. Lee, M. J. Roman and R. B. Devereux	- 2006	Left atrial diameter as an independent predictor of first clinical cardiovascular events in middle-aged and elderly adults: the Strong Heart Study (SHS)	irrelevent reports	Am Heart J
Maffeis, C., D. A. Morris, E. Belyavskiy, M. Kropf, A. K. Radhakrishnan, V. Zach, C. Rozados da Conceicao, T. D. Trippel, E. Pieske-Kraigher, A. Rossi, B. Pieske and F. Edelmann	- 2021	Left atrial function and maximal exercise capacity in heart failure with preserved and mid-range ejection fraction	irrelevent reports	ESC Heart Failure
Brener, M. I. and S. Ravalli	- 2020	Left Ventricular Cavity Obliteration From Eosinophilic Myocarditis in a Patient With Classic Hodgkin Lymphoma	irrelevent reports	JACC: Case Reports
Romejko, K., A. Rymarz, K. Szamotulska, Z. Bartoszewicz, T. Rozmyslowicz and S. Niemczyk	- 2022	Left Ventricular Diastolic Dysfunction in Chronic Kidney Disease Patients Not Treated with Dialysis	irrelevent reports	Nutrients
Kosmala, W., D. Jedrzejuk, R. Derzhko, M. Przewlocka-Kosmala, A. Mysiak and G. Bednarek-Tupikowska	- 2012	Left ventricular function impairment in patients with normal-weight obesity: contribution of abdominal fat deposition, profibrotic state, reduced insulin sensitivity, and proinflammatory activation	irrelevent reports	Circ Cardiovasc Imaging
Ghionzoli, N., A. Gismondi, G. E. Mandoli, L. Spera, A. Di Florio, F. D'Ascenzi, M. Cameli, L. Cavigli, C. Sciaccaluga, S. F. Carbone, G. D. Aquaro, S. Valente and M. Focardi	- 2022	Left Ventricular Mass with Delayed Enhancement as a Predictor of Major Events in Patients with Myocarditis with Preserved Ejection Fraction	irrelevent reports	Journal of Clinical Medicine
Beitnes, J. O., O. Gjesdal, K. Lunde, S. Solheim, T. Edvardsen, H. Arnesen, K. Forfang and S. Aakhus	- 2011	Left ventricular systolic and diastolic function improve after acute myocardial infarction treated with acute percutaneous coronary intervention, but are not influenced by intracoronary injection of autologous mononuclear bone marrow cells: a 3 year serial echocardiographic sub-study of the randomized-controlled ASTAMI study	irrelevent reports	Eur J Echocardiogr
Nevers, T., A. M. Salvador, A. Grodecki-Pena, A. Knapp, F. Velázquez, M. Aronovitz, N. K. Kapur, R. H. Karas, R. M. Blanton and P. Alcaide	- 2015	Left Ventricular T-Cell Recruitment Contributes to the Pathogenesis of Heart Failure	irrelevent reports	Circ Heart Fail
Wilson, I. C., J. M. DiNatale, A. M. Gillinov, W. E. Curtis, D. E. Cameron and T. J. Gardner	- 1993	Leukocyte depletion in a neonatal model of cardiac surgery	irrelevent reports	Ann Thorac Surg
Wang, J. W., M. S. C. Fontes, X. Wang, S. Y. Chong, E. L. Kessler, Y. N. Zhang, J. J. de Haan, F. Arslan, S. C. A. de Jager, L. Timmers, T. A. B. van Veen, C. S. P. Lam and D. P. V. Kleijn	- 2017	Leukocytic Toll-Like Receptor 2 Deficiency Preserves Cardiac Function And Reduces Fibrosis In Sustained Pressure Overload	irrelevent reports	Sci Rep

Li, M. M., W. C. Shen, Y. J. Li and J. Teng	- 2022	Linezolid-Induced Pancytopenia in Patients Using Dapagliflozin: A Case Series	irrelevent reports	Infection and Drug Resistance
Chalubinska-Fendler, J., L. Graczyk, G. Piotrowski, K. Wyka, Z. Nowicka, B. Tomasik, J. Fijuth, D. Kozono and W. Fendler	- 2019	Lipopolysaccharide-Binding Protein Is an Early Biomarker of Cardiac Function After Radiation Therapy for Breast Cancer	irrelevent reports	Int J Radiat Oncol Biol Phys
Jun-Feng, C., J. Xiao-Ping, Z. Juan, Y. Man-Li, L. Fan, F. Peng, W. Qi-Shun, S. Yang, S. Bo-Lin, H. Yu-Wen and C. Liang	- 2023	Lipoprotein-Associated Phospholipase A2Correlates with Reduced Left Ventricle Ejection Fraction in Hemodialysis Patients	irrelevent reports	Lab Medicine
Hiramatsu, T., Y. Asano, M. Mabuchi, K. Imai, D. Iguchi and S. Furuta	- 2018	Liraglutide relieves cardiac dilated function than DPP-4 inhibitors	irrelevent reports	Eur J Clin Invest
Francisco, M. A., J. F. Lee, Z. Barrett-O'keefe, H. J. Groot, S. M. Ratchford, K. Bunsawat, J. K. Alpenglow, J. J. Ryan, J. N. Nativi, R. S. Richardson and D. W. Wray	- 2021	Locomotor Muscle Microvascular Dysfunction in Heart Failure With Preserved Ejection Fraction	irrelevent reports	Hypertension
Alam, A., S. Thampi, S. G. Saba and R. Jermyn	- 2017	Loeffler Endocarditis: A Unique Presentation of Right-Sided Heart Failure Due to Eosinophil-Induced Endomyocardial Fibrosis	irrelevent reports	Clinical Medicine Insights: Case Reports
Kontaraki, J. E., M. E. Marketou, G. E. Kochiadakis, A. Patrianakos, S. Maragkoudakis, A. Plevritaki, S. Papadaki, A. Alevizaki, O. Theodosaki and F. I. Parthenakis	- 2021	Long noncoding RNAs in peripheral blood mononuclear cells of hypertensive patients with heart failure with preserved ejection fraction in relation to their functional capacity	irrelevent reports	Hellenic J Cardiol
Klimczak-Tomaniak, D., V. J. van den Berg, M. Strachinaru, K. M. Akkerhuis, S. Baart, K. Caliskan, O. C. Manintveld, V. Umans, M. Geleijnse, E. Boersma, B. M. van Dalen and I. Kardys	- 2020	Longitudinal patterns of N-terminal pro B-type natriuretic peptide, troponin T, and C-reactive protein in relation to the dynamics of echocardiographic parameters in heart failure patients	irrelevent reports	Eur Heart J Cardiovasc Imaging
Meyer, T. E., S. J. Kovács, A. A. Ehsani, S. Klein, J. O. Holloszy and L. Fontana	- 2006	Long-term caloric restriction ameliorates the decline in diastolic function in humans	irrelevent reports	J Am Coll Cardiol
Zhang, S., J. Guo, P. Zhang, Y. Liu, Z. Jia, K. Ma, W. Li, L. Li and C. Zhou	- 2004	Long-term effects of bone marrow mononuclear cell transplantation on left ventricular function and remodeling in rats	irrelevent reports	Life Sci
Klobučar, I., L. Vidović, I. Arih, M. Lechleitner, G. Pregartner, A. Berghold, H. Habisch, T. Madl, S. Frank and V. Degoricija	- 2023	Low Valine Serum Levels Predict Increased 1-Year Mortality in Acute Heart Failure Patients	irrelevent reports	Biomolecules
Ma, Z., L. Zhao, S. Martin, Y. Zhang, Y. Dong, J. C. Zhong and X. C. Yang	- 2021	Lower Plasma Elabela Levels in Hypertensive Patients With Heart Failure Predict the Occurrence of Major Adverse Cardiac Events: A Preliminary Study	irrelevent reports	Frontiers in Cardiovascular Medicine
Agibetov, A., B. Seirer, T. M. Dachs, M. Koschutnik, D. Dalos, R. Rettl, F. Duca, L. Schrutka, H. Agis, R. Kain, M. Auer-Grumbach, C. Binder, J. Mascherbauer, C. Hengstenberg, M. Samwald, G. Dorffner and D. Bonderman	- 2020	Machine learning enables prediction of cardiac amyloidosis by routine laboratory parameters: A proof-of-concept study	irrelevent reports	Journal of Clinical Medicine
Chien, S. C., C. I. Lo, C. F. Lin, K. T. Sung, J. P. Tsai, W. H. Huang, C. H. Yun, T. C. Hung, J. L. Lin, C. Y. Liu, C. J. Y. Hou, I. H. Tsai, C. H. Su, H. I. Yeh and C. L. Hung	- 2019	Malnutrition in acute heart failure with preserved ejection fraction: clinical correlates and prognostic implications	irrelevent reports	ESC Heart Failure
Watanabe, Y., Y. Horiuchi, M. Nakase, N. Setoguchi, T. Ishizawa, M. Sekiguchi, H. Nonaka, M. Nakajima, M. Asami, K. Yahagi, K. Komiyama, H. Yuzawa, J. Tanaka, J. Aoki and K. Tanabe	- 2022	Malnutrition, hemodynamics and inflammation in heart failure with reduced, mildly reduced and preserved ejection fraction	irrelevent reports	Heart Vessels

Currier, J. S.	- 2018	Management of long-term complications of HIV disease: Focus on cardiovascular disease	irrelevent reports	Topics in Antiviral Medicine
Rupawala, A. H., D. Gachette, M. Bakhit, L. Jimoh and C. R. Kelly	- 2021	Management of Severe and Severe/Complicated Clostridoides difficile Infection Using Sequential Fecal Microbiota Transplant by Retention Enema	irrelevent reports	Clinical Infectious Diseases
Sciarretta, S., A. Ferrucci, G. M. Ciavarella, P. De Paolis, V. Venturelli, G. Tocci, L. De Biase, S. Rubattu and M. Volpe	- 2007	Markers of inflammation and fibrosis are related to cardiovascular damage in hypertensive patients with metabolic syndrome	irrelevent reports	Am J Hypertens
Van de Veire, N. R., O. De Winter, J. Philippé, M. De Buyzere, D. Bernard, M. Langlois, T. C. Gillebert and J. De Sutter	- 2006	Maximum oxygen uptake at peak exercise in elderly patients with coronary artery disease and preserved left ventricular function: the role of inflammation on top of tissue Doppler-derived systolic and diastolic function	irrelevent reports	Am Heart J
Lüscher, T. F.	- 2018	Mechanisms and outcomes of heart failure: From HFpEF, HFmrEF, and HFrEF to transplantation	irrelevent reports	European Heart Journal
Myhre, P. L., M. Vaduganathan, E. O'Meara, B. L. Claggett, S. De Denus, P. Jarolim, I. S. Anand, B. Pitt, J. L. Rouleau, S. D. Solomon, M. A. Pfeffer and A. S. Desai	- 2020	Mechanistic Effects of Spironolactone on Cardiovascular and Renal Biomarkers in Heart Failure with Preserved Ejection Fraction: A TOPCAT Biorepository Study	irrelevent reports	Circulation: Heart Failure
Kouvari, M., C. Chrysohoou, P. Aggelopoulos, E. Tsiamis, K. Tsioufis, C. Pitsavos and D. Tousoulis	- 2017	Mediterranean diet and prognosis of first-diagnosed Acute Coronary Syndrome patients according to heart failure phenotype: Hellenic Heart Failure Study	irrelevent reports	European Journal of Clinical Nutrition
Marketou, M. E., J. E. Kontaraki, S. Maragkoudakis, A. Patrianakos, J. Konstantinou, H. Nakou, D. Vougia, J. Logakis, G. Chlouverakis, P. E. Vardas and F. I. Parthenakis	- 2018	MicroRNAs in Peripheral Mononuclear Cells as Potential Biomarkers in Hypertensive Patients With Heart Failure With Preserved Ejection Fraction	irrelevent reports	Am J Hypertens
Cohen, A. J., K. Teramoto, B. Claggett, L. E. O. Buckley, S. Solomon, C. Ballantyne, E. Selvin and A. M. Shah	- 2021	Mid- to Late-Life Inflammation and Risk of Cardiac Dysfunction, HFpEF and HFrEF in Late Life	irrelevent reports	Journal of Cardiac Failure
Sotomi, Y., T. Sato, S. Hikoso, S. Komukai, B. Oeun, T. Kitamura, D. Nakatani, H. Mizuno, K. Okada, T. Dohi, A. Sunaga, H. Kida, M. Seo, M. Yano, T. Hayashi, A. Nakagawa, Y. Nakagawa, S. Tamaki, T. Ohtani, Y. Yasumura, T. Yamada and Y. Sakata	- 2022	Minimal subphenotyping model for acute heart failure with preserved ejection fraction	irrelevent reports	ESC Heart Failure
Buckley, L. F., A. M. Agha, P. Dorbala, B. L. Claggett, B. Yu, A. Hussain, V. Nambi, L. Y. Chen, K. Matsushita, R. C. Hoogeveen, C. M. Ballantyne and A. M. Shah	- 2023	MMP-2 Associates With Incident Heart Failure and Atrial Fibrillation: The ARIC Study	irrelevent reports	Circulation: Heart Failure
Bolat, I. and M. Biteker	- 2020	Modified Glasgow Prognostic Score is a novel predictor of clinical outcome in heart failure with preserved ejection fraction	irrelevent reports	Scand Cardiovasc J
Lebedev, D. A., E. A. Lyasnikova, A. A. Vasilyeva, E. Y. Vasilyeva, A. Y. Babenko and E. V. Shlyakhto	- 2020	Molecular biomarker profile of heart failure with mid-range and preserved ejection fraction in patients with type 2 diabetes	irrelevent reports	Russian Journal of Cardiology
Bozkurt, D., S. M. K. Bozgul, O. Emgin, O. Butun, T. Kose, E. Simsek, M. Hekimgil and S. Kilic	- 2021	Mortal Interaction Between Hemophagocytic Syndrome and Newly Developed Heart Failure	irrelevent reports	Arq Bras Cardiol

Bozkurt, D., S. M. K. Bozgul, O. Emgin, O. Butun, T. Kose, E. Simsek, M. Hekimgil and S. Kilic	- 2021	Mortal İnteraction between hemophagocytic syndrome and newly developed heart fail	irrelevent reports	Arquivos Brasileiros de Cardiologia
He, G., L. Zhang, X. Huo, Q. Wang, D. Hu, X. Huang, J. Ge, Y. Wu and J. Li	- 2022	Multi-Biomarker Points and Outcomes in Patients Hospitalized for Heart Failure: Insights From the China PEACE Prospective Heart Failure Study	irrelevent reports	Frontiers in Cardiovascular Medicine
Večerić-Haler, Ž., Š. Borštnar, B. Luzar, M. Jerše and N. Kojc	- 2021	Multiorgan failure with fatal outcome after stem cell tourism	irrelevent reports	European Journal of Medical Research
Chugh, S., A. Kichloo, F. Jafri, L. Yusvirazi and R. Lerner	- 2017	Multiple Myeloma as the Underlying Cause of Thrombotic Microangiopathy Leading to Acute Kidney Injury: Revisiting a Very Rare Entity	irrelevent reports	Journal of Investigative Medicine High Impact Case Reports
Zile, M. R., C. F. Baicu, J. S. Ikonomidis, R. E. Stroud, P. J. Nietert, A. D. Bradshaw, R. Slater, B. M. Palmer, P. Van Buren, M. Meyer, M. M. Redfield, D. A. Bull, H. L. Granzier and M. M. LeWinter	- 2015	Myocardial stiffness in patients with heart failure and a preserved ejection fraction contributions of collagen and titin	irrelevent reports	Circulation
Pais, I. P., A. Teles, N. Miranda and R. Pinto	- 2012	Necrotising enterocolitis in a full-term infant with reversed diastolic flow in the descending aorta: What is the diagnosis?	irrelevent reports	BMJ Case Reports
Riascos-Bernal, D. F. and N. E. Sibinga	- 2022	Neutrophil extracellular traps in cardiac hypertrophy: a KLF2 perspective	irrelevent reports	J Clin Invest
Van Bruggen, S., S. Kraisin, J. Van Wauwe, K. Bomhals, M. Stroobants, P. Carai, L. Frederix, A. Van De Bruaene, T. Witsch and K. Martinod	- 2023	Neutrophil peptidylarginine deiminase 4 is essential for detrimental age-related cardiac remodelling and dysfunction in mice	irrelevent reports	Philos Trans R Soc Lond B Biol Sci
Pecherina, T. B., V. N. Karetnikova, V. V. Kashtalap, E. V. Dren, J. S. Ignatova, S. Y. Shuster, A. V. Yurkina, Y. I. Gusel'nikova and O. L. Barbarash	- 2023	NEW BIOLOGICAL MARKERS FOR A PROGNOSTIC MODEL FOR ASSESSING THE RISK OF CARDIAC FIBROSIS IN PATIENTS WITH ST-SEGMENT ELEVATION MYOCARDIAL INFARCTION	irrelevent reports	Complex Issues of Cardiovascular Diseases
Chaulagain, C. P. and R. L. Comenzo	- 2013	New insights and modern treatment of AL amyloidosis	irrelevent reports	Current Hematologic Malignancy Reports
Piatek, K., A. Feuerstein, V. Zach, C. Rozados da Conceicao, A. Beblo, E. Belyavskiy, E. Pieske-Kraigher, A. Krannich, E. Schwedhelm, S. Hinz, B. Pieske and F. Edelmann	- 2022	Nitric oxide metabolites: associations with cardiovascular biomarkers and clinical parameters in patients with HFpEF	irrelevent reports	ESC Heart Fail
Borgognone, A., E. Shantsila, S. M. Worrall, E. Prompunt, T. Loka, B. L. Loudon, M. Chimen, G. E. Rainger, J. M. Lord, A. Turner, P. Nightingale, M. Feelisch, P. Kirchhof, G. Y. H. Lip, S. P. Watson, M. P. Frenneaux and M. Madhani	- 2018	Nitrite circumvents platelet resistance to nitric oxide in patients with heart failure preserved ejection fraction and chronic atrial fibrillation	irrelevent reports	Cardiovascular Research
Ebong, I. A., M. D. Wilson, P. Chang, D. Appiah, T. Polonsky, C. Ballantyne and A. G. Bertoni	- 2022	NT-pro B-type natriuretic peptide, early menopause, and incident heart failure in postmenopausal women of the ARIC study	irrelevent reports	Menopause
Gehle, P., P. N. Robinson, F. Heinzel, F. Edelmann, M. Yigitbasi, F. Berger, V. Falk, B. Pieske and E. Wellnhofer	- 2016	NT-proBNP and diastolic left ventricular function in patients with Marfan syndrome	irrelevent reports	IJC Heart and Vasculature
Sabbah, M. S., A. U. Fayyaz, S. De Denus, G. M. Felker, B. A. Borlaug, S. Dasari, R. E. Carter and M. M. Redfield	- 2020	Obese-Inflammatory Phenotypes in Heart Failure With Preserved Ejection Fraction	irrelevent reports	Circulation: Heart Failure
Ramirez, M. F., E. S. Lau, J. K. Parekh, A. S. Pan, N. Owunna, D. Wang, J. N. McNeill, R. Malhotra, M. Nayor, G. D. Lewis and J. E. Ho	- 2023	Obesity-Related Biomarkers Are Associated With Exercise Intolerance and HFpEF	irrelevent reports	Circulation: Heart Failure

Azarfar, A., A. Sharma, J. G. Parikh, N. M. Shaikh and K. King-Morris	- 2021	Ocular manifestation of giant cell arteritis vs AL-amyloidosis: similar presentations but different approaches	irrelevent reports	Modern Rheumatology Case Reports
Futai, R., T. Ito, Y. Kawanishi, F. Terasaki and Y. Kitaura	- 2009	Olmesartan ameliorates myocardial function independent of blood pressure control in patients with mild-to-moderate hypertension	irrelevent reports	Heart Vessels
Denil, S. L., E. R. Rietzschel, M. L. De Buyzere, C. M. Van Daele, P. Segers, D. De Bacquer, W. Van Criekinge, S. Bekaert, T. C. Gillebert and T. De Meyer	- 2014	On cross-sectional associations of leukocyte telomere length with cardiac systolic, diastolic and vascular function: the Asklepios study	irrelevent reports	PLoS One
Bezgin, T., A. Elveran, A. Karagoz, Y. Canga and C. Dogan	- 2016	Parathyroid hormone is associated with heart failure with preserved ejection fraction	irrelevent reports	Bratisl Lek Listy
Sotomi, Y., S. Tamaki, S. Hikoso, D. Nakatani, K. Okada, T. Dohi, A. Sunaga, H. Kida, T. Sato, Y. Matsuoka, D. Sakamoto, T. Kitamura, S. Komukai, M. Seo, M. Yano, T. Hayashi, A. Nakagawa, Y. Nakagawa, T. Ohtani, Y. Yasumura, T. Yamada and Y. Sakata	- 2023	Pathophysiological insights into machine learning-based subphenotypes of acute heart failure with preserved ejection fraction	irrelevent reports	Heart
Berezin, A. E., A. A. Kremzer, Y. V. Martovitskaya, T. A. Berezina and E. A. Gromenko	- 2016	Pattern of endothelial progenitor cells and apoptotic endothelial cell-derived microparticles in chronic heart failure patients with preserved and reduced left ventricular ejection fraction	irrelevent reports	EBioMedicine
Liu, Q., T. Tu, Z. Bai and S. Zhou	- 2011	Pentraxin 3 as a new biomarker of diastolic dysfunction	irrelevent reports	Int J Cardiol
Matsubara, J., S. Sugiyama, T. Nozaki, K. Sugamura, M. Konishi, K. Ohba, Y. Matsuzawa, E. Akiyama, E. Yamamoto, K. Sakamoto, Y. Nagayoshi, K. Kaikita, H. Sumida, S. Kim-Mitsuyama and H. Ogawa	- 2011	Pentraxin 3 is a new inflammatory marker correlated with left ventricular diastolic dysfunction and heart failure with normal ejection fraction	irrelevent reports	J Am Coll Cardiol
Somuncu, M., F. Tatar, N. Serbest, B. Uygur and A. Demir	- 2021	Pentraxin-3 is associated with adverse diastolic remodeling in patients with st-elevation myocardial infarction after successful reperfusion by primary percutaneous intervention	irrelevent reports	Journal of Cardiovascular Echography
Molinsky, R. L., M. Yuzefpolskaya, F. L. Norby, B. Yu, A. M. Shah, J. S. Pankow, C. E. Ndumele, P. L. Lutsey, P. N. Papapanou, J. D. Beck, P. C. Colombo and R. T. Demmer	- 2022	Periodontal Status, C-Reactive Protein, NT-proBNP, and Incident Heart Failure: The ARIC Study	irrelevent reports	JACC: Heart Failure
Molinsky, R. L., M. Yuzefpolskaya, F. L. Norby, B. Yu, A. M. Shah, J. S. Pankow, C. E. Ndumele, P. L. Lutsey, P. N. Papapanou, J. D. Beck, P. C. Colombo and R. T. Demmer	- 2022	Periodontal Status, C-Reactive Protein, NT-proBNP, and Incident Heart Failure: The ARIC Study	irrelevent reports	JACC Heart Fail
Marketou, M., J. Kontaraki, E. Zacharis, S. Maragkoudakis, K. Fragkiadakis, E. Kampanieris, A. Plevritaki, E. Savva, O. Malikides, G. Chlouverakis and G. Kochiadakis	- 2024	Peripheral Blood MicroRNA-21 as a Predictive Biomarker for Heart Failure With Preserved Ejection Fraction in Old Hypertensives	irrelevent reports	Am J Hypertens

Sotomi, Y., S. Hikoso, S. Komukai, T. Sato, B. Oeun, T. Kitamura, A. Nakagawa, D. Nakatani, H. Mizuno, K. Okada, T. Dohi, A. Sunaga, H. Kida, M. Seo, M. Yano, T. Hayashi, Y. Nakagawa, S. Tamaki, T. Ohtani, Y. Yasumura, T. Yamada and Y. Sakata	- 2022	Phenotyping of acute decompensated heart failure with preserved ejection fraction	irrelevent reports	Heart
Potratz, M., C. Sohns, D. Dumitrescu, P. Sommer and H. Fox	- 2021	Phrenic nerve stimulation improves physical performance and hypoxemia in heart failure patients with central sleep apnea	irrelevent reports	Journal of Clinical Medicine
Yi, L., A. Chuan, A. Xin, Z. Xinyu, S. Lin and Z. Quanlin	- 2024	Piperacillin-tazobactam-induced myocardial injury with heart failure: A case report	irrelevent reports	Experimental and Therapeutic Medicine
Guo, Y., N. Wang, Y. Dong, X. Li, Q. Liu, Q. Liu, G. Wang, M. Qin, Z. Zhang, J. Song, Y. Liu, H. Chi and J. Zhong	- 2023	Plasma levels of bone morphogenic protein-4 are downregulated in elderly hypertensive patients with heart failure with preserved ejection fraction	irrelevent reports	Clinical Biochemistry
Dixit, G., J. Blair and C. Ozcan	- 2022	Plasma proteomic analysis of association between atrial fibrillation, coronary microvascular disease and heart failure	irrelevent reports	American Journal of Cardiovascular Disease
Van Ommen, A. M., E. Diez Benavente, N. C. Onland-Moret, G. B. Valstar, M. J. Cramer, F. H. Rutten, A. J. Teske, R. Menken, L. Hofstra, I. I. Tulevski, N. Sweitzer, G. A. Somsen and H. M. Den Ruijter	- 2023	Plasma Proteomic Patterns Show Sex Differences in Early Concentric Left Ventricular Remodeling	irrelevent reports	Circulation: Heart Failure
Kanagala, P., J. R. Arnold, J. N. Khan, A. Singh, G. S. Gulsin, D. C. S. Chan, A. S. H. Cheng, J. Yang, Z. Li, P. Gupta, I. B. Squire, G. P. McCann and L. L. Ng	- 2020	Plasma Tenascin-C: a prognostic biomarker in heart failure with preserved ejection fraction	irrelevent reports	Biomarkers
Kuryata, O., O. Sirenko, A. Tykhomyrov and T. Yatsenko	- 2022	Plasminogen activator inhibitor-1 and circulating ceruloplasmin levels in men with iron-deficiency anemia and heart failure with concomitant prostate cancer and their dynamics after treatment	irrelevent reports	Journal of Medical Sciences (Taiwan)
Awad, A., S. Elnemr, H. Hodeib and D. El Amrousy	- 2022	Platelet Activation Markers in Children with Pulmonary Arterial Hypertension Associated with Congenital Heart Disease	irrelevent reports	Pediatr Cardiol
Shen, Q., S. A. Cintron and J. D. Pierce	- 2024	Platelet and Leukocyte Mitochondrial Function With Cardiac Function and Self-Reported Health Status Among Obese Patients With Heart Failure	irrelevent reports	Nurs Res
Mir, T. H., P. A. Zargar, A. Sharma, B. Jabeen, S. Sharma, M. O. Parvaiz, S. Bashir and R. Javeed	- 2023	Post COVID-19 AA amyloidosis of the kidneys with rapidly progressive renal failure	irrelevent reports	Prion
Ye, P., C. E. Wu, L. Sheng and H. Li	- 2009	Potential protective effect of long-term therapy with Xuezhikang on left ventricular diastolic function in patients with essential hypertension	irrelevent reports	J Altern Complement Med
Pugliese, N. R., N. De Biase, L. Gargani, M. Mazzola, L. Conte, I. Fabiani, A. Natali, F. L. Dini, P. Frumento, J. Rosada, S. Taddei, B. A. Borlaug and S. Masi	- 2021	Predicting the transition to and progression of heart failure with preserved ejection fraction: A weighted risk score using bio-humoural, cardiopulmonary, and echocardiographic stress testing	irrelevent reports	European Journal of Preventive Cardiology
Melbye, H., M. Stylidis, J. C. A. Solis, M. Averina and H. Schirmer	- 2020	Prediction of chronic heart failure and chronic obstructive pulmonary disease in a general population: the Tromsø study	irrelevent reports	ESC Heart Failure
Zacharoulis, A., V. Kotseroglou, S. Lerakis, A. Karavidas, S. Arapi and A. Zacharoulis	- 2006	Predictive value of C-reactive protein and left ventricular diastolic filling pattern after a non-ST elevation myocardial infarction	irrelevent reports	Am J Med Sci
Michowitz, Y., Y. Arbel, D. Wexler, D. Sheps, O. Rogowski, I. Shapira, S. Berliner, G. Keren, J. George and A. Roth	- 2008	Predictive value of high sensitivity CRP in patients with diastolic heart failure	irrelevent reports	Int J Cardiol
Zhao, L., X. Zhao, P. Tian, L. Liang, B. Huang, L. Huang, J. Feng, Y. Zhang and J. Zhang	- 2023	Predictive value of remnant cholesterol level for all-cause mortality in heart failure patients	irrelevent reports	Frontiers in Cardiovascular Medicine
Turen, S. and K. M. Sancar	- 2023	Predictive Value of the Prognostic Nutritional Index for Long-Term Mortality in Patients with Advanced Heart Failure	irrelevent reports	Acta Cardiologica Sinica

Chen, L., Z. Huang, X. Zhao, J. Liang, X. Lu, Y. He, Y. Kang, Y. Xie, J. Liu, Y. Liu, J. Yang, W. Yu, W. Deng, Y. Pan, J. Lu, Y. Yang, X. Xie, X. Qian, Q. Xu, L. Chen, K. Chen and S. Chen	- 2022	Predictors and Mortality for Worsening Left Ventricular Ejection Fraction in Patients With HFpEF	irrelevent reports	Frontiers in Cardiovascular Medicine
Nakagawa, A., Y. Yasumura, C. Yoshida, T. Okumura, J. Tateishi, J. Yoshida, M. Seo, M. Yano, T. Hayashi, Y. Nakagawa, S. Tamaki, T. Yamada, H. Kurakami, Y. Sotomi, D. Nakatani, S. Hikoso, Y. Sakata, Y. Sakata, S. Hikoso, D. Nakatani, H. Mizuno, K. Okada, T. Dohi, Y. Sotomi, A. Sunaga, H. Kida, B. Oeun, T. Sato, M. Seo, T. Watanabe, T. Yamada, T. Hayashi, Y. Higuchi, M. Masuda, M. Asai, T. Mano, H. Fuji, D. Masuda, S. Tamaki, R. Shutta, S. Yamashita, M. Sairyo, Y. Nakagawa, H. Abe, Y. Ueda, Y. Matsumura, K. Nagai, M. Yano, M. Nishino, J. Tanouchi, Y. Arita, N. Ogasawara, T. Ishizu, M. Ichikawa, Y. Takano, E. Rin, Y. Shinoda, K. Tachibana, S. Hoshida, M. Izumi, H. Yamamoto, H. Kato, K. Nakatani, Y. Yasuga, M. Nishio, K. Hirooka, T. Yoshimura, A. Tani, Y. Okumoto, Y. Makino, T. Onishi, K. Iwakura, Y. Kijima, T. Kitao, H. Kanai, M. Fujita, K. Harada, M. Kumada, O. Nakagawa, T. Yamada, A. Nakagawa, Y. Yasumura, T. Sato, A. Sunaga, B. Oeun, H. Kida, Y. Sotomi, T. Dohi, K. Nakamoto, K. Okada, F. Sera, H. Kioka, T. Ohtani, T. Takeda, D. Nakatani, H. Mizuno and S. Hikoso	- 2022	Predictors and Outcomes of Heart Failure With Preserved Ejection Fraction in Patients With a Left Ventricular Ejection Fraction Above or Below 60%	irrelevent reports	Journal of the American Heart Association
Rossi, V. A., I. Krizanovic-Grgic, J. Steffel, D. Hofer, T. Wolber, C. B. Brunckhorst, F. Ruschitzka, F. Duru, A. Breitenstein and A. M. Saguner	- 2022	Predictors of left atrial fibrosis in patients witatrial fibrillation referred for catheter ablatio	irrelevent reports	Cardiology Journal
Fujimoto, Y., D. Maeda, N. Kagiyama, T. Sunayama, T. Dotare, K. Jujo, K. Saito, K. Kamiya, H. Saito, Y. Ogasahara, E. Maekawa, M. Konishi, T. Kitai, K. Iwata, H. Wada, M. Hiki, T. Kasai, H. Nagamatsu, T. Ozawa, K. Izawa, S. Yamamoto, N. Aizawa, K. Wakaume, K. Oka, S. I. Momomura and Y. Matsue	- 2023	Prevalence and prognostic impact of the coexistence of cachexia and sarcopenia in older patients with heart failure	irrelevent reports	International Journal of Cardiology
Mini, P., B. Marc, S. Fabien and M. Chuzeville	- 2018	Prevalence of iron deficiency in patients aged 75 years or older with heart failure	irrelevent reports	Journal of Geriatric Cardiology

Caliskan, M., Y. Turan, Z. Caliskan, H. Gullu, F. C. Ciftci, E. Avci, C. Duran, O. Kostek, O. Telci Caklili, H. Koca and M. Kulaksizoglu	- 2015	Previous gestational diabetes history is associated with impaired coronary flow reserve	irrelevent reports	Ann Med
Ahmad, T. Y., H. N. Al Houri, S. Jomaa, W. Assad and S. Z. Addeen	- 2022	Primary splenic T-cell/histiocyte-rich B-cell lymphoma in a patient with recurrent hairy cell leukemia: a case report	irrelevent reports	Oxford Medical Case Reports
Koliev, V. I., I. E. Sarapulova and I. I. Shaposhnik	- 2021	Probable etiological factors for the development of chronic heart failure in patients with chronic obstructive pulmonary disease	irrelevent reports	Profilakticheskaya Meditsina
Nishino, M., Y. Egami, S. Kawanami, M. Abe, M. Ohsuga, H. Nohara, K. Ukita, A. Kawamura, K. Yasumoto, M. Tsuda, N. Okamoto, Y. Matsunaga-Lee and M. Yano	- 2024	Prognostic Comparison of Octogenarian vs. Non-Octogenarian With Acute Decompensated Heart Failure — AURORA Study —	irrelevent reports	Circulation Journal
Oeun, B., S. Hikoso, D. Nakatani, H. Mizuno, S. Suna, T. Kitamura, K. Okada, T. Dohi, Y. Sotomi, T. Kojima, H. Kida, A. Sunaga, T. Sato, Y. Takeda, H. Kurakami, T. Yamada, S. Tamaki, H. Abe, Y. Nakagawa, Y. Higuchi, H. Fuji, T. Mano, M. Uematsu, Y. Yasumura, T. Yamada and Y. Sakata	- 2022	Prognostic Impact of Echocardiographic Diastolic Dysfunction on Outcomes in Patients with Heart Failure with Preserved Ejection Fraction - Insights From the PURSUIT-HFpEF Registry	irrelevent reports	Circulation Journal
Seo, J. H., D. Hong, T. Youn, S. H. Lee, K. H. Choi, D. Kim, T. K. Park, J. M. Lee, Y. B. Song, J. O. Choi, J. Y. Hahn, S. H. Choi, H. C. Gwon, E. S. Jeon and J. H. Yang	- 2022	Prognostic implications of coronary artery disease and stress tests in patients with elevated left ventricular filling pressure and preserved ejection fraction	irrelevent reports	Frontiers in Cardiovascular Medicine
Greene, S. J., D. Lautsch, L. Yang, X. I. Tan and J. E. Brady	- 2022	Prognostic Interplay Between COVID-19 and Heart Failure With Reduced Ejection Fraction	irrelevent reports	Journal of Cardiac Failure
Zencirkiran Agus, H. and S. Kahraman	- 2020	Prognostic nutritional index predicts one-year outcome in heart failure with preserved ejection fraction	irrelevent reports	Acta Cardiologica
Fuchida, A., S. Suzuki, H. Motoki, Y. Kanzaki, T. Maruyama, N. Hashizume, A. Kozuka, K. Yahikozawa and K. Kuwahara	- 2021	Prognostic significance of diastolic blood pressure in patients with heart failure with preserved ejection fraction	irrelevent reports	Heart and Vessels
Zhao, Z., D. Qi, Z. Zhang, X. Du, F. Zhang, R. Ma, Y. Liang, Y. Zhao, Y. Gao and Y. Yang	- 2024	Prognostic Value of Inflammatory Cytokines in Predicting Hospital Readmissions in Heart Failure with Preserved Ejection Fraction	irrelevent reports	Journal of Inflammation Research
Kocabas, U., H. Altay, F. Ozkalayci, O. Yildirimturk, E. Kulah and S. Pehlivanoglu	- 2020	Prognostic value of worsening renal function in patients with acute decompensated heart failure with preserved ejection fraction and its association with increased inflammatory state	irrelevent reports	International Journal of the Cardiovascular Academy
Abernethy, A., S. Raza, J. L. Sun, K. J. Anstrom, R. Tracy, J. Steiner, P. VanBuren and M. M. LeWinter	- 2018	Pro-Inflammatory Biomarkers in Stable Versus Acutely Decompensated Heart Failure With Preserved Ejection Fraction	irrelevent reports	J Am Heart Assoc
Kumar, P., A. Lim, S. L. Poh, S. N. Hazirah, C. J. H. Chua, N. B. Sutamam, T. Arkachaisri, J. G. Yeo, T. Kofidis, V. Sorokin, C. S. P. Lam, A. M. Richards and S. Albani	- 2022	Pro-Inflammatory Derangement of the Immuno-Interactome in Heart Failure	irrelevent reports	Front Immunol
Kresoja, K. P., K. P. Rommel, R. Wachter, S. Henger, C. Besler, N. Klöting, M. Schnelle, A. Hoffmann, P. Büttner, U. Ceglarek, H. Thiele, M. Scholz, F. Edelmann, M. Blüher and P. Lurz	- 2021	Proteomics to improve phenotyping in obese patients with heart failure with preserved ejection fraction	irrelevent reports	European Journal of Heart Failure

Hirata, Y., E. Yamamoto, T. Tokitsu, H. Kusaka, K. Fujisue, H. Kurokawa, K. Sugamura, H. Maeda, K. Tsujita, M. Yamamuro, K. Kaikita, S. Hokimoto, S. Sugiyama and H. Ogawa	- 2015	Reactive oxidative metabolites are associated with the severity of heart failure and predict future cardiovascular events in heart failure with preserved left ventricular ejection fraction	irrelevent reports	International Journal of Cardiology
Mazumder, S., R. Karmali, P. Sankar, F. Berglund, M. Majid, A. Syed, A. Paul and A. Klein	- 2023	Recurrent Pericardial Effusions and Pericarditis Due to Yellow Nail Syndrome	irrelevent reports	JACC: Case Reports
Suades, R., A. Vilella-Figuerola, T. Padró, S. Mirabet and L. Badimon	- 2023	Red Blood Cells and Endothelium Derived Circulating Extracellular Vesicles in Health and Chronic Heart Failure: A Focus on Phosphatidylserine Dynamics in Vesiculation	irrelevent reports	International Journal of Molecular Sciences
Stamerra, C. A., E. D'Elia, M. Gori, F. Roncali, A. Cereda, A. Gavazzi, C. Ferri and M. Senni	- 2023	Red cell distribution width (RDW) is correlated to time of oxygen desaturation < 90% and length of sleep apneas in patients with sleep disorder breathing (SDB) and acute heart failure with preserved ejection fraction (HFpEF)	irrelevent reports	Frontiers in Cardiovascular Medicine
Tan, E. S. J., S. P. Chan, Y. C. Choi, C. J. Pemberton, R. Troughton, K. Poppe, M. Lund, G. Devlin, R. N. Doughty and A. M. Richards	- 2023	Regional Handling and Prognostic Performance of Circulating Insulin-Like Growth Factor Binding Protein-7 in Heart Failure	irrelevent reports	JACC: Heart Failure
Tan, E. S. J., S. P. Chan, Y. C. Choi, C. J. Pemberton, R. Troughton, K. Poppe, M. Lund, G. Devlin, R. N. Doughty and A. M. Richards	- 2023	Regional Handling and Prognostic Performance of Circulating Insulin-Like Growth Factor Binding Protein-7 in Heart Failure	irrelevent reports	JACC Heart Fail
Vausort, M., M. Niedolistek, A. I. Lumley, M. Oknińska, A. Paterek, M. Mączewski, X. Dong, C. Jäger, C. L. Linster, P. Leszek and Y. Devaux	- 2022	Regulation of N6-Methyladenosine after Myocardial Infarction	irrelevent reports	Cells
Gottdiener, J. S., P. Buzkova, P. A. Kahn, C. DeFilippi, S. Shah, E. Barasch, J. R. Kizer, B. Psaty and J. M. Gardin	- 2022	Relation of Cigarette Smoking and Heart Failure in Adults ≥65 Years of Age (From the Cardiovascular Health Study)	irrelevent reports	American Journal of Cardiology
Gottdiener, J. S., P. Buzkova, P. A. Kahn, C. DeFilippi, S. Shah, E. Barasch, J. R. Kizer, B. Psaty and J. M. Gardin	- 2022	Relation of Cigarette Smoking and Heart Failure in Adults ≥65 Years of Age (From the Cardiovascular Health Study)	irrelevent reports	Am J Cardiol
Dahiya, R., S. P. Shultz, A. Dahiya, J. Fu, C. Flatley, D. Duncan, J. Cardinal, K. M. Kostner, N. M. Byrne, A. P. Hills, M. Harris, L. S. Conwell and G. M. Leong	- 2015	Relation of reduced preclinical left ventricular diastolic function and cardiac remodeling in overweight youth to insulin resistance and inflammation	irrelevent reports	Am J Cardiol
Sato, Y., A. Yoshihisa, M. Oikawa, T. Nagai, T. Yoshikawa, Y. Saito, K. Yamamoto, Y. Takeishi and T. Anzai	- 2019	Relation of systolic blood pressure on the following day with post-discharge mortality in hospitalized heart failure patients with preserved ejection fraction: A report from the JASPER registry	irrelevent reports	International Heart Journal
Aramburu-Bodas, Ó., B. Garciá-Casado, P. Salamanca-Bautista, M. E. Guisado-Espartero, J. L. Arias-Jiménez, A. Barco-Sánchez, J. C. Santamariá-González, F. Formiga, M. Montero-Pérez-Barquero and L. Manzano	- 2015	Relationship between osteoprotegerin and mortality in decompensated heart failure with preserved ejection fraction	irrelevent reports	Journal of Cardiovascular Medicine
Celik, A., F. Koc, H. Kadi, K. Ceyhan, U. Erkorkmaz, T. Burucu, M. Karayakali and O. Onalan	- 2012	Relationship between red cell distribution width and echocardiographic parameters in patients with diastolic heart failure	irrelevent reports	Kaohsiung Journal of Medical Sciences
Shurkevich, N. P., A. S. Vetoshkin, L. I. Gapon, S. M. Dyachkov and A. A. Simonyan	- 2022	Relationship of arterial and cardiac stiffness in rotating shift workers in the Arctic	irrelevent reports	Arterial Hypertension (Russian Federation)

Prausmüller, S., G. Spinka, H. Arfsten, S. Stasek, R. Rettl, P. E. Bartko, G. Goliasch, G. Strunk, J. Riebandt, J. Mascherbauer, D. Bonderman, C. Hengstenberg, M. Hülsmann and N. Pavo	- 2021	Relevance of Neutrophil Neprilysin in Heart Failure	irrelevent reports	Cells
Dung, B. T., T. T. X. Anh, T. Hoa and N. M. Duc	- 2023	Remarkable and Prolonged Response to Melphalan and High-Dose Dexamethasone Combined Therapy of a Patient with Light-Chain Cardiac Amyloidosis	irrelevent reports	Biomedical Research and Therapy
Minaña, G., E. Santas, R. De La Espriella, E. Núñez, M. Lorenzo, G. Núñez, E. Valero, V. Bodí, F. J. Chorro, J. Sanchis, A. Cohen-Solal, A. Bayés-Genís and J. Núñez	- 2021	Right ventricular function and iron deficiency in acute heart failure	irrelevent reports	European Heart Journal: Acute Cardiovascular Care
Wang, Y. J., L. L. Ma, Y. Liu, Y. Yan, Y. Sun, Y. S. Wang, X. M. Dai, Z. F. Ji, L. Y. Ma, H. Y. Chen and L. D. Jiang	- 2021	Risk assessment model for heart failure in Chinese patients with Takayasu's arteritis	irrelevent reports	Clinical Rheumatology
Bansal, N., L. R. Zelnick, R. Scherzer, M. Estrella and M. G. Shlipak	- 2024	Risk factors and outcomes associated with heart failure with preserved and reduced ejection fraction in people with chronic kidney disease	irrelevent reports	Circulation: Heart Failure
Lin, Y., X. Zhong, M. Liu, S. Zhang, Z. Xiong, Y. Huang, Y. Fan, X. Xu, Y. Guo, Y. Li, X. Sun, H. Zhou, D. Yang, X. Ye, X. Liao and X. Zhuang	- 2022	Risk Stratification and Efficacy of Spironolactone in Patients with Heart Failure with Preserved Ejection Fraction: Secondary Analysis of the TOPCAT Randomized Clinical Trial	irrelevent reports	Cardiovascular Drugs and Therapy
Kotwica, T., J. Relewicz, A. Rojek, M. Tupikowska-Marzec, M. Kabaj, B. Karolko, J. Maj, G. Bednarek-Tupikowska, W. Kosmala, J. C. Szepietowski and M. Przewlocka-Kosmala	- 2019	Role of galectin-3 in subclinical myocardial impairment in psoriasis	irrelevent reports	J Eur Acad Dermatol Venereol
Temporelli, P. L.	- 2024	Role of glucagon-like peptide-1 agonists in obesity and heart failure with preserved ejection fraction	irrelevent reports	European Heart Journal, Supplement
El Mallawany, H., M. I. Mahmoud, T. S. Morsi and R. M. El-Shiekh	- 2014	Role of N-terminal pro B-type natriuretic peptide in acute exacerbation of chronic obstructive pulmonary disease	irrelevent reports	Egyptian Journal of Chest Diseases and Tuberculosis
Malatiali, S. A. and J. S. Juggi	- 1995	Role of polymorphonuclear leukocytes in reperfusion injury of globally ischemic rat heart	irrelevent reports	Can J Cardiol
von Bibra, H., M. Diamant, P. G. Scheffer, T. Siegmund and P. M. Schumm-Draeger	- 2008	Rosiglitazone, but not glimepiride, improves myocardial diastolic function in association with reduction in oxidative stress in type 2 diabetic patients without overt heart disease	irrelevent reports	Diab Vasc Dis Res
Zhu, M., J. Guo, B. Qiqike, X. Nay, S. Dan, A. Kuransi, G. Hu, Z. Han, D. Hou, A. Aili, B. Xia, P. Chen, B. Bate and J. Xie	- 2023	Sacubitril/Valsartan Cannot Improve Cardiac Function Compared with Valsartan in Patients Suffering Nonvalvular Atrial Fibrillation without Systolic Heart Failure	irrelevent reports	International Heart Journal
Sivri, F., Y. K. Icen, H. Koca, M. Coşkun, M. Ardınç, O. Deniz, F. N. Arici, M. Koc and H. Güngör	- 2023	Selvester QRS Score is a Predictor of Mortality in Heart Failure with Preserved Ejection Fraction	irrelevent reports	Arq Bras Cardiol
Petrie, M. C., B. A. Borlaug, J. Butler, M. J. Davies, D. W. Kitzman, S. J. Shah, S. Verma, T. J. Jensen, M. N. Einfeldt, K. Liisberg, E. Perna, K. Sharma, J. A. Ezekowitz, M. Fu, V. Melenovský, H. Ito, M. Lelonek and M. N. Kosiborod	- 2024	Semaglutide and NT-proBNP in Obesity-Related HFpEF: Insights From the STEP-HFpEF Program	irrelevent reports	Journal of the American College of Cardiology

Borlaug, B. A., D. W. Kitzman, M. J. Davies, S. Rasmussen, E. Barros, J. Butler, M. N. Einfeldt, G. K. Hovingh, D. V. Møller, M. C. Petrie, S. J. Shah, S. Verma, W. Abhayaratna, F. Z. Ahmed, V. Chopra, J. Ezekowitz, M. Fu, H. Ito, M. Lelonek, V. Melenovsky, J. Núñez, E. Perna, M. Schou, M. Senni, P. van der Meer, D. Von Lewinski, D. Wolf and M. N. Kosiborod	- 2023	Semaglutide in HFpEF across obesity class and by body weight reduction: a prespecified analysis of the STEP-HFpEF trial	irrelevent reports	Nat Med
Kosiborod, M. N., S. Z. Abildstrøm, B. A. Borlaug, J. Butler, S. Rasmussen, M. Davies, G. K. Hovingh, D. W. Kitzman, M. L. Lindegaard, D. V. Møller, S. J. Shah, M. B. Treppendahl, S. Verma, W. Abhayaratna, F. Z. Ahmed, V. Chopra, J. Ezekowitz, M. Fu, H. Ito, M. Lelonek, V. Melenovsky, B. Merkely, J. Núñez, E. Perna, M. Schou, M. Senni, K. Sharma, P. Van Der Meer, D. Von Lewinski, D. Wolf and M. C. Petrie	- 2023	Semaglutide in Patients with Heart Failure with Preserved Ejection Fraction and Obesity	irrelevent reports	New England Journal of Medicine
Butler, J., S. Z. Abildstrøm, B. A. Borlaug, M. J. Davies, D. W. Kitzman, M. C. Petrie, S. J. Shah, S. Verma, W. P. Abhayaratna, V. Chopra, J. A. Ezekowitz, M. Fu, H. Ito, M. Lelonek, J. Núñez, E. Perna, M. Schou, M. Senni, P. van der Meer, D. von Lewinski, D. Wolf, R. L. Altschul, S. Rasmussen and M. N. Kosiborod	- 2023	Semaglutide in Patients With Obesity and Heart Failure Across Mildly Reduced or Preserved Ejection Fraction	irrelevent reports	Journal of the American College of Cardiology
Kosiborod, M. N., M. C. Petrie, B. A. Borlaug, J. Butler, M. J. Davies, G. K. Hovingh, D. W. Kitzman, D. V. Møller, M. B. Treppendahl, S. Verma, T. J. Jensen, K. Liisberg, M. L. Lindegaard, W. Abhayaratna, F. Z. Ahmed, T. Ben-Gal, V. Chopra, J. A. Ezekowitz, M. Fu, H. Ito, M. Lelonek, V. Melenovský, B. Merkely, J. Núñez, E. Perna, M. Schou, M. Senni, K. Sharma, P. van der Meer, D. Von Lewinski, D. Wolf and S. J. Shah	- 2024	Semaglutide in Patients with Obesity-Related Heart Failure and Type 2 Diabetes	irrelevent reports	New England Journal of Medicine
Butler, J., S. J. Shah, M. C. Petrie, B. A. Borlaug, S. Z. Abildstrøm, M. J. Davies, G. K. Hovingh, D. W. Kitzman, D. V. Møller, S. Verma, M. N. Einfeldt, M. L. Lindegaard, S. Rasmussen, W. Abhayaratna, F. Z. Ahmed, T. Ben-Gal, V. Chopra, J. A. Ezekowitz, M. Fu, H. Ito, M. Lelonek, V. Melenovský, B. Merkely, J. Núñez, E. Perna, M. Schou, M. Senni, K. Sharma, P. van der Meer, D. Von Lewinski, D. Wolf and M. N. Kosiborod	- 2024	Semaglutide versus placebo in people with obesity-related heart failure with preserved ejection fraction: a pooled analysis of the STEP-HFpEF and STEP-HFpEF DM randomised trials	irrelevent reports	The Lancet

Manso, M. C., D. P. Marques, S. L. Rocha, S. C. Rodeia and R. Domingos	- 2017	Senile systemic amyloidosis: An underdiagnosed disease	irrelevent reports	European Journal of Case Reports in Internal Medicine
Karpiński, L., R. Płaksej, W. Kosmala and M. Witkowska	- 2008	Serum levels of interleukin-6, interleukin-10 and C-reactive protein in relation to left ventricular function in patients with myocardial infarction treated with primary angioplasty	irrelevent reports	Kardiol Pol
Ben-Dor, I., M. Haim, E. Rechavia, D. Murninkas, D. Harell, A. Porter, Z. Iakobishvili, A. Battler and D. Hasdai	- 2006	Serum NT-proBNP concentrations in the early phase do not predict the severity of systolic or diastolic left ventricular dysfunction among patients with ST-elevation acute myocardial infarction	irrelevent reports	Angiology
Lepojärvi, E. S., O. P. Piira, E. Pääkkö, E. Lammentausta, J. Risteli, J. A. Miettinen, J. S. Perkiömäki, H. V. Huikuri and M. J. Junttila	- 2015	Serum PINP, PIIINP, galectin-3, and ST2 as surrogates of myocardial fibrosis and echocardiographic left venticular diastolic filling properties	irrelevent reports	Frontiers in Physiology
Nogi, S., S. I. Fujita, Y. Okamoto, S. Kizawa, H. Morita, T. Ito, K. Sakane, K. Sohmiya, M. Hoshiga and N. Ishizaka	- 2015	Serum uric acid is associated with cardiac diastolic dysfunction among women with preserved ejection fraction	irrelevent reports	American Journal of Physiology - Heart and Circulatory Physiology
Yang, C. D., S. Feng, J. W. Chen, M. Aihemaiti, X. Y. Shu, J. W. Quan, F. H. Ding, L. Lu, W. F. Shen, R. Y. Zhang and X. Q. Wang	- 2022	Serum Uric Acid Is Associated with the Progression of Left Ventricular Diastolic Dysfunction in Apparently Healthy Subjects	irrelevent reports	Disease Markers
Li, L., M. Sun and Y. Zhang	- 2016	Serum uric acid, NT-probnp and hs-CRP as biomarkers in chronic heart failure	irrelevent reports	International Journal of Clinical and Experimental Medicine
Li, J., P. Liu, H. Li, Y. Wang, Y. Chen, R. Qi and Y. Li	- 2019	Sevoflurane Preconditioning Prevents Septic Myocardial Dysfunction in Lipopolysaccharide-Challenged Mice	irrelevent reports	J Cardiovasc Pharmacol
Lau, E. S., T. Cunningham, K. M. Hardin, E. Liu, R. Malhotra, M. Nayor, G. D. Lewis and J. E. Ho	- 2020	Sex Differences in Cardiometabolic Traits and Determinants of Exercise Capacity in Heart Failure With Preserved Ejection Fraction	irrelevent reports	JAMA Cardiol
Grakova, E. V., K. V. Kopieva, A. M. Gusakova, A. V. Smorgon, A. N. Maltseva, A. V. Mochula, A. V. Svarovskaya and K. V. Zavadovsky	- 2023	Significance of catestatin in the pathogenesis of heart failure with preserved ejection fraction in patients with non-obstructive coronary artery disease	irrelevent reports	Russian Journal of Cardiology
Conti, V., G. Corbi, M. V. Polito, M. Ciccarelli, V. Manzo, M. Torsiello, E. De Bellis, F. D'Auria, G. Vitulano, F. Piscione, A. Carrizzo, P. Di Pietro, C. Vecchione, N. Ferrara and A. Filippelli	- 2020	Sirt1 Activity in PBMCs as a Biomarker of Different Heart Failure Phenotypes	irrelevent reports	Biomolecules
Hitsumoto, T.	- 2020	Skin Autofluorescence as a Predictor of First Heart Failure Hospitalization in Patients With Heart Failure With Preserved Ejection Fraction	irrelevent reports	Cardiology Research
Vazir, A. and C. J. Kapelios	- 2023	Sleep-disordered breathing and cardiovascular disease: Who and why to test and how to intervene?	irrelevent reports	Heart
Cavka, L., U. Bencak Ferko, N. Pitz, Z. Trpkovski and M. Lainscak	- 2021	Sodium–glucose cotransporter 2 inhibitor-induced euglycaemic diabetic ketoacidosis in heart failure with preserved ejection fraction	irrelevent reports	ESC Heart Failure
Al-Kindi, S. G., P. Buzkova, S. G. Shitole, A. P. Reiner, P. K. Garg, J. S. Gottdiener, B. M. Psaty and J. R. Kizer	- 2020	Soluble CD14 and Risk of Heart Failure and Its Subtypes in Older Adults	irrelevent reports	Journal of Cardiac Failure
You, Y., S. Huang, H. Liu, C. Fan, K. Liu and Z. Wang	- 2021	Soluble fibrinogen-like protein 2 levels are decreased in patients with ischemic heart failure and associated with cardiac function	irrelevent reports	Molecular Medicine Reports
Sugano, A., Y. Seo, T. Ishizu, S. Sai, M. Yamamoto, Y. Hamada-Harimura, T. Machino-Ohtsuka, K. Obara, I. Nishi, K. Aonuma and A. Nogami	- 2019	Soluble ST2 and brain natriuretic peptide predict different mode of death in patients with heart failure and preserved ejection fraction	irrelevent reports	Journal of Cardiology
Peeples, J., M. Byku, A. L. Hinderliter, S. V. Smith, V. K. Derebail, L. L. Balos, R. C. Venuto and M. K. Saha	- 2021	Sparkling myocardium in a hemodialysis patient	irrelevent reports	Kidney International

Seko, Y., T. Kato, T. Morimoto, H. Yaku, Y. Inuzuka, Y. Tamaki, N. Ozasa, M. Shiba, E. Yamamoto, Y. Yoshikawa, Y. Yamashita, T. Kitai, R. Taniguchi, M. Iguchi, K. Nagao, T. Kawai, A. Komasa, R. Nishikawa, Y. Kawase, T. Morinaga, M. Toyofuku, Y. Furukawa, K. Ando, K. Kadota, Y. Sato, K. Kuwahara and T. Kimura	- 2022	Starting Neurohormonal Antagonists in Patients With Acute Heart Failure With Mid-Range and Preserved Ejection Fraction	irrelevent reports	Circulation Journal
Elena, B., M. C. María Izarbe, O. G. Susana, M. G. Sebastián, S. M. José Luis, D. M. José María and T. C. Miguel Ángel	- 2021	Study of Cellular Aging in a Cohort of Patients with Heart Failure	irrelevent reports	High Blood Pressure and Cardiovascular Prevention
Mathews, S. E., J. Castellanos-Diaz, A. Srihari, S. Kadiyala, J. Leey-Casella, H. K. Ghayee and A. Ogunsakin	- 2021	Subacute Thyroiditis and Heart Failure in a Patient Presenting With COVID-19	irrelevent reports	Journal of Investigative Medicine High Impact Case Reports
Zhao, L., R. Zierath, J. E. John, B. L. Claggett, M. E. Hall, D. Clark, K. R. Butler, A. Correa and A. M. Shah	- 2022	Subclinical Risk Factors for Heart Failure with Preserved and Reduced Ejection Fraction among Black Adults	irrelevent reports	JAMA Network Open
Prandi, F. R., A. N. Zaidi, G. Larocca, M. Hadley, M. Riasat, M. O. Anastasius, P. R. Moreno, S. Sharma, A. Kini, R. Murthy, P. Boateng and S. Lerakis	- 2022	Sudden Cardiac Arrest in an Adult with Anomalous Origin of the Left Coronary Artery from the Pulmonary Artery (ALCAPA): Case Report	irrelevent reports	International Journal of Environmental Research and Public Health
Chen, H., M. Tesic, V. N. Nikolic, M. Pavlovic, R. M. Vucic, A. Spasic, H. Jovanovic, I. Jovanovic, S. E. L. Town, M. P. Padula and L. McClements	- 2022	Systemic Biomarkers and Unique Pathways in Different Phenotypes of Heart Failure with Preserved Ejection Fraction	irrelevent reports	Biomolecules
Hegner, P., M. Wester, M. Tafelmeier, Z. Provaznik, S. Klatt, C. Schmid, L. S. Maier, M. Arzt, S. Wagner and S. Lebek	- 2024	Systemic inflammation predicts diastolic dysfunction in patients with sleep disordered breathing	irrelevent reports	Eur Respir J
Xu, L., G. Chen, Y. Liang, C. Zhou, F. Zhang, T. Fan, X. Chen, H. Zhou and W. Yuan	- 2021	T helper 17 cell responses induce cardiac hypertrophy and remodeling in essential hypertension	irrelevent reports	Pol Arch Intern Med
Kasbekar, M., V. Nardi, P. D. Cin, A. M. Brunner, M. Burke, Y. B. Chen, C. Connolly, A. T. Fathi, J. Foster, M. MacRae, S. L. McAfee, K. McGregor, R. Narayan, A. Y. Ramos, T. T. Som, M. Vartanian, R. S. Friedman, K. A. Benhadji and G. S. Hobbs	- 2020	Targeted FGFR inhibition results in a durable remission in an FGFR1-driven myeloid neoplasm with eosinophilia	irrelevent reports	Blood Advances
Russomanno, G., G. Corbi, V. Manzo, N. Ferrara, G. Rengo, A. A. Puca, S. Latte, A. Carrizzo, M. C. Calabrese, R. Andriantsitohaina, W. Filippelli, C. Vecchione, A. Filippelli and V. Conti	- 2017	The anti-ageing molecule sirt1 mediates beneficial effects of cardiac rehabilitation	irrelevent reports	Immunity and Ageing
Koza, Y.	- 2014	The association between C-reactive protein level and left ventricular diastolic functions in patients with first ST-segment elevation myocardial infarction treated by primary coronary intervention	irrelevent reports	J Cardiol
Tucker, W., R. L. McClelland, M. A. Allison, M. Szklo, K. A. Rye and K. L. Ong	- 2023	The association of circulating fibroblast growth factor 21 levels with incident heart failure: The Multi-Ethnic Study of Atherosclerosis	irrelevent reports	Metabolism: Clinical and Experimental

Takvorian, K. S., D. Wang, P. Courchesne, R. S. Vasan, E. J. Benjamin, S. Cheng, M. G. Larson, D. Levy and J. E. Ho	- 2023	The Association of Protein Biomarkers With Incident Heart Failure With Preserved and Reduced Ejection Fraction	irrelevent reports	Circ Heart Fail
Zhu, D., C. Wang, Y. Zhou, H. Che, R. Wang, L. Cheng, C. Rao, Q. Zhong, Z. Li, Y. Duan and K. He	- 2024	The Associations of Two Novel Inflammation Biomarkers, SIRI and SII, with Mortality Risk in Patients with Chronic Heart Failure	irrelevent reports	Journal of Inflammation Research
Bjerring, A. W., S. D. Fosså, H. S. Haugnes, R. Nome, T. M. Stokke, K. H. Haugaa, C. E. Kiserud, T. Edvardsen and S. I. Sarvari	- 2021	The cardiac impact of cisplatin-based chemotherapy in survivors of testicular cancer: A 30-year follow-up	irrelevent reports	European Heart Journal Cardiovascular Imaging
Komorita, T., E. Yamamoto, D. Sueta, T. Tokitsu, K. Fujisue, H. Usuku, T. Nishihara, F. Oike, M. Takae, K. Egashira, S. Takashio, M. Ito, K. Yamanaga, Y. Arima, K. Sakamoto, S. Suzuki, K. Kaikita and K. Tsujita	- 2020	The controlling nutritional status score predicts outcomes of cardiovascular events in patients with heart failure with preserved ejection fraction	irrelevent reports	IJC Heart and Vasculature
Morgan, R. B., L. McCullagh, M. Barry and C. Daly	- 2017	The cost of inpatient management of heart failure patients: a microcosting study in the Irish healthcare setting	irrelevent reports	Irish Journal of Medical Science
Turgutkaya, A., A. Z. Bolaman and İ. Yavaşoğlu	- 2021	The Development of Transfusion-Related Acute Lung Injury for the Second Time: A New Awareness for Daratumumab?	irrelevent reports	Plasmatology
Michalski, B., P. Trzciński, K. Kupczyńska, D. Miśkowiec, Ł. Pęczek, B. Nawrot, P. Lipiec and J. D. Kasprzak	- 2017	The differences in the relationship between diastolic dysfunction, selected biomarkers and collagen turn-over in heart failure patients with preserved and reduced ejection fraction	irrelevent reports	Cardiology Journal
Stojanovic, D., V. Mitic, M. Stojanovic, D. Petrovic, A. Ignjatovic, M. Milojkovic, O. Dunjic, V. Bojanic and M. D. Ilic	- 2021	The Discriminatory Ability of Renalase and Biomarkers of Cardiac Remodeling for the Prediction of Ischemia in Chronic Heart Failure Patients With the Regard to the Ejection Fraction	irrelevent reports	Frontiers in Cardiovascular Medicine
Chung, I., D. Goyal, R. J. Macfadyen and G. Y. Lip	- 2008	The effects of maximal treadmill graded exercise testing on haemorheological, haemodynamic and flow cytometry platelet markers in patients with systolic or diastolic heart failure	irrelevent reports	Eur J Clin Invest
Chuda-Wietczak, A., A. Sakowicz, A. Tycinska, I. Bytyci and A. Bielecka-Dabrowa	- 2023	The GLVC scoring system: a single-center model for predicting survival and hospitalization in patients with heart failure	irrelevent reports	Irish Journal of Medical Science
Henkens, M. T. H. M., A. M. van Ommen, S. Remmelzwaal, G. B. Valstar, P. Wang, J. A. J. Verdonschot, M. R. Hazebroek, L. Hofstra, V. P. M. van Empel, J. W. J. Beulens, H. M. den Ruijter and S. R. B. Heymans	- 2022	The HFA-PEFF score identifies 'early-HFpEF' phenogroups associated with distinct biomarker profiles	irrelevent reports	ESC Heart Failure
Kim, J. H., Y. A. Ko, J. Hedley, J. MacNamara, M. Awad, W. Taylor, S. Healy, H. Aida, N. A. Le, P. W. Wilson, M. White, L. S. Sperling, J. S. Wilson, Jr. and A. L. Baggish	- 2017	The impact of moderate distance recreational running and ageing on cardiac physiology	irrelevent reports	Heart
Dahlen, B., A. Schulz, S. Göbel, S. O. Tröbs, S. Schwuchow-Thonke, H. M. Spronk, J. H. Prochaska, N. Arnold, K. J. Lackner, T. Gori, H. Ten Cate, T. Münzel, P. S. Wild and M. Panova-Noeva	- 2021	The impact of platelet indices on clinical outcome in heart failure: results from the MyoVasc study	irrelevent reports	ESC Heart Fail
Fukuda, Y., K. Nitta, S. Kurisu, N. Watanabe, H. Ikenaga, K. Ishibashi and Y. Nakano	- 2021	The Monocyte to High-Density Lipoprotein Cholesterol Ratio Is Associated with Left Ventricular Diastolic Function in Patients with No Significant Perfusion Abnormality	irrelevent reports	Int Heart J

Yamamoto, E., Y. Hirata, T. Tokitsu, H. Kusaka, K. Sakamoto, M. Yamamuro, K. Kaikita, H. Watanabe, S. Hokimoto, S. Sugiyama, T. Maruyama and H. Ogawa	- 2015	The pivotal role of eNOS uncoupling in vascular endothelial dysfunction in patients with heart failure with preserved ejection fraction	irrelevent reports	International Journal of Cardiology
Giro, P., K. D. Taylor, S. J. Shah and R. B. Patel	- 2023	The pK56M ICAM1 HFpEF risk variant and inflammatory biomarkers	irrelevent reports	American Heart Journal Plus: Cardiology Research and Practice
Yuan, X., C. Lv, S. Wu, H. Wang and X. Liu	- 2024	The Predictive Value of Hemoglobin to Red Cell Blood Distribution Width Ratio Combined with Serum Sodium for MACE of Acute Heart Failure with Preserved Ejection Fraction in Elderly Patients	irrelevent reports	International Journal of General Medicine
Wang, X., S. C. Butcher, J. H. Kuneman, R. P. Lustosa, F. Fortuni, N. Ajmone Marsan, J. Knuuti, J. J. Bax and V. Delgado	- 2022	The Quantity of Epicardial Adipose Tissue in Patients Having Ablation for Atrial Fibrillation With and Without Heart Failure	irrelevent reports	Am J Cardiol
Bayam, E., M. Kalçık, B. Öztürkeri, E. Yıldırım, A. Karaduman, S. Kalkan, A. Küp, N. Günay, A. Güner, M. Kahyaoğlu and C. Uyan	- 2021	The relationship between H2FPEF and SYNTAX scores in patients with non-ST elevation myocardial infarction	irrelevent reports	Acta Cardiologica
Seyfeli, E., B. Sarli, H. Saglam, C. Y. Karatas, E. Ozkan and M. Ugurlu	- 2016	The Relationship Between High-Sensitivity C-Reactive Protein Levels and Left Ventricular Hypertrophy in Patients With Newly Diagnosed Hypertension	irrelevent reports	J Clin Hypertens (Greenwich)
Kalinkina, T. V., N. V. Lareva, M. V. Chistyakova and V. V. Gorbunov	- 2020	The relationship of endothelial dysfunction with the development of diastolic heart failure in patients with hypertension	irrelevent reports	Rational Pharmacotherapy in Cardiology
Alpert, S., N. P. Lewis, H. Ross, M. Fowler and H. A. Valantine	- 1995	The relationship of granzyme A and perforin expression to cardiac allograft rejection and dysfunction	irrelevent reports	Transplantation
Fontes-Carvalho, R., F. Sampaio, M. Teixeira, V. Gama and A. F. Leite-Moreira	- 2015	The role of a structured exercise training program on cardiac structure and function after acute myocardial infarction: Study protocol for a randomized controlled trial	irrelevent reports	Trials
Kuryata, O. and O. Sirenko	- 2021	The role of testosterone deficiency in men with heart failure as a factor of changes in interaction between markers of inflammation, tissue fibrosis and advanced glycation end products	irrelevent reports	Romanian Journal of Diabetes, Nutrition and Metabolic Diseases
Ridker, P. M.	- 2023	The Time to Initiate Anti-Inflammatory Therapy for Patients With Chronic Coronary Atherosclerosis Has Arrived	irrelevent reports	Circulation
Hatab, I., M. Kneihsl, E. Bisping, P. P. Rainer, S. Fandler-Höfler, S. Eppinger, M. Haidegger, N. Berger, H. Mangge, R. Schmidt, C. Enzinger and T. Gattringer	- 2023	The value of clinical routine blood biomarkers in predicting long-term mortality after stroke	irrelevent reports	European Stroke Journal
Du, A., W. Zuo and P. Zhang	- 2020	Therapeutic Effects of Baoxin Capsules in Combination with Trimetazidine Tablets on Type 2 Diabetes Complicated with Chronic Heart Failure	irrelevent reports	Indian Journal of Pharmaceutical Sciences
Javaid, A., S. Sehgal and J. Dazley	- 2019	Three-valve endocarditis in a patient with bioprosthetic aortic valve replacement and intravenous drug use: A case report	irrelevent reports	European Heart Journal - Case Reports
Kim, M. K., B. Kim, J. Y. Lee, J. S. Kim, B. G. Han, S. O. Choi and J. W. Yang	- 2013	Tissue Doppler-derived E/e′ ratio as a parameter for assessing diastolic heart failure and as a predictor of mortality in patients with chronic kidney disease	irrelevent reports	Korean Journal of Internal Medicine
Yu, Q., R. Vazquez, S. Zabadi, R. R. Watson and D. F. Larson	- 2010	T-lymphocytes mediate left ventricular fibrillar collagen cross-linking and diastolic dysfunction in mice	irrelevent reports	Matrix Biol
Liu, S., Q. Yang, J. Liu, Q. Zhang and J. Zhang	- 2023	To assess the role of immune infiltrating immune cells of patients with chronic heart failure with atrial fibrillation and nursing care in these patients	irrelevent reports	Cellular and Molecular Biology
Yang, M. and A. Lopez	- 2023	Total parenteral nutrition pleural effusion after peripherally inserted central venous catheter insertion despite fluoroscopic confirmation	irrelevent reports	SAGE Open Medical Case Reports
Tuaima, T., A. J. Kinsara, A. Alrajawi, H. H. Allam and S. Alfakih	- 2020	Transient ST segment elevation in a patient with COVID-19 and a normal transthoracic echocardiogram	irrelevent reports	European Journal of Case Reports in Internal Medicine

Mohib, O., P. Clevenbergh, C. Truyens, M. Morissens and J. Castro Rodriguez	- 2020	Trichinella spiralis-associated myocarditis mimicking acute myocardial infarction	irrelevent reports	Acta Clinica Belgica: International Journal of Clinical and Laboratory Medicine
Lee, V. Y. C., J. T. H. Wong, H. C. Fan and V. T. F. Yeung	- 2012	Tuberculous pericarditis presenting as massive haemorrhagic pericardial effusion	irrelevent reports	BMJ Case Reports
Wong, S. W., J. K. X. Ng and Y. W. Chia	- 2021	Tuberculous pericarditis with tamponade diagnosed concomitantly with COVID-19: a case report	irrelevent reports	European Heart Journal - Case Reports
Toda, N., J. Takeoka, K. Tanigaki, H. Hirashima, M. Fujita and T. Komiya	- 2022	Two episodes of acute dyspnea that were induced by COVID-19 in a peritoneal dialysis patient	irrelevent reports	CEN Case Reports
Lebedev, D. A., E. A. Lyasnikova, E. Y. Vasilyeva, A. Y. Babenko and E. V. Shlyakhto	- 2020	Type 2 Diabetes Mellitus and Chronic Heart Failure with Midrange and Preserved Ejection Fraction: A Focus on Serum Biomarkers of Fibrosis	irrelevent reports	J Diabetes Res
Zhou, Q. F., F. Yang, Y. Dai, S. Chen, F. R. Zhang, L. Lu and Q. Y. Lu	- 2024	Tyrosine to threonine ratio was related to heart failure with reduced or mildly reduced ejection fraction	irrelevent reports	ESC Heart Failure
Mahfouz, R. A., M. Abdelhamed, I. Galal and M. Elsanan	- 2021	Usefulness of Stress-Derived E/e' Ratio in Asymptomatic Hypertensive Patients	irrelevent reports	Pulse
Mason, J. M., H. C. Hancock, H. Close, J. J. Murphy, A. Fuat, M. de Belder, R. Singh, A. Teggert, E. Wood, G. Brennan, N. Hussain, N. Kumar, N. Manshani, D. Hodges, D. Wilson and A. P. S. Hungin	- 2013	Utility of Biomarkers in the Differential Diagnosis of Heart Failure in Older People: Findings from the Heart Failure in Care Homes (HFinCH) Diagnostic Accuracy Study	irrelevent reports	PLoS ONE
Iida, M., M. Yamamoto, Y. S. Ishiguro, M. Yamazaki, N. Ueda, H. Honjo and K. Kamiya	- 2013	Utility of cystatin C as a marker for the severity of aortic regurgitation in hypertensive patients	irrelevent reports	Clin Chim Acta
Yokoyama, H., W. Shioyama, T. Shintani, S. Maeda, S. Hirobe, M. Maeda, Y. Sakata and Y. Fujio	- 2021	Vascular endothelial growth factor receptor inhibitors impair left ventricular diastolic functions	irrelevent reports	International Heart Journal
Thadhani, R., E. Appelbaum, Y. Chang, Y. Pritchett, I. Bhan, R. Agarwal, C. Zoccali, C. Wanner, D. Lloyd-Jones, J. Cannata, T. Thompson, P. Audhya, D. Andress, W. Zhang, J. Ye, D. Packham, B. Singh, D. Zehnder, W. J. Manning, A. Pachika and S. D. Solomon	- 2011	Vitamin D receptor activation and left ventricular hypertrophy in advanced kidney disease	irrelevent reports	Am J Nephrol
Nikolova, M., N. Nazifova-Tasinova, D. Vankova, D. Gerova, Y. Yotov, A. Atanasov, M. Pasheva, Y. Kiselova-Kaneva and B. Galunska	- 2021	Vitamin d status in patients with atrial fibrillation and heart failure - is there a link?	irrelevent reports	Clinical Laboratory
Trachsel, L. D., M. Boidin, C. Henri, A. Fortier, J. Lalongé, M. Juneau, A. Nigam and M. Gayda	- 2021	Women and men with coronary heart disease respond similarly to different aerobic exercise training modalities: a pooled analysis of prospective randomized trials	irrelevent reports	Appl Physiol Nutr Metab
Harada, A., Y. Mizuno, M. Ishii, T. Ishida, T. Yamada, F. Kugimiya and H. Yasue	- 2022	β-Blockers are associated with increased B-type natriuretic peptide levels differently in men and women in heart failure with preserved ejection fraction	irrelevent reports	American Journal of Physiology - Heart and Circulatory Physiology
Hikoso, S., H. Kida, A. Sunaga, D. Nakatani, K. Okada, T. Dohi, Y. Sotomi, B. Oeun, T. Sato, Y. Matsuoka, T. Kitamura, T. Yamada, H. Kurakami, S. Tamaki, M. Seo, M. Yano, T. Hayashi, A. Nakagawa, Y. Nakagawa, T. Yamada, Y. Yasumura and Y. Sakata	- 2024	β-blockers may be detrimental in frail patients with heart failure with preserved ejection fraction	irrelevent reports	Clinical Research in Cardiology

van den Hoogen, P., S. C. A. de Jager, M. M. H. Huibers, A. H. Schoneveld, Y. M. Puspitasari, G. B. Valstar, M. I. F. J. Oerlemans, R. A. de Weger, P. A. Doevendans, H. M. den Ruijter, J. D. Laman, A. Vink and J. P. G. Sluijter	- 2019	Increased circulating IgG levels, myocardial immune cells and IgG deposits support a role for an immune response in pre- and end-stage heart failure	irrelevent reports	Journal of Cellular and Molecular Medicine
Al Badri, A., K. Lai, J. Wei, S. Landes, P. K. Mehta, Q. Li, D. Johnson, S. E. Reis, S. F. Kelsey, V. Bittner, G. Sopko, L. J. Shaw, C. J. Pepine and C. N. B. Merz	- 2017	Inflammatory biomarkers as predictors of heart failure in women without obstructive coronary artery disease: A report from the NHLBI-sponsored Women's Ischemia Syndrome Evaluation (WISE)	irrelevent reports	PLoS ONE
Abudoukelimu, M., B. Ba, Y. Kai Guo and J. Xu	- 2022	Von Willebrand factor (vWF) in patients with heart failure with preserved ejection fraction (HFpEF): A retrospective observational study	irrelevent topic	Medicine (Baltimore)
van de Bovenkamp, A. A., N. Wijkstra, F. P. T. Oosterveer, A. Vonk Noordegraaf, H. J. Bogaard, A. C. van Rossum, F. S. de Man, B. A. Borlaug and M. L. Handoko	- 2022	The Value of Passive Leg Raise During Right Heart Catheterization in Diagnosing Heart Failure With Preserved Ejection Fraction	irrelevent topic	Circ Heart Fail
Mooney, L., C. E. Jackson, C. Adamson, A. McConnachie, P. Welsh, R. C. Myles, J. J. V. McMurray, P. S. Jhund, M. C. Petrie and N. N. Lang	- 2023	Adverse Outcomes Associated With Interleukin-6 in Patients Recently Hospitalized for Heart Failure With Preserved Ejection Fraction	irrelevent topic	Circ Heart Fail
Ovchinnikov, A., A. Filatova, A. Potekhina, T. Arefieva, A. Gvozdeva, F. Ageev and E. Belyavskiy	- 2023	Blood Immune Cell Alterations in Patients with Hypertensive Left Ventricular Hypertrophy and Heart Failure with Preserved Ejection Fraction	irrelevent topic	Journal of Cardiovascular Development and Disease
Almengló, C., X. Fu, M. T. Flores-Arias, L. Fernández Á, J. E. Viñuela, J. M. Martínez-Cereijo, D. Durán, M. Rodríguez-Mañero, J. R. González-Juanatey and S. Eiras	- 2022	Synergism between obesity and HFpEF on neutrophils phenotype and its regulation by adipose tissue-molecules and SGLT2i dapagliflozin	irrelevent topic	J Cell Mol Med
Gui, X. Y. and S. W. Rabkin	- 2023	C-Reactive Protein, Interleukin-6, Trimethylamine-N-Oxide, Syndecan-1, Nitric Oxide, and Tumor Necrosis Factor Receptor-1 in Heart Failure with Preserved Versus Reduced Ejection Fraction: a Meta-Analysis	meta analysis	Curr Heart Fail Rep
Nadarajah, R., T. Younsi, E. Romer, K. Raveendra, Y. M. Nakao, K. Nakao, F. Shuweidhi, D. C. Hogg, R. Arbel, D. Zahger, Z. Iakobishvili, G. C. Fonarow, M. C. Petrie, J. Wu and C. P. Gale	- 2023	Prediction models for heart failure in the community: A systematic review and meta-analysis	meta analysis	European Journal of Heart Failure
Vakhshoori, M., N. Bondariyan, S. Sabouhi, K. Kiani, N. Alaei Faradonbeh, S. A. Emami, M. Shakarami, F. Khanizadeh, S. Sanaei, N. Motamedi and D. Shafie	- 2024	The impact of platelet-to-lymphocyte ratio on clinical outcomes in heart failure: a systematic review and meta-analysis	meta analysis	Therapeutic Advances in Cardiovascular Disease
Li, S., X. Zhu, Y. Zhang, F. Li and S. Guo	- 2023	Validation of heart failure algorithm for diagnosing heart failure with preserved ejection fraction: a meta-analysis	meta analysis	ESC Heart Fail
Wang, R., J. Wu, H. Ye, X. Zhang and L. Wang	- 2022	Application Value of Systemic Inflammatory Indexes in the Clinical Evaluation of Patients with Heart Failure with Preserved Ejection Fraction (HFpEF)	no relevant outcomes	Medicina (Kaunas)
Jirak, P., R. Pistulli, M. Lichtenauer, B. Wernly, V. Paar, L. J. Motloch, R. Rezar, C. Jung, U. C. Hoppe, P. C. Schulze, D. Kretzschmar, R. C. Braun-Dullaeus and T. Bekfani	- 2020	Expression of the novel cardiac biomarkers sST2, GDF-15, suPAR, and H-FABP in HFpEF patients compared to ICM, DCM, and controls	no relevant outcomes	Journal of Clinical Medicine
Zhou, R., Y. Y. Xia, Z. Li, L. D. Wu, Y. Shi, Z. Y. Ling and J. X. Zhang	- 2024	HFpEF as systemic disease, insight from a diagnostic prediction model reminiscent of systemic inflammation and organ interaction in HFpEF patients	no relevant outcomes	Sci Rep

Deng, X. L., H. W. Yi, J. Xiao, X. F. Zhang, J. Zhao, M. Sun, X. S. Wen, Z. Q. Liu, L. Gao, Z. Y. Li, P. Ge, Q. Yu and D. Y. Zhang	- 2023	Serum uric acid: A risk factor for right ventricular dysfunction and prognosis in heart failure with preserved ejection fraction	no relevant outcomes	Frontiers in Endocrinology
Kaburova, A. N., O. M. Drapkina, S. M. Yudin, A. A. Yafarova, S. N. Koretsky, M. S. Pokrovskaya, V. V. Makarov, S. A. Kraevoy, B. B. Shoibonov, I. A. Efimova and Z. Z. Serebryanskaya	- 2022	The relationship between gut microbiota, chronic systemic inflammation, and endotoxemia in patients with heart failure with preserved ejection fraction	no relevant outcomes	Cardiovascular Therapy and Prevention (Russian Federation)
Bai, B., M. Cheng, L. Jiang, J. Xu, H. Chen and Y. Xu	- 2021	High Neutrophil to Lymphocyte Ratio and Its Gene Signatures Correlate With Diastolic Dysfunction in Heart Failure With Preserved Ejection Fraction	no relevant outcomes	Front Cardiovasc Med
Manilall, A., L. Mokotedi, S. Gunter, R. Le Roux, S. Fourie, C. A. Flanagan and A. M. E. Millen	- 2023	Tumor Necrosis Factor-α Mediates Inflammation-induced Early-Stage Left Ventricular Systolic Dysfunction	non-HFpEF	Journal of Cardiovascular Pharmacology
Mirolyubova, O. A., A. V. Kudryavtsev, E. O. Semchyugova, S. K. Malyutina and A. N. Ryabikov	- 2020	[C-reactive protein and its associations with cardiometabolic risk factors and echocardiographic indicators of heart failure: results of "Know your heart" study in Arkhangelsk]	non-HFpEF	Kardiologiia
Majmundar, M., T. Kansara, H. Park, G. Ibarra, J. Marta Lenik, P. Shah, A. Kumar, R. Doshi, H. Zala, S. Chaudhari and A. Kalra	- 2022	Absolute lymphocyte count as a predictor of mortality and readmission in heart failure hospitalization	non-HFpEF	Int J Cardiol Heart Vasc
Hou, M., L. Cao, Y. Ding, Y. Chen, B. Wang, J. Shen, W. Zhou, J. Huang, Q. Xu, H. Lv and L. Sun	- 2021	Neutrophil to Lymphocyte Ratio Is Increased and Associated With Left Ventricular Diastolic Function in Newly Diagnosed Essential Hypertension Children	non-HFpEF	Front Pediatr
Yıldız, A., M. Yüksel, M. Oylumlu, N. Polat, M. A. Akıl and H. Acet	- 2015	The association between the neutrophil/lymphocyte ratio and functional capacity in patients with idiopathic dilated cardiomyopathy	non-HFpEF	Anatol J Cardiol
Cherico, A. S., A. Rizvi, T. Jayakrishnan and P. Mewawalla	- 2022	Macroglossia as the initial presentation of AL amyloidosis: review and updates in treatment	review	BMJ Case Reports
Frey, P., C. Akret, D. Irles, A. Dompnier and A. C. Bing	- 2021	Pyoderma gangrenosumcomplicating a permanent pacemaker implantation: A case report and literature review	review	European Heart Journal - Case Reports
Yadav, R., L. Sun, M. Salyana, M. Eric, V. Gotlieb and J. C. Wang	- 2022	SMARCA4-Deficient Undifferentiated Tumor of Lung Mass—A Rare Tumor With the Rarer Occurrence of Brain Metastasis: A Case Report and Review of the Literature	review	Journal of Investigative Medicine High Impact Case Reports
Kataoka, N., T. Imamura, T. Koi, K. Uchida and K. Kinugawa	- 2024	Adverse Events Requiring Hospitalization Following Catheter Ablation for Atrial Fibrillation in Heart Failure with versus without Systolic Dysfunction	review	Journal of Cardiovascular Development and Disease
Zhang, B., Y. Xu, X. Cui, H. Jiang, W. Luo, X. Weng, Y. Wang, Y. Zhao, A. Sun and J. Ge	- 2021	Alteration of m6A RNA Methylation in Heart Failure With Preserved Ejection Fraction	review	Frontiers in Cardiovascular Medicine
Vanberg, P. and D. Atar	- 2010	Androgenic anabolic steroid abuse and the cardiovascular system	review	Handb Exp Pharmacol
Czapla, M., R. Juárez-Vela, K. Łokieć and P. Karniej	- 2021	The association between nutritional status and in-hospital mortality among patients with heart failure—a result of the retrospective nutritional status heart study 2 (Nshs2)	review	Nutrients
Lejeune, S., A. Ginion, N. Menghoum, D. Vancraeynest, A. Pasquet, B. L. Gerber, S. Horman, C. Beauloye and A. C. Pouleur	- 2023	Association of Plasma Myeloperoxidase with Inflammation and Diabetic status in HFpEF	review	Reviews in Cardiovascular Medicine
Kopecky, B. J. and K. J. Lavine	- 2024	Cardiac macrophage metabolism in health and disease	review	Trends Endocrinol Metab
Okerson, T. and R. J. Chilton	- 2012	The Cardiovascular Effects of GLP-1 Receptor Agonists	review	Cardiovascular Therapeutics
Güder, G. and F. H. Rutten	- 2014	Comorbidity of heart failure and chronic obstructive pulmonary disease: More than coincidence	review	Current Heart Failure Reports
Sokalski, V., D. Liu, K. Hu, S. Frantz and P. Nordbeck	- 2024	Echocardiographic predictors of outcome in severe aortic stenosis patients with preserved or reduced ejection fraction	review	Clinical Research in Cardiology

Frangogiannis, N. G.	- 2017	The extracellular matrix in myocardial injury, repair, and remodeling	review	J Clin Invest
George, M., A. Jena, V. Srivatsan, R. Muthukumar and V. E. Dhandapani	- 2016	GDF 15 - a novel biomarker in the offing for heart failure	review	Current Cardiology Reviews
Beatty, A. L., I. A. Ku, R. H. Christenson, C. R. DeFilippi, N. B. Schiller and M. A. Whooley	- 2013	High-sensitivity cardiac troponin T levels and secondary events in outpatients with coronary heart disease from the Heart and Soul Study	review	JAMA Intern Med
Wienecke, L. M., J. M. Leid, F. Leuschner and K. J. Lavine	- 2023	Imaging Targets to Visualize the Cardiac Immune Landscape in Heart Failure	review	Circulation: Cardiovascular Imaging
Zaidi, Y., E. G. Aguilar, M. Troncoso, D. V. Ilatovskaya and K. Y. DeLeon-Pennell	- 2021	Immune regulation of cardiac fibrosis post myocardial infarction	review	Cell Signal
Barth, Z., B. Nomeland Witczak, T. Schwartz, K. Gjesdal, B. Flatø, A. Koller, H. Sanner and I. Sjaastad	- 2016	In juvenile dermatomyositis, heart rate variability is reduced, and associated with both cardiac dysfunction and markers of inflammation: a cross-sectional study median 13.5 years after symptom onset	review	Rheumatology (Oxford)
Brouwers, F. P., R. A. De Boer, P. Van Der Harst, A. A. Voors, R. T. Gansevoort, S. J. Bakker, H. L. Hillege, D. J. Van Veldhuisen and W. H. Van Gilst	- 2013	Incidence and epidemiology of new onset heart failure with preserved vs. reduced ejection fraction in a community-based cohort: 11-year follow-up of PREVEND	review	European Heart Journal
Van Empel, V. and H. P. Brunner-La Rocca	- 2015	Inflammation in HFpEF: Key or circumstantial?	review	International Journal of Cardiology
Da Silva, D. M., H. Langer and T. Graf	- 2019	Inflammatory and molecular pathways in heart failure-ischemia, HFpEF and transthyretin cardiac amyloidosis	review	International Journal of Molecular Sciences
Yagel, S., S. M. Cohen and D. Goldman-Wohl	- 2022	An integrated model of preeclampsia: a multifaceted syndrome of the maternal cardiovascular-placental-fetal array	review	Am J Obstet Gynecol
Anderson, W. L., M. H. Bahrami, M. Guglin and R. Rao	- 2021	Lymphocytic myocarditis with suspected granulomatosis with polyangiitis presenting as cardiogenic shock, restrictive cardiomyopathy and complete heart block	review	Journal of Cardiology Cases
Barry, W. H.	- 1994	Mechanisms of immune-mediated myocyte injury	review	Circulation
Forte, M., D. Rodolico, P. Ameri, D. Catalucci, C. Chimenti, L. Crotti, L. Schirone, A. Pingitore, D. Torella, G. Iacovone, V. Valenti, G. G. Schiattarella, C. Perrino and S. Sciarretta	- 2023	Molecular mechanisms underlying the beneficial effects of exercise and dietary interventions in the prevention of cardiometabolic diseases	review	Journal of Cardiovascular Medicine
Liu, S., Y. Y. Chirkov and J. D. Horowitz	- 2018	Neutrophil-Initiated Myocardial Inflammation and Its Modulation by B-Type Natriuretic Peptide: A Potential Therapeutic Target	review	Int J Mol Sci
Cai, Z., C. Wu, Y. Xu, J. Cai, M. Zhao and L. Zu	- 2023	The NO-cGMP-PKG Axis in HFpEF: From Pathological Mechanisms to Potential Therapies	review	Aging and Disease
Corker, A., L. S. Neff, P. Broughton, A. D. Bradshaw and K. Y. Deleon-Pennell	- 2022	Organized chaos: Deciphering immune cell heterogeneity’s role in inflammation in the heart	review	Biomolecules
Suvakov, S., E. Bonner, V. Nikolic, D. Jerotic, T. P. Simic, V. D. Garovic, G. Lopez-Campos and L. McClements	- 2020	Overlapping pathogenic signalling pathways and biomarkers in preeclampsia and cardiovascular disease	review	Pregnancy Hypertension
Ermert, L., F. Kreimer, D. R. Quast, A. Pflaumbaum, A. Mügge and M. Gotzmann	- 2023	Rate of atrial fibrillation and flutter induced tachycardiomyopathy in a cohort of hospitalized patients with heart failure and detection of indicators for improved diagnosis	review	Frontiers in Cardiovascular Medicine
Yoo, J. H., S. W. Park, J. E. Jun, S. M. Jin, K. Y. Hur, M. K. Lee, M. Kang, G. Kim and J. H. Kim	- 2021	Relationship between low skeletal muscle mass, sarcopenic obesity and left ventricular diastolic dysfunction in Korean adults	review	Diabetes/Metabolism Research and Reviews
Turkoglu, E. I. and E. C. Kircicegi Cıcekdag	- 2019	Resistant hypertension in elderly: a clinical manifestation of heart failure with preserved ejection fraction? retrospective single-center analysis	review	Clinical and Experimental Hypertension

Bermea, K., A. Bhalodia, A. Huff, S. Rousseau and L. Adamo	- 2022	The Role of B Cells in Cardiomyopathy and Heart Failure	review	Curr Cardiol Rep
Sherwood, M. and T. M. Brown	- 2012	The role of cardiac rehabilitation in heart failure patients	review	US Cardiology
Godo, S., J. Takahashi, S. Yasuda and H. Shimokawa	- 2021	Role of inflammation in coronary epicardial and microvascular dysfunction	review	European Cardiology Review
Owens, W. A., A. Walaszczyk, I. Spyridopoulos, E. Dookun and G. D. Richardson	- 2021	Senescence and senolytics in cardiovascular disease: Promise and potential pitfalls	review	Mechanisms of Ageing and Development
Lau, E. S., A. Binek, S. J. Parker, S. H. Shah, M. V. Zanni, J. E. Van Eyk and J. E. Ho	- 2022	Sexual Dimorphism in Cardiovascular Biomarkers: Clinical and Research Implications	review	Circulation Research
Olsen, M. B., I. Gregersen, Ø. Sandanger, K. Yang, M. Sokolova, B. E. Halvorsen, L. Gullestad, K. Broch, P. Aukrust and M. C. Louwe	- 2022	Targeting the Inflammasome in Cardiovascular Disease	review	JACC Basic Transl Sci
Yeh, J. K. and C. Y. Wang	- 2016	Telomeres and telomerase in cardiovascular diseases	review	Genes
Prattichizzo, F., C. Frigé, R. La Grotta and A. Ceriello	- 2023	Weight variability and diabetes complications	review	Diabetes Research and Clinical Practice
Chaar, D., B. L. Dumont, B. Vulesevic, P. E. Neagoe, A. Räkel, M. White and M. G. Sirois	- 2022	Neutrophils and Circulating Inflammatory Biomarkers in Diabetes Mellitus and Heart Failure With Preserved Ejection Fraction	Unreported HR value	Am J Cardiol
Colluoglu, T. and Y. Akın	- 2023	The Value of Neutrophil-to-Lymphocyte Ratio and Epicardial Adipose Tissue Thickness in Heart Failure With Preserved Ejection Fraction	Unreported HR value	Cureus

1.4 Supplementary Figure1 sensitivity analysis

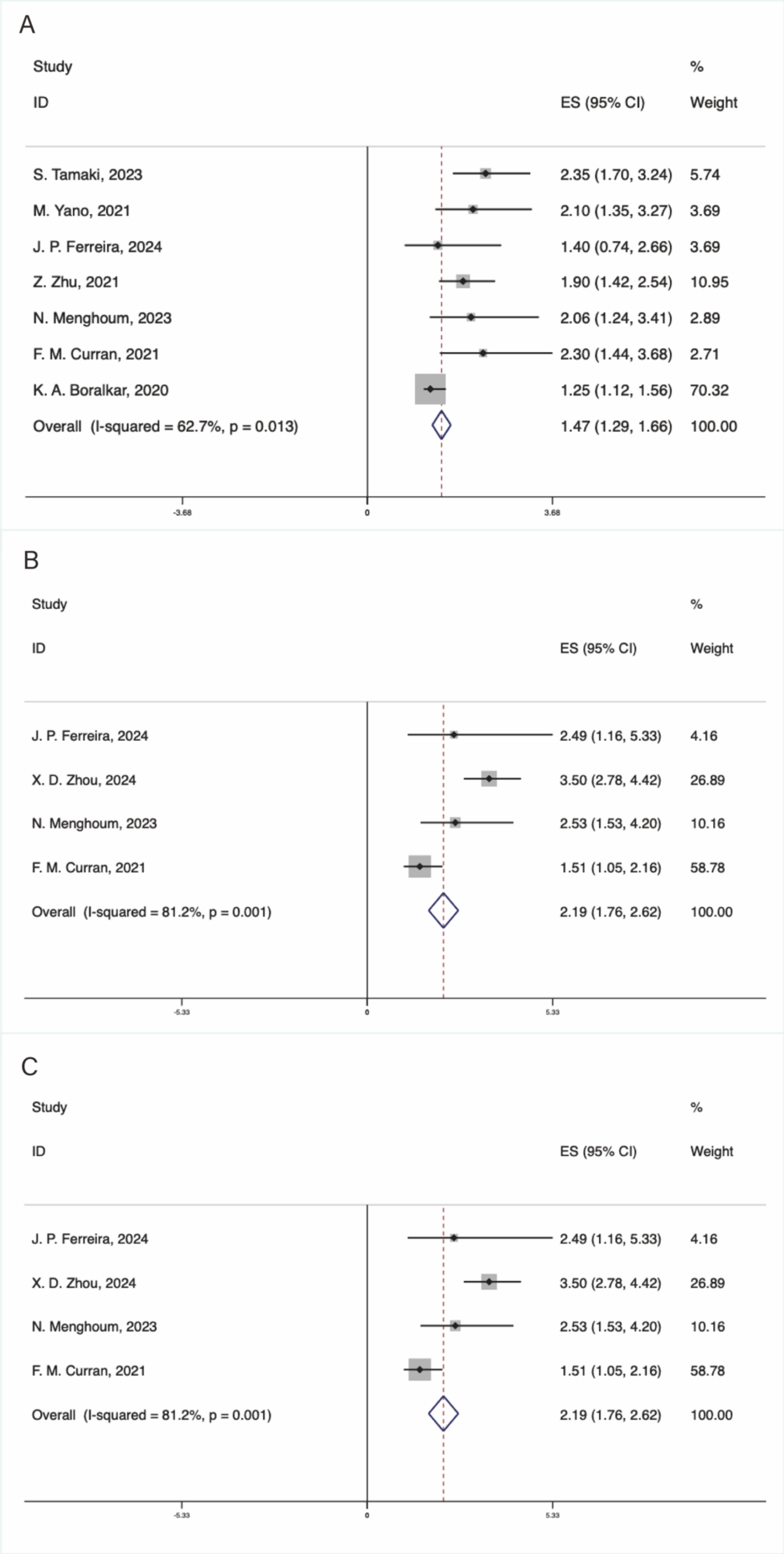
A
Study
ID
ES (95% CI)
%
Weight
S. Tamaki, 2023
2.35 (1.70, 3.24)
5.74
M. Yano, 2021
2.10 (1.35, 3.27)
3.69
J. P. Ferreira, 2024
1.40 (0.74, 2.66)
3.69
Z. Zhu, 2021
1.90 (1.42, 2.54)
10.95
N. Menghoum, 2023
2.06 (1.24, 3.41)
2.89
F. M. Curran, 2021
2.30 (1.44, 3.68)
2.71
K. A. Boralkar, 2020
1.25 (1.12, 1.56)
70.32
Overall (I-squared = 62.7%, p = 0.013)
1.47 (1.29, 1.66)
100.00
-3.68
0
3.68
B
Study
ID
ES (95% CI)
%
Weight
J. P. Ferreira, 2024
2.49 (1.16, 5.33)
4.16
X. D. Zhou, 2024
3.50 (2.78, 4.42)
26.89
N. Menghoum, 2023
2.53 (1.53, 4.20)
10.16
F. M. Curran, 2021
1.51 (1.05, 2.16)
58.78
Overall (I-squared = 81.2%, p = 0.001)
2.19 (1.76, 2.62)
100.00
-5.33
0
5.33
C
Study
ID
ES (95% CI)
%
Weight
J. P. Ferreira, 2024
2.49 (1.16, 5.33)
4.16
X. D. Zhou, 2024
3.50 (2.78, 4.42)
26.89
N. Menghoum, 2023
2.53 (1.53, 4.20)
10.16
F. M. Curran, 2021
1.51 (1.05, 2.16)
58.78
Overall (I-squared = 81.2%, p = 0.001)
2.19 (1.76, 2.62)
100.00
-5.33
0
5.33

frontiers
